# An alternative C–P cross-coupling route for the synthesis of novel V-shaped aryldiphosphonic acids

**DOI:** 10.3762/bjoc.18.160

**Published:** 2022-11-07

**Authors:** Stephen J I Shearan, Enrico Andreoli, Marco Taddei

**Affiliations:** 1 Energy Safety Research Institute, Swansea University, Fabian Way, Crymlyn Burrows, Skewen, Swansea SA1 8EN, UK; 2 Dipartimento di Chimica e Chimica Industriale, Università di Pisa, Via Giuseppe Moruzzi, 13, 56124 Pisa, Italy

**Keywords:** arylphosphonic acids, cross-coupling reaction, phosphonate esters, transition-metal catalysis

## Abstract

The synthesis of phosphonate esters is a topic of interest for various fields, including the preparation of phosphonic acids to be employed as organic linkers for the construction of metal phosphonate materials. We report an alternative method that requires no solvent and involves a different order of addition of reactants to perform the transition-metal-catalyzed C–P cross-coupling reaction, often referred to as the Tavs reaction, employing NiCl_2_ as a pre-catalyst in the phosphonylation of aryl bromide substrates using triisopropyl phosphite. This new method was employed in the synthesis of three novel aryl diphosphonate esters which were subsequently transformed to phosphonic acids through silylation and hydrolysis.

## Introduction

Phosphonates and phosphonic acids are a very interesting class of compounds and examples of their use can be found in a number of different areas, including pharmaceuticals [1–6], metal chelation [7–9], anti-corrosion coatings [10–12], fertilizers [13–14], proton conduction [15–17], and catalysis [18], amongst others. Phosphonates can also be employed as organic linkers in combination with metal ions to afford coordination polymers and metal-organic frameworks (MOFs), or more aptly, metal phosphonate frameworks [19–20]. One of the main challenges in the synthesis of metal phosphonates is that the linkers are rarely commercially available and can often be difficult to prepare. Most often, the challenge is, in fact, not the synthesis of the phosphonic acid itself, but that of the phosphonic ester precursor [21].

Perhaps the most well-known C–P coupling procedure is the Michaelis–Arbuzov rearrangement involving a reaction between a primary alkyl halide and a trialkyl phosphite, first reported in the late 1890s, the general scheme for which can be seen in Supporting Information File 1, Scheme S1 [22]. It should be noted that this reaction is not suitable for use with aryl halide substrates due to the poor reactivity between aryl halides and trialkyl phosphites [23]. Some of the most studied C–P coupling reactions involving aryl substrates are those employing catalysts, which are required in order to lower the energy barrier of the reaction and overcome the poor reactivity between aryl halides and trialkyl phosphites [24–26]. These catalytic cross-coupling reactions tend to follow similar pathways to the Michaelis–Arbuzov reaction, with the inclusion of a catalytic intermediate step. A number of suitable catalysts have been identified, ranging from nickel(II) bromide and nickel(II) chloride, to palladium(II) acetate and palladium(II) chloride [23,27–28]. Reactions involving these catalysts are most often carried out at high temperatures, usually in excess of 160 °C, and involve slow dropwise addition of the trialkyl phosphite to the substrate [23]. In the search for milder reaction conditions, a new catalyst, tetrakis(triphenylphosphine)palladium(0), was introduced by Hirao and co-workers, which allowed for the lowering of the reaction temperature to approximately 90 °C [29–31].

In this work, we have developed an alternative experimental protocol to perform the Ni-catalyzed C–P cross-coupling reaction, or Tavs reaction, whose mechanism is shown in Supporting Information File 1, Scheme S2 [32]. Starting from commercially available bromide precursors, we target a series of novel aryldiphosphonic acids sharing the feature of having non-linear – or V-shaped – geometry, intended to be employed as organic linkers for the synthesis of open framework metal phosphonate materials. The proposed protocol does not require a solvent, while featuring reaction times and yields comparable, if not better, to those of most procedures commonly employed in the literature.

## Results and Discussion

**Scheme 1:**
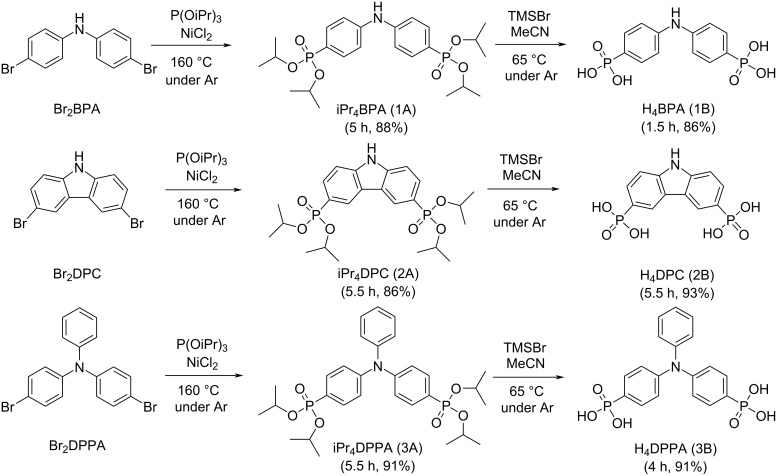
Scheme showing the transformation of the Br-substrates to phosphonate esters and then to phosphonic acids.

The goal of this work is the phosphonylation of the commercially available bromo-substituted *N*-aryl precursors bis(4-bromophenyl)amine (Br_2_BPA), 3,6-dibromocarbazole (Br_2_DPC), and 4-bromo-*N*-(4-bromophenyl)-*N*-phenylaniline (Br_2_DPPA) (see Scheme 1). In light of using the resulting phosphonic acids as linkers for the construction of metal phosphonate frameworks, two main considerations were made when selecting these substrates with regards to rigidity and geometry (Figure S1, Supporting Information File 1). In the compounds considered here, rigidity is ensured by the network of sp^2^-hybridized carbon atoms, or aromatic rings, and is important to ensure stability in the potential MOF structures derived from the proposed linkers. The geometry of these linkers, termed as V-shaped, was selected to try and move away from the pillared-layered structures that are obtained when using linear diphosphonate linkers, which are often either non-porous or have low porosity and have little to no long-range order. The idea here was that the V-shaped linkers, as well as different substituents attached to nitrogen, could potentially force a non-layered porous structure.

Conventionally, the transition-metal-catalyzed C–P cross-coupling reaction is carried out by placing the aryl halide and the precatalyst into a round-bottomed flask in the presence of a suitable solvent, such as 1,3-diisopropylbenzene or mesitylene, and setting to reflux. The advantage of using such solvents lies in their high boiling point (203 °C and 164 °C, respectively), which allows for reactions to be run at high temperatures, thus enhancing the rate of the reaction. While the reaction mixture is refluxing, the alkyl phosphite is added in several small portions.

The work we present differs from the conventional nickel-catalyzed cross-coupling reaction in two aspects: we use no solvent and we employ a different order of addition of reactants. The absence of solvent presents a few advantages over the original method. First of all, the removal of said solvent by distillation after the completion of the reaction is no longer required and thus there is a simplification of the workup procedure. Second, there is no dilution of the reaction mixture, which lends itself to an increased reaction rate. Contrary to the conventional method, this alternative method starts with the nickel(II) precatalyst and the alkyl phosphite, triisopropyl phosphite in our case, being added to a round-bottomed flask and heated to approximately 160 °C, leading to the formation of the nickel(0) catalyst, more accurately representing the catalytic cycle presented in Scheme S2 (Supporting Information File 1). The solid aryl bromide is then added to the mixture via a powder addition funnel over a 2–4 hour period, depending on the substrate, and is then left to react for an additional 1–3.5 hours to reach complete conversion of the substrate into the respective phosphonic ester. In this way, the dibromide substrate is always the limiting reagent, promoting full conversion to the respective diphosphonic ester and limiting the accumulation of an undesired, partially converted product that would need to be separated during workup.

**Figure 1:**
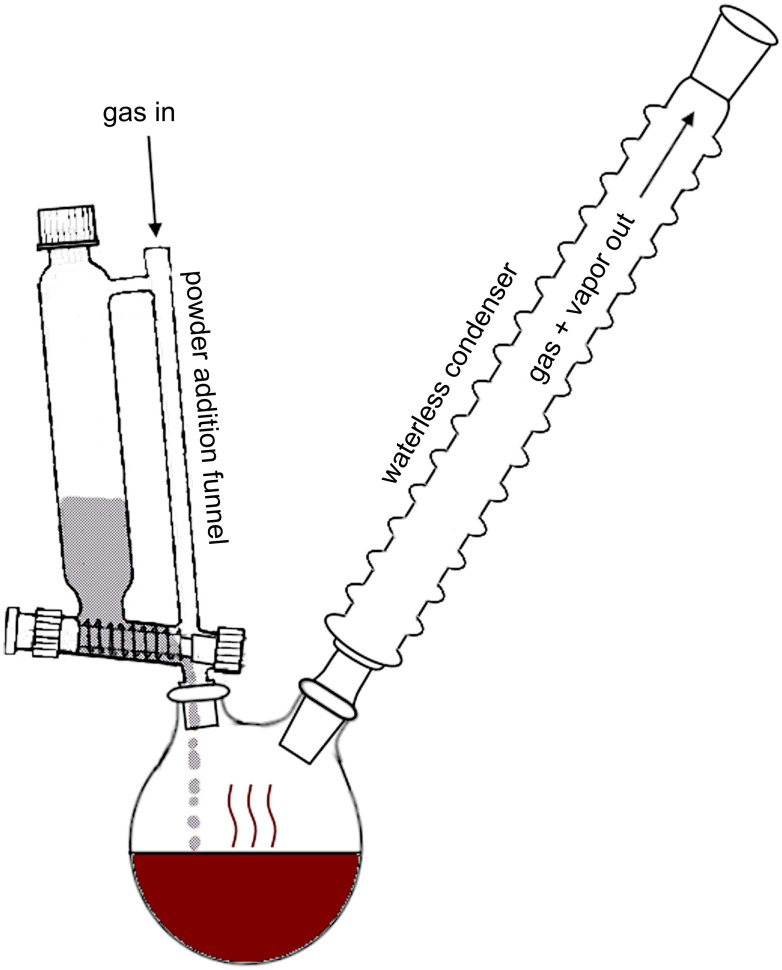
Experimental setup for the improved C–P cross-coupling reaction.

Figure 1 shows the setup for the reaction, with the solid aryl bromide in grey and the precatalyst/triisopropyl phosphite mixture in red. It is important to note here that the system is kept under a constant flow of either argon or nitrogen, mainly to avoid side reactions with components in the air (humidity, oxygen), but also to prevent the solid in the addition funnel from contacting any vapour and turning soggy before it is added to the round-bottomed flask. As can be seen in Figure 1, this is achieved by flowing the gas through the powder addition funnel via a gas inlet. This also allows for the quick removal (usually complete in less than 30 minutes) of residual triisopropyl phosphite at the end of the reaction by simply increasing the gas flow, thus preventing the equilibrium between the gas and liquid phases and allowing to bypass the further step which would have involved removing these components by vacuum distillation. Although not shown in Figure 1, the addition of a second condenser and collection flask perpendicular, as in a distillation, to the first column also allows for the collection of unreacted phosphite, and byproducts, such as isopropyl bromide. Firstly, this prevents any release of vapours of toxic compounds and facilitates appropriate disposal procedures. Secondly, it is likely that the majority of what remains in the flask at the end is simply unreacted phosphite, which would ideally need to be investigated to assess its recyclability, and lead to a process with greener attributes. In this sense, the phosphite is likely to be the last product coming over via distillation, and should be relatively pure, but further investigation would be required in order to confirm this.

The choice of the phosphite is also important, partially due to the boiling point and the potential for running reactions at higher temperatures, and also the formation of an alkyl halide byproduct. It is the reactivity of this byproduct that determines which phosphite is chosen. In this case, triisopropyl phosphite has been chosen over commonly employed triethyl phosphite, since the latter results in a more reactive alkyl halide (i.e., ethyl bromide), which would react with triethyl phosphite to produce diethyl ethylphosphonate, thus consuming the triethyl phosphite in a competing reaction and introducing undesired side products that would make the workup procedure more laborious. Furthermore, the boiling point of triisopropyl phosphite is 181 °C, versus 156 °C for triethyl phosphite, which allows to run the reaction at higher temperature and reduce the time.

In Table 1, we see a range of different methods based on cross-coupling reactions compared to the method proposed in this work. The first, and one of the most important comparisons, is time. The upper range for our method is in the 5.5 hour mark, whereas most of the literature routes run for at least 6.5 hours. The shorter time for method F is likely due to the fact that it involves a monobromo substrate, with no issues related with the presence of partially converted side products, which are common with polybromo substrates. The shorter time for method H is due to the much higher temperature at which the reaction can be run in a pressure-resistant closed vessel. The relatively short time, in part, can be attributed to the absence of solvent, which we cited previously as an advantage in that we are not diluting the reaction mixture and thus not slowing down the reaction. This in turn explains the high phosphite-to-bromine ratio, which in our case is higher than all the other routes, since the phosphite itself acts as the solvent as well as being a reactant. If this ratio was lower, there would be a considerable drop in the reaction rate towards the end and would likely lead to generally lower yields. This issue could be further minimized upon exploration of recycling the phosphite distillate. It is to be noted that our method, by bypassing the distillation step necessary to remove the solvent in most literature methods, in just 30 minutes on top of the time reported in Table 1 provides a reaction crude that can be then worked up to obtain the desired product. We also manage to use less catalyst than some of the other methods, except for methods F, G, and H. In keeping with the mild conditions, the temperature we use is 160 °C, which is lower than that of the other reactions. Working at 180 °C, close to the boiling point of triisopropyl phosphite, was actually detrimental to the reaction. In these conditions, under a constant stream of inert gas, the phosphite is prone to be swept away, even in the presence of a reflux condenser. Reactions carried out in such conditions afforded a very viscous crude and a low conversion of the starting material, as revealed by TLC.

**Table 1:** A comparison of literature phosphonate syntheses with the alternative method proposed in this work. The microwave-assisted method made use of a pressure-resistant vessel due to considerable pressure buildup (≈ 10 bar), while the other methods were run under reflux conditions.

	this work	method A [33]	method B [34]	method C [35]	method D [36]

time (h)	4–6	20	20	20	54
scale (g of precursor)	3–5	30	8	10	6.3
temperature (°C)	160	180	180	170	185
P/Br ratio	7	1.5	3	2.1	4.5
mol %/Br (NiX_2_)	13% X = Cl	17% X = Br	39% X = Cl	16% X = Br	15% X = Cl
isolated yield (%)	70–90	60	89	61	80
solvent	no solvent	1,3-diisopropyl- benzene	1,3-diisopropyl- benzene	*tert*-butylbenzene	1,3-diisopropyl- benzene
procedure	addition of Br-substrate	addition of phosphite	addition of phosphite	addition of phosphite	addition of phosphite
substrate	dibromo- polyarylamines	1,3,5-tris(4-bromo- phenyl)benzene	2,4,6-tri-(4-bromo- phenyl)-s-triazine	tetra(4-bromo- phenyl)methane	4,4-dibromo- biphenyl

	this work	method E [37]	method F [38]	method G [39]	method H [40]

time (h)	4–6	29.5	6.5	3	0.75
scale (g of precursor)	3–5	19.5	4.5	23	0.543
temperature (°C)	160	180	180	180	225
P/Br ratio	7	1.6	1.5	1.5	5
mol %/Br (NiX_2_)	13% X = Cl	6% X = Br	7% X = Br	8% X = Br	5% X = Cl
isolated yield (%)	70–90	78	85	80	82
solvent	no solvent	1,3-diisopropyl- benzene	mesitylene	mesitylene	no solvent
procedure	addition of Br-substrate	addition of phosphite	addition of phosphite	addition of phosphite	one-pot synthesis (microwave assisted)
substrate	dibromo- polyarylamines	1,3-dibromo- benzene	2,5-dibromo- thiophene	methyl 3-bromo- benzoate	1,3,5-tris(4-bromo- phenyl)benzene

Notably, the yield we achieve, which varies between substrates, is generally comparable to those of literature routes. With regards to method H, we note that the scale of this method, originally reported by one of us, was limited to 0.543 g (1 mmol) of substrate. Scale up of this protocol was not attempted, but it might become problematic due to issues with microwave penetration in a medium that contains a strong absorber, such as the Ni catalytic complex. In this work, we have employed either 5.0 g (15.3 mmol), 4.0 g (12.2 mmol) or 3.0 g (7.4 mmol) of substrate.

Once the phosphonate esters had been successfully obtained and characterized by ^1^H, ^31^P, ^13^C NMR and mass spectrometry (see experimental section and Supporting Information File 1), they were then subjected to silylation and subsequent hydrolysis using the method put forward by McKenna et al. (1977), which involves the use of trimethylbromosilane (TMSiBr) in a transesterification of the dialkyl phosphonate to bis(trimethylsilyl) phosphonate, followed by treatment in water or short-chain alcohols to obtain a phosphonic acid, as shown in Supporting Information File 1, Scheme S3 [41–42]. Prior to using this method, the standard hydrolysis under prolonged reflux in 6 M HCl was attempted, though these conditions proved too harsh, and often led to cleavage of the C–P bond. Thus, this popular method was abandoned in favor of using the less harsh method employing TMSiBr, which most often led to achieve overall yields above 70% for the phosphonic acid, based on the initial Br-substrate.

## Conclusion

Presented in this article is the synthesis of three novel phosphonate esters and their corresponding phosphonic acids. While the phosphonic acids are indeed the target products, the progress made here is mainly focused on the improvement of the cross-coupling procedure used to obtain the phosphonate esters. Oftentimes, these reactions take up to 24 hours to reach completion, sometimes more, while here we have presented a simple yet effective change that can be made to the order of addition of reactants, which affords a reaction time that is at least five times faster than most literature methods with no considerable effect on the yield or the purity of the product. This has also completely removed the requirement of a solvent, since triisopropyl phosphite acts as the solvent. In making savings for both cost of reagents and in total reaction time, and with no detriment to the yield, it is clear that this method presents an advantage over the literature routes, both in terms of cost and efficient use of time. Referring specifically to the phosphonic acids presented in this work, we have obtained three novel and structurally related linkers for the preparation of metal phosphonates. Each of the linkers was obtained in good yield and with no considerable impurities identified during characterization. This series of linkers will allow to determine the effects of the geometry and of different substituents on the formation of metal phosphonate frameworks.

## Supporting Information

File 1 NMR Spectra, MS spectra, and respective discussions.
