# Supplementary material for: An alternative C–P cross-coupling route for the synthesis of novel V-shaped aryldiphosphonic acids
Source: Beilstein J Org Chem. 2022 Nov 7;18:1518–23. doi: 10.3762/bjoc.18.160 (PMC9663968; doi:10.3762/bjoc.18.160)
Supplement: File 1 — NMR Spectra, MS spectra, and respective discussions. [file Beilstein_J_Org_Chem-18-1518-s001.pdf]

BEILSTEIN JOURNAL OF ORGANIC CHEMISTRY

Supporting Information

for

An alternative C–P cross-coupling route for the synthesis of novel V-shaped aryldiphosphonic acids

Stephen J. I. Shearan, Enrico Andreoli and Marco Taddei

Beilstein J. Org. Chem. **2022,** *18,* 1518–1523. doi:10.3762/bjoc.18.160

NMR Spectra, MS spectra, and respective discussions

License and Terms: This is a supporting information file under the terms of the Creative Commons Attribution License (https://creativecommons.org/licenses/by/4.0). Please note that the reuse, redistribution and reproduction in particular requires that the author(s) and source are credited and that individual graphics may be subject to special legal provisions.
The license is subject to the *Beilstein Journal of Organic Chemistry* terms and conditions: (https://www.beilstein-journals.org/bjoc/terms)

$$RCH_2X \quad + \quad P(OR^1)_3 \longrightarrow RCH_2P(O)(OR^1)_2 + \quad R^1X$$

Scheme S1: General scheme for the Michaelis–Arbuzov reaction. This reaction proceeds in two steps, initiating when the α-carbon of the primary alkyl halide undergoes a nucleophilic attack from the phosphorus lone pair of the trialkyl phosphite, leading to the formation of a quasi-phosphonium salt. In the second step, the α-carbon on one of the three alkoxy groups undergoes nucleophilic attack by the free halide ion generated in the first step, resulting in the formation of a new C–X bond and the cleavage of the C–O bond.

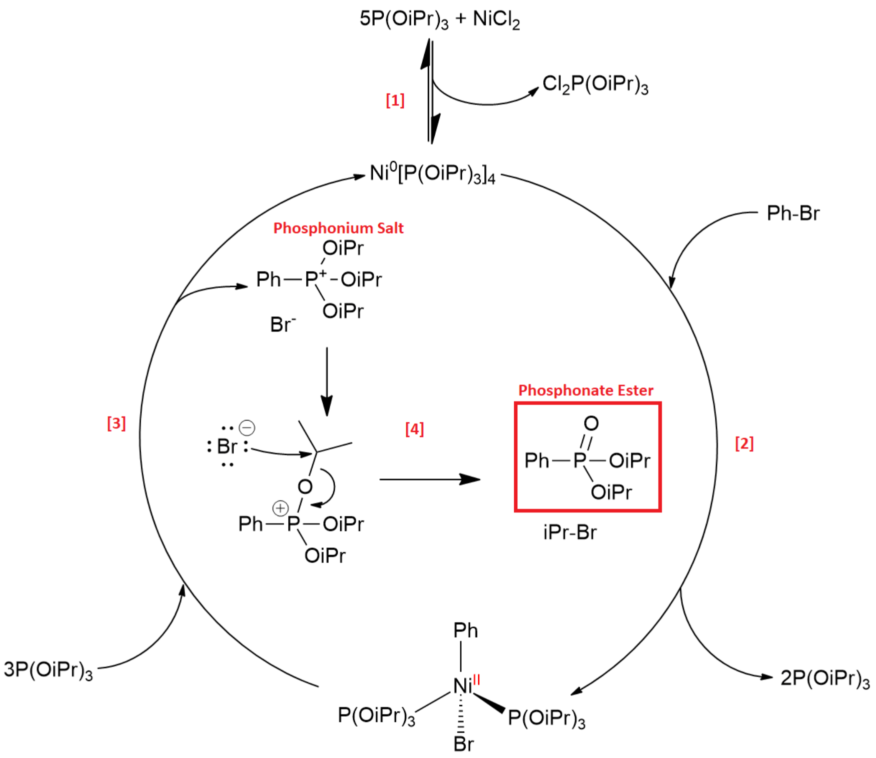

Scheme S2: The catalytic cycle for the nickel-catalyzed cross-coupling reaction of aryl halides with triisopropyl phosphite. The cycle begins with the reduction of the nickel(II) chloride pre-catalyst by the trialkyl phosphite (shown here as triisopropyl phosphite) to form the catalyst, tetrakis(triisopropyl phosphite)nickel(0) [Step 1]. The aryl halide then undergoes oxidative addition to the nickel complex, forming a new nickel(II) complex [Step 2]. We then see the formation of the arylphosphonium salt and the regeneration of the nickel(0) catalyst *via* eliminative reduction [Step 3]. The final step in the cycle is identical to the Michaelis–Arbuzov reaction, whereby diisopropyl phosphonate and isopropyl bromide are formed through a nucleophilic substitution of the halide anion [Step 4].

Scheme S2 was adapted with permission of The Royal Society of Chemistry from [18] (“Chapter 6: Synthesis of Phosphonic Acids and Their Esters as Possible Substrates for Reticular Chemistry. In Metal Phosphonate Chemistry: From Synthesis to

Applications” by J. Zon et al., © 2011); permission conveyed through Copyright Clearing Center, Inc. This content is not subject to CC BY 4.0.

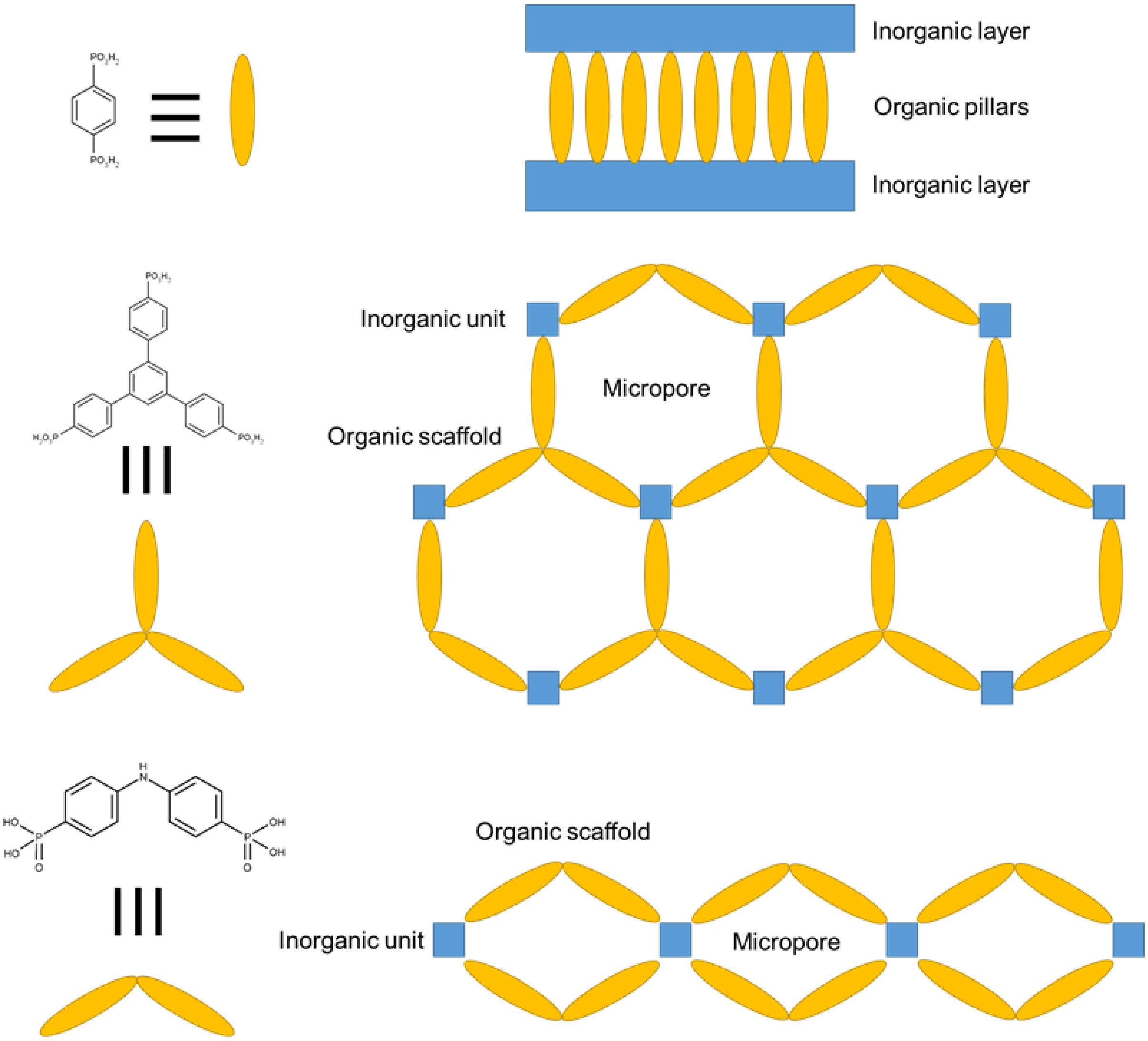

Figure S1: Linker design strategy to induce the formation of microporous metal phosphonates. Linear diphosphonates, such as 1,4-phenylenediphosphonic acid (top), usually afford dense pillared-layered structures with little or no empty volume available. The use of rigid tritopic linkers, such as 1,3,5-tris(4-phosphonophenyl)benzene (center), which do not fit in the pillared-layered motif, has led to several examples of open framework metal phosphonates [1]. Similarly, we expect that rigid, V-shaped linkers, such as *N*,*N*-bis(4-phosphonophenyl)amine

(bottom), can exert a similar effect to tritopic linkers, favoring the assembly of a microporous structure.

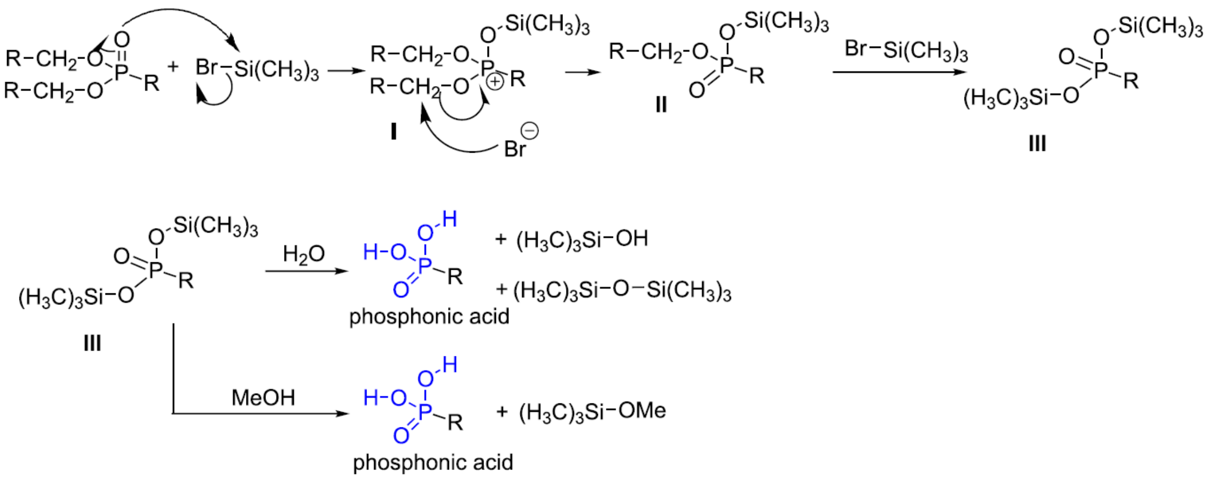

Scheme S3: Proposed mechanism for the hydrolysis of dialkyl phosphonates using trimethylbromosilane. The initial step in this mechanism proceeds via an oxophilic substitution on the silicon of TMSiBr, whereby bromide acts as the leaving group, resulting in the formation of intermediate I. A nucleophilic attack by the bromide on the electrophilic carbon then leads to the formation of intermediate II, and then intermediate III through repetition of the same process. From here, there are two possible routes for obtaining a phosphonic acid. The first route is hydrolysis, leading to the formation of the phosphonic acid and two volatile side products, trimethylsilanol and hexamethyldisiloxane. The second route is methanolysis, leading to the formation of the phosphonic acid and methoxytrimethylsilane, a side product that is inherently more volatile than those formed during hydrolysis. Here we followed the water hydrolysis route. Scheme 4 was adapted from [2] (© 2017 C. M. Sevrain et al., published by Beilstein-Institut, distributed under the terms of the Creative Commons Attribution 4.0 International License, https://creativecommons.org/licenses/by/4.0).

Experimental

Materials

All materials were used as received and not subject to further purifications.

- Acetonitrile, anhydrous (75-05-8, 99.8%, CH_3CN, Sigma-Merck)
- Bis(4-bromophenyl)amine (16292-17-4, 97%, $C_{12}H_9Br_2N$, Sigma-Merck)
- 4-Bromo-*N*-(4-bromophenyl)-*N*-phenylaniline (81090-53-1, 98%, $C_{18}H_{13}Br_2N$, Fluorochem)
- 3,6-Dibromo-9*H*-carbazole (6825-20-3, 97%, $C_{12}H_7Br_2N$, Fluorochem)
- Ethyl acetate (141-78-6, 99.7%, $CH_3CO_2C_2H_5$, Sigma-Merck)
- Hexane (110-54-3, 97%, $CH_3(CH_2)_4CH_3$, Sigma-Merck)
- Nickel(II) chloride, anhydrous (7718-54-9, 98%, $NiCl_2$, Alfa Aesar)
- Triisopropyl phosphite (116-17-6, 95%, $[(CH_3)_2CHO]_3P$, Sigma-Merck)
- Trimethylbromosilane (2857-97-8, 97%, $(CH_3)_3SiBr$, Sigma-Merck)

Methods

^{1}H, ^{13}C, ^{31}P, and HSQC NMR spectra were recorded on a Bruker Avance III 500 MHz instrument. Phosphonate esters were dissolved in $CDCl_3$. Phosphonic acids were dissolved in a 0.1 M solution of NaOH in D_2O. ^{1}H NMR parameters: 16 scans, 5 s relaxation delay (d1). ^{31}P NMR parameters: 32 scans, 2 s relaxation delay (d1). ^{13}C NMR parameters: 1024 scans, 2 s relaxation delay (d1). ^{1}H-^{13}C HSQC: 2 scans, 2 s relaxation delay (d1).

All mass spectral analyses were carried out at the National Mass Spectrometry Facility (NMSF), Swansea University Medical School, and processed using vendor XCalibur

software. iPr_4BPA, iPr_4DPC, and iPr_4DPPA samples were prepared for analysis by solvation in 350 μL MeOH and further 1:1000 dilution in MeOH with 30 mM ammonium acetate (NH_4OAc). 20 μL was aliquoted into a 96 well plate and sprayed via an Advion NanoMate in positive ion mode at +1.5 kV into the API source of a Thermo LTQ Orbitrap XL. API source conditions were capillary temperature 200 °C, capillary voltage 41 V and tube lens voltage 150 V. H_4BPA, H_4DPC, and H_4DPPA samples were prepared for analysis by solvation in 350 μL MeOH and further 1:1000 dilution in MeOH with 1% diethylamine (DEA) to promote deprotonation. 20 μL was aliquoted into a 96 well plate and sprayed via an Advion NanoMate in negative ion mode at −1.5 kV into the API source of a Thermo LTQ Orbitrap XL. API source conditions were capillary temperature 200 °C, capillary voltage −32 V and tube lens voltage −100 V.

TM-Catalyzed C–P coupling reactions

(1A) *N*,*N*-Bis(4-diisopropylphosphonophenyl)amine [iPr_4BPA]

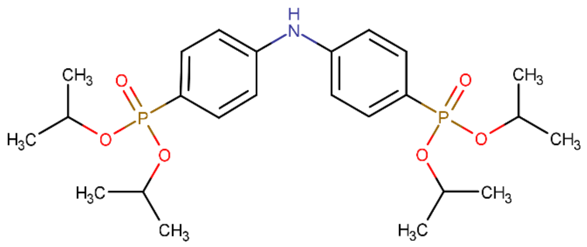

Figure S2: Chemical structure of iPr_4BPA (1A).

Bis(4-bromophenyl)amine (4.0 g, 12.2 mmol) was placed into a screw powder addition funnel and attached to a 100 mL round-bottomed flask. Triisopropyl phosphite (42 mL, 170 mmol, 7 equivalents) and anhydrous nickel chloride (13 mol % per Br – 0.41 g, 3.16 mmol) were then added to the round-bottomed flask and set to reflux (160 °C) under argon. Once the mixture had reached temperature, the bis(4-bromophenyl)amine was added slowly over 4 hours and the reaction monitored via

TLC using an acetone/ethyl acetate mixture in a 1:9 ratio. Once the addition was complete, the reaction mixture was left for a further 1 hour and again monitored by TLC to identify when the reaction had gone to completion. The gas flow rate was increased in order to remove excess phosphite and remaining byproducts, resulting in a dark treacle-like substance. This was left to cool and subsequently washed overnight in hexane, resulting in the formation of a fine white powder. This was washed two more times in hexane and separated via centrifugation. The solid was then purified using flash chromatography with a 50:50 mixture of acetone and ethyl acetate and allowed to recrystallized through solvent evaporation, resulting in 5.35 g of white solid. (Yield = 88.0%)

^{31}P NMR (202 MHz, $CDCl_3$): δ 17.31 (m, J ≈ 4.4 Hz, 2P)

^{1}H NMR (500 MHz, $CDCl_3$): δ 7.71 (dd, J = 12.6, 8.4 Hz, 4H, aromatic), δ 7.19 (dd, J = 8.5, 3.1 Hz, 4H, aromatic), δ 4.68 (dp, J = 7.9, 6.1 Hz, 4H, O-C(H)-CH3), δ 1.31 (dd, 24H, O-C-CH3)

^{13}C NMR (500 MHz, $CDCl_3$): δ 145.13 (s, 2C), δ 133.50 (d, J = 10.95 Hz, 4C), δ 122.65 (s, 2C), δ 116.97 (s, 4C), δ 70.50 (d, J = 5.54 Hz, 4C), δ 24.00 (dd, J = 25.6, 4.4 Hz, 8C)

m/z: 498.22 ($[M+H]^+$), 995.43 ($[2M+H]^+$)

(2A) 3,6-Bis(diisopropylphosphono)-9*H*-carbazole [iPr$_4$DPC]

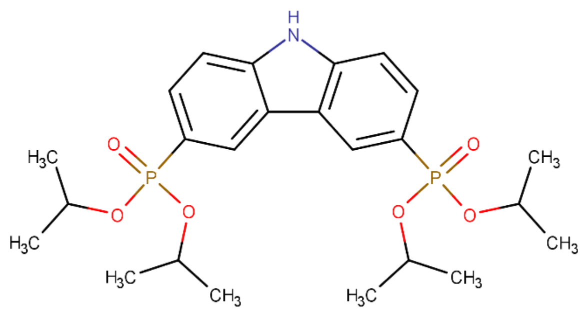

Figure S3: Chemical structure of iPr$_4$DPC (2A).

3,6-Dibromo-9*H*-carbazole (5.0 g, 15.4 mmol) was placed into a screw powder addition funnel and attached to a 100 mL round-bottomed flask. Triisopropyl phosphite (52.5 mL, 214 mmol, 7 equivalents) and anhydrous nickel chloride (13 mol % per Br – 0.52 g, 4.00 mmol) were then added to the round-bottomed flask and set to reflux (160 °C) under argon. Once the mixture in the round-bottom had reached temperature, the 3,6-dibromo-9*H*-carbazole was added slowly over 4.5 hours and monitored via TLC. Once the addition was complete, the reaction mixture was left for a further 1 hour and monitored by TLC to identify when the reaction had gone to completion. The gas flow was then increased to remove excess phosphite, resulting in a brown sticky crude. This was washed overnight in hexane, resulting in the formation of an off-white powder. This was washed two more times in hexane and the final white powder was dried using rotary evaporation, resulting in 6.56 g of white solid. (Yield = 86.1%)

^{31}P NMR (202 MHz, $CDCl_3$): δ 18.63 (s, 2P)

^{1}H NMR (500 MHz, $CDCl_3$): δ 8.95 (s, 1H, N-H), δ 8.66 (d, J = 15.1 Hz, 2H, aromatic), δ 7.91 (ddd, J = 12.4, 8.3, 1.4 Hz, 2H, aromatic), δ 7.55 (dd, J = 8.4, 3.1 Hz, 2H, aromatic), δ 4.75 (dp, J = 8.0, 6.2 Hz, 4H, CH), δ 1.35 (dd, J = 83.4, 6.2 Hz, 24H, O-C-(CH3)2)

^{13}C NMR (500 MHz, $CDCl_3$): δ 141.98 (s, 2C), δ 129.68 (d, J = 11.76 Hz, 2C), δ 125.40 (s, J = 10.87 Hz, 2C), δ 122.84 (s, 2C), δ 121.40 (s, 2C), δ 110.96 (d, J = 16.55 Hz, 2C), δ 70.66 (d, J = 5.36 Hz, 4C), δ 24.04 (dd, J = 25.9, 4.1 Hz, 8C)

m/z: 496.2 ($[M+H]^+$), 991.39 ($[2M+H]^+$)

(3A) 4-Diisopropylphosphono-*N*-(4-diisopropylphosphonophenyl)-*N*-phenylaniline [iPr$_4$DPPA]

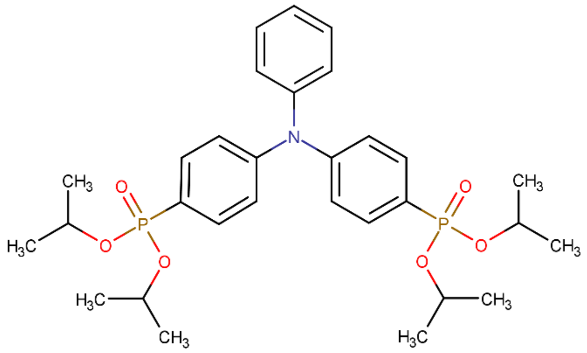

Figure S4: Chemical structure of iPr$_4$DPPA (3A).

4-Bromo-*N*-(4-bromophenyl)-*N*-phenylaniline (3.0 g, 7.4 mmol) was placed into a screw powder addition funnel and attached to a 100 mL round-bottomed flask. Triisopropyl phosphite (25.7 mL, 104.2 mmol, 7 equivalents) and anhydrous nickel chloride (13 mol % per Br - 0.25 g, 1.92 mmol) were then added to the round-bottomed flask and set to reflux (160 °C) under argon. Once the mixture in the round-bottom had reached temperature, the 4-bromo-*N*-(4-bromophenyl)-*N*-phenylaniline was added slowly over 2.5 hours and monitored via TLC. Once the addition was complete, the reaction mixture was left for a further 3 hours and monitored by TLC to identify when the reaction had gone to completion. After 3 hours, the gas flow rate was increased in order to remove excess phosphite and remaining byproducts. The mixture was then left to cool and became a sticky, treacle-like substance, and was subsequently washed overnight in hexane, resulting in the formation of a fine white solid. The hexane was decanted, and the powder again washed in hexane, resulting in 3.86 g of a white solid. (Yield = 90.5%)

^{31}P NMR (202 MHz, CDCl$_3$): δ 17.02 (m, 2P)

^{1}H NMR (500 MHz, CDCl$_3$): δ 7.68 (dd, J = 12.8, 8.2 Hz, 4H, Aromatic), δ 7.36 (t, J = 7.8 Hz, 2H, Aromatic), δ 7.20 (t, J = 7.5 Hz, 1H, Aromatic), δ 7.15 (d, J = 8.1 Hz, 2H,

Aromatic), δ 7.12 (dd, 4H, Aromatic), δ 4.73 (h, J = 6.6 Hz, 4H), δ 1.34 (dd, J = 55.1, 6.2 Hz, 24H).

^{13}C NMR (500 MHz, $CDCl_3$): δ 150.25 (s, 2C), δ 146.08 (s, 1C), δ 133.03 (d, J = 10.80 Hz, 4C), δ 129.84 (s, 2C), δ 126.54 (s, 2C), δ 125.27 (s, 2C), δ 123.95 (s, 1C), δ 122.46 (d, J = 15.5 Hz, 4C), δ 70.63 (d, J = 5.7 Hz, 4C), δ 70.63 (dd, J = 21.3, 4.4 Hz, 8C)

m/z: 574.2 ($[M+H]^+$)

Phosphonic acid synthesis through silylation and hydrolysis

(1B) *N*,*N*-Bis(4-phosphonophenyl)amine [H_4BPA]

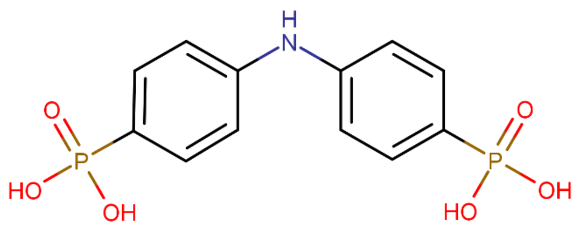

Figure S5: Chemical structure of H_4BPA (1B).

iPr_4BPA (4.5 g, 9.1 mmol) was dissolved in approximately 50 mL of acetonitrile inside a 100 mL round bottom flask and flushed with argon. Trimethylbromosilane (8.4 mL, 63.3 mmol) was then added to the flask, resulting in a color change of the solution to a blue color. The temperature of the vessel was then set to 65 °C. After one and a half hours, TLC showed that the starting material had already been consumed, thus the heating was turned off and the solution allowed to cool. Once sufficiently cooled, rotary evaporation was used to remove the solvent, acetonitrile, resulting in a blue oil. On treatment of this oil with water, a white solid began forming. Water was then progressively added until no oil remained. The white solid was then washed chloroform and dried using rotary evaporation, resulting in 2.56 g of white solid. (Yield = 86.1%)

^{31}P NMR (202 MHz, 0.1M NaOH in D_2O): δ 12.39 (s, 2P)

^{1}H NMR (500 MHz, 0.1M NaOH in D_2O): δ 7.50 (dd, J = 11.6, 8.1 Hz, 4H, aromatic), δ 7.05 (d, J = 8.6 Hz, 4H, aromatic)

^{13}C NMR (500 MHz, 0.1M NaOH in D_2O): δ 143.43 (s, 2C), δ 133.70 (s, 2C), δ 131.53 (d, J = 9.8 Hz, 4C), δ 116.81 (d, J = 13.3 Hz, 4C)

m/z: 163.5 ($[M\text{-}2H]^{2-}$), 163.5 ($[M\text{-}H]^{-}$)

(2B) 3,6-Diphosphono-9*H*-carbazole [H_4DPC]

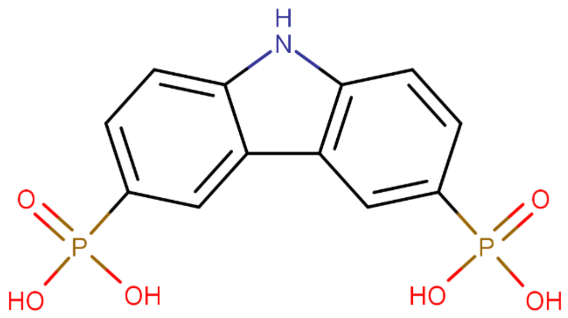

Figure S6: Chemical structure of H_4DPC (2B).

iPr$_4$DPC (3.0 g, 6.05 mmol) was partially dissolved in approximately 100 mL of acetonitrile inside a 250 mL round-bottomed flask and flushed with argon. Trimethylbromosilane (5.6 mL, 42.4 mmol) was then added to the flask, resulting in a color change of the solution to a blue color. The temperature was then set to 65 °C and left to react for five and a half hours and monitored via TLC. Once the starting material was consumed, the heating was turned off and the solution allowed to cool. Once sufficiently cooled, rotary evaporation was used to remove the solvent, acetonitrile, resulting in a blue oil. On treatment of this oil with water, a white solid began forming, but then became a yellow liquid. The solvent was removed using rotary evaporation to give a white solid. After drying, the white solid was then washed with chloroform and then vacuum filtered, resulting in 1.84 g of white solid. (Yield = 93.0%)

^{31}P NMR (202 MHz, 0.1M NaOH in D_2O): δ 12.91 (t, J = 11.7 Hz, 2P)

^{1}H NMR (500 MHz, 0.1M NaOH in D_2O): δ 8.41 (d, J = 12.2 Hz, 2H, aromatic), δ 7.72 (dd, J = 10.9, 8.3 Hz, 2H, aromatic), δ 7.46 (dd, J = 8.2, 2.3 Hz, 2H, aromatic)

^{13}C NMR (500 MHz, 0.1M NaOH in D_2O): δ 140.15 (s, 2C), δ 128.38 (s, 2C), δ 122.18 (s, 4C), δ 110.22 (s, 4C) - Intensity not great enough for full characterisation.

m/z: 162.5 ($[M\text{-}2H]^{2-}$), 325.99 ($[M\text{-}H]^{-}$)

(3B) 4-Phosphono-*N*-(4-phosphonophenyl)-*N*-phenylaniline [H_4DPPA]

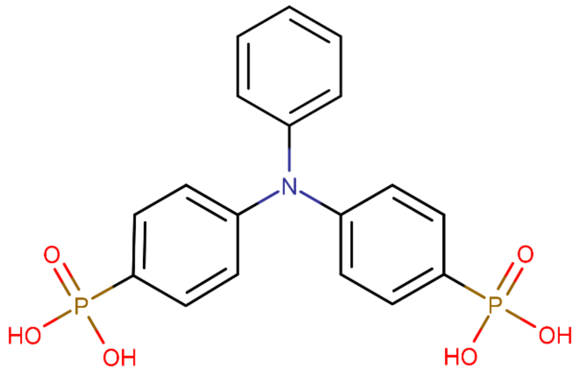

Figure S7: Chemical structure of H_4DPPA (3B).

iPr$_4$DPPA (2.5 g, 4.36 mmol) was dissolved in approximately 10 mL of acetonitrile inside a 100 mL round-bottomed flask, resulting in a clear light green solution, and was then flushed with argon. Trimethylbromosilane (2.9 mL, 31.2 mmol) was then added to the flask, resulting in a color change of the solution to a blue color. The temperature was then set to 65 °C and left to react for four hours and monitored via TLC. Once the starting material was consumed, the heating was turned off and the solution allowed to cool. Once sufficiently cooled, rotary evaporation was used to remove the solvent, acetonitrile, resulting in a blue oil. On treatment of this oil with water, a white solid began forming. Water was then progressively added until no oil remained. The white solid was then washed twice with water and acetone and dried using rotary evaporation, resulting in 1.61 g of white powder. (Yield = 91.1%)

^{31}P NMR (202 MHz, 0.1M NaOH in D_2O): δ 11.32 (t, J = 11.2 Hz, 2P)

^{1}H NMR (500 MHz, 0.1M NaOH in D_2O): δ 7.52 (dd, J = 11.2, 8.0 Hz, 4H, Aromatic), δ 7.29 (t, J = 7.7 Hz, 2H, Aromatic), δ 7.13 (d, J = 7.9 Hz, 2H), δ 7.07 (t, J = 7.5 Hz, 1H, Aromatic), δ 7.03 (d, J = 6.1 Hz, 4H)

^{13}C NMR (500 MHz, 0.1M NaOH in D_2O): δ 133.97 (d, 4C), δ 132.11 (s, 2C), - Intensity not great enough for full characterization.

m/z: 162.5 ($[M-2H]^{2-}$), 325.99 ($[M-H]^{-}$)

_N_,_N_-Bis(4-diisopropylphosphonophenyl)amine (iPr$_4$BPA) (1A)

The first synthetic target obtained was *N*,*N*-bis(4-diisopropylphosphonophenyl)amine, known herein as iPr$_4$BPA (1A). The procedure to obtain this compound was relatively simple and reproducible, whereby the product could be obtained consistently at yields above 80%. Before the workup of the crude reaction product, which took the form of a dark brown treacle-like mixture, thin layer chromatography (TLC) analysis often showed that some starting material was still present, alongside what is reasoned to be the mono-substituted intermediate, *N*-(4-diisopropylphosphonophenyl)-*N*-phenylamine. This is easily removed by employing flash chromatography, using a mixture of acetone and ethyl acetate in a 1:9 ratio. The first product to elute off the column is the starting material, followed by the mono-substituted intermediate. This leaves a pure iPr$_4$BPA (1A) product in the final vials of eluate. The pure product could then be obtained quickly by rotary evaporation to remove the solvent, resulting in a white solid in yields most often between 80 to 90%. The phase purity of this was then confirmed by ^{31}P, ^{1}H, ^{13}C, 2D HSQC NMR spectroscopy, and mass spectrometry. The solvent used for NMR was $CDCl_3$.

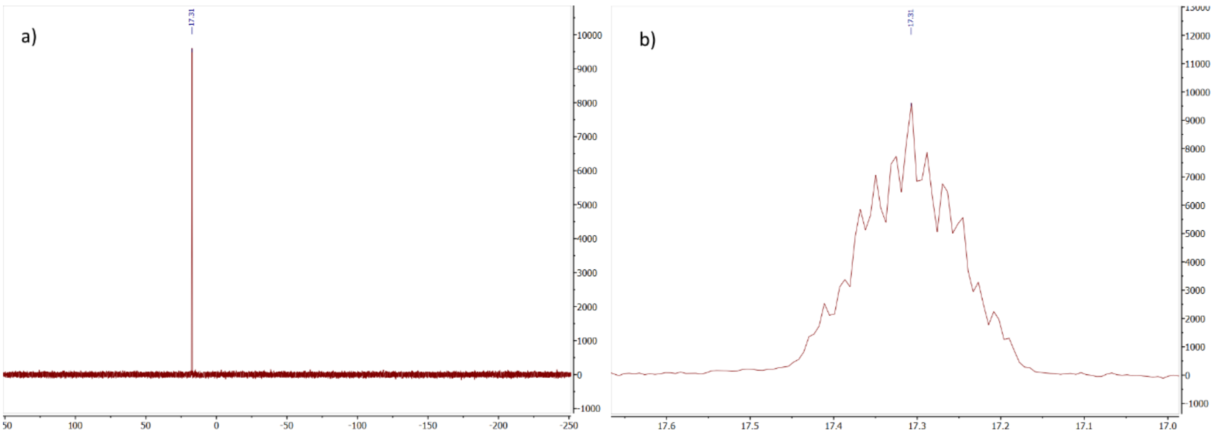

Figure S8: a) Full ^{31}P NMR spectrum, and b) zoomed ^{31}P NMR spectrum for iPr$_4$BPA (1A).

Looking at the full ^{31}P spectrum in Figure S8a we see a single signal at δ 17.3 ppm which corresponds to the phosphonate ester product, suggesting that no other phosphorus-containing impurities are present. A closer look at this signal, as shown in Figure S8b reveals a complex multiplet splitting pattern with coupling constants of between 4.4 and 50 Hz. Coupling constants at the lower end of this range indicate that three- and four-bond couplings are present, suggesting that phosphorus is coupling with the protons present on the methyl groups on the isopropyl moieties as well as the aromatic protons furthest away from phosphorus on the corresponding rings and those of the methine groups. At the upper end of this range, it is expected that phosphorus is coupling with the protons closest to it on the aromatic ring.

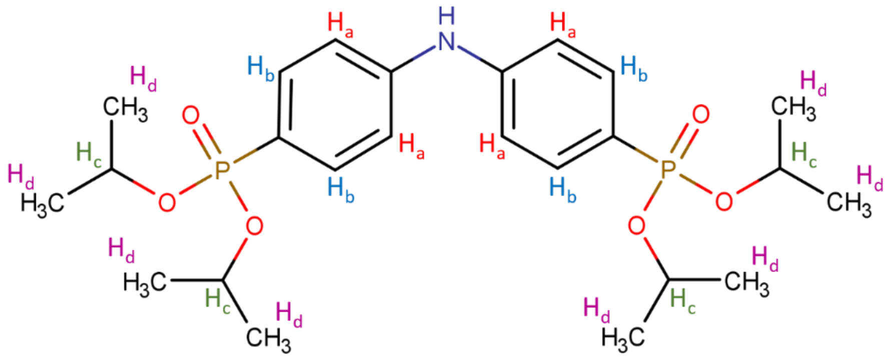

Figure S9: Chemical structure of iPr$_4$BPA with proton environments labelled H_a–H_d.

With the positive result demonstrated on the ^{31}P spectrum, ^{1}H NMR provides a similar story. A quick investigation of the full spectrum, as shown in Figure S10, immediately reveals the correct number of proton environments for the molecule, there are no significant impurities, and we see that each of the signals integrate to give the correct number of protons in each environment.

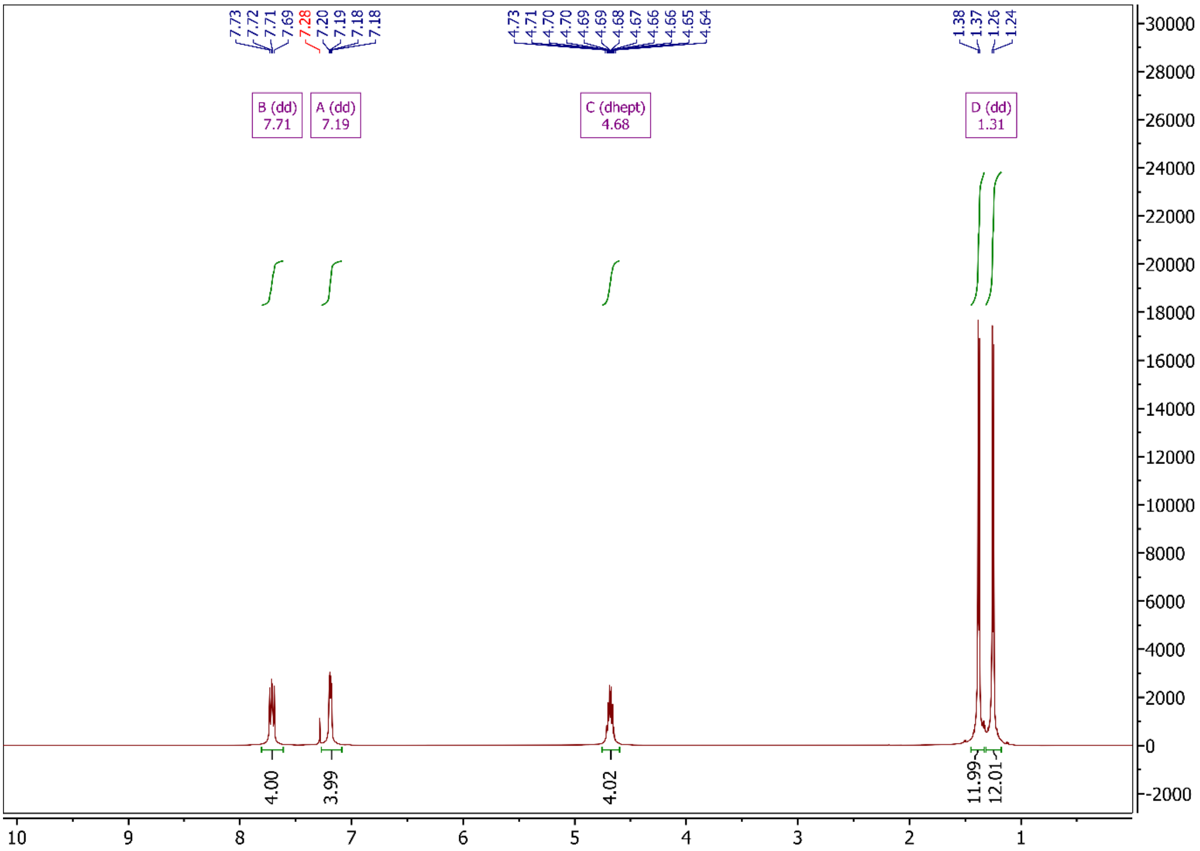

Figure S10: Full ^{1}H NMR spectrum for iPr$_4$BPA **(1A)**.

Looking specifically at the aromatic region (Figure S11) we see three peaks, including a singlet at δ 7.28 ppm, which is due to the deuterated solvent (chloroform-*d*) and two doublets of doublets at δ 7.71 ppm and δ 7.19 ppm, which correspond to the protons on the phenyl rings. On first viewing of these signals, it was tempting to assign the more downfield of the two (δ 7.71 ppm) to the protons nearest to nitrogen, represented as H_a in Figure S9, though examination of the coupling constants reveals that the downfield signal has a larger coupling constant. This implies that the signal actually represents the protons closest to the phosphonate group (H_b), since phosphorus, like a proton, has a quantum spin number of 1/2, and will thus be involved in coupling with protons. This means that the upfield signal at δ 7.19 ppm can be assigned to the protons nearest to nitrogen (H_b).

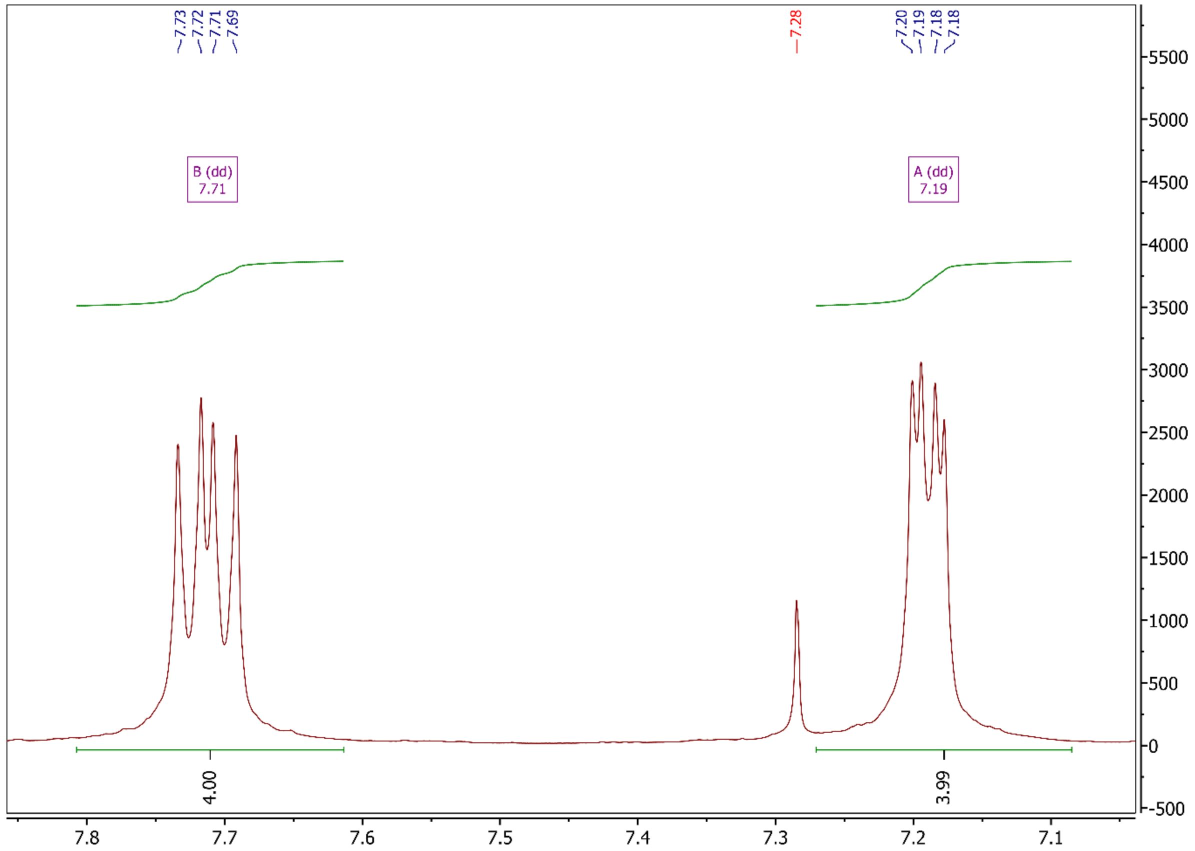

Figure S11: Aromatic region of the ^{1}H NMR spectrum for iPr$_4$BPA.

Moving further upfield (Figure S12), there is a signal at δ 4.68 ppm which is easily attributed to the methine group (H$_c$) of the isopropyl moiety. One might expect to see a septuplet here arising from the six aliphatic protons on each isopropyl moiety, though it appears to be a sextet. Closer analysis would appear to suggest that, in fact, the signal is a pair of overlapping sextets, since there are clear shoulders on some of the peaks. In this sense, however, multiplet analysis appears to miss two shoulders that would in fact make this a doublet of "leaning" septets. This analysis appears to be confirmed in the ^{1}H NMR spectrum for iPr$_4$DPC (**2A**), as seen in Figure S32, whereby a multiplet analysis appears to identify the previously hidden shoulders. The complicated nature of this signal is likely brought about by the coupling of ^{31}P and ^{1}H nuclei, as was mentioned in the discussion for the aromatic signals. Most importantly,

the signal integrates to give the correct number of protons for the methine groups on the molecule.

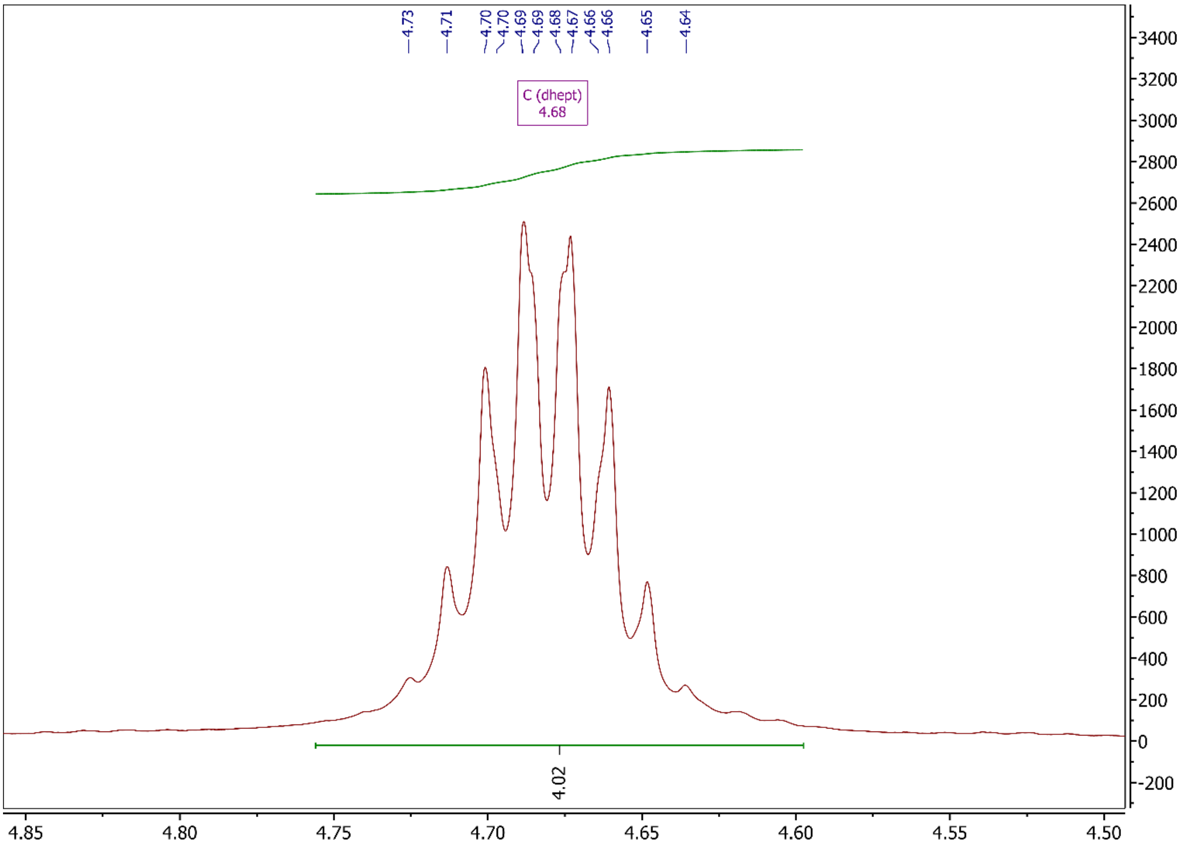

Figure S12: Methine region of the ^{1}H NMR spectrum for iPr4BPA.

The final signals on this spectrum, as seen in Figure S13, were originally thought to be a well-resolved doublet of doublets that corresponds to the terminal methyl groups (H_d) on the phosphonate ester. However, due to the relatively large coupling constant and the need to integrate the peaks separately, it was determined that the signals are more likely two doublets, indicating that there are two types of chemically inequivalent isopropyl groups present on the molecule, though how these arise has not been investigated. Working on this basis, the signal integrates to a total of 24 protons, as expected. Each of the doublets arises from the coupling with the methine proton.

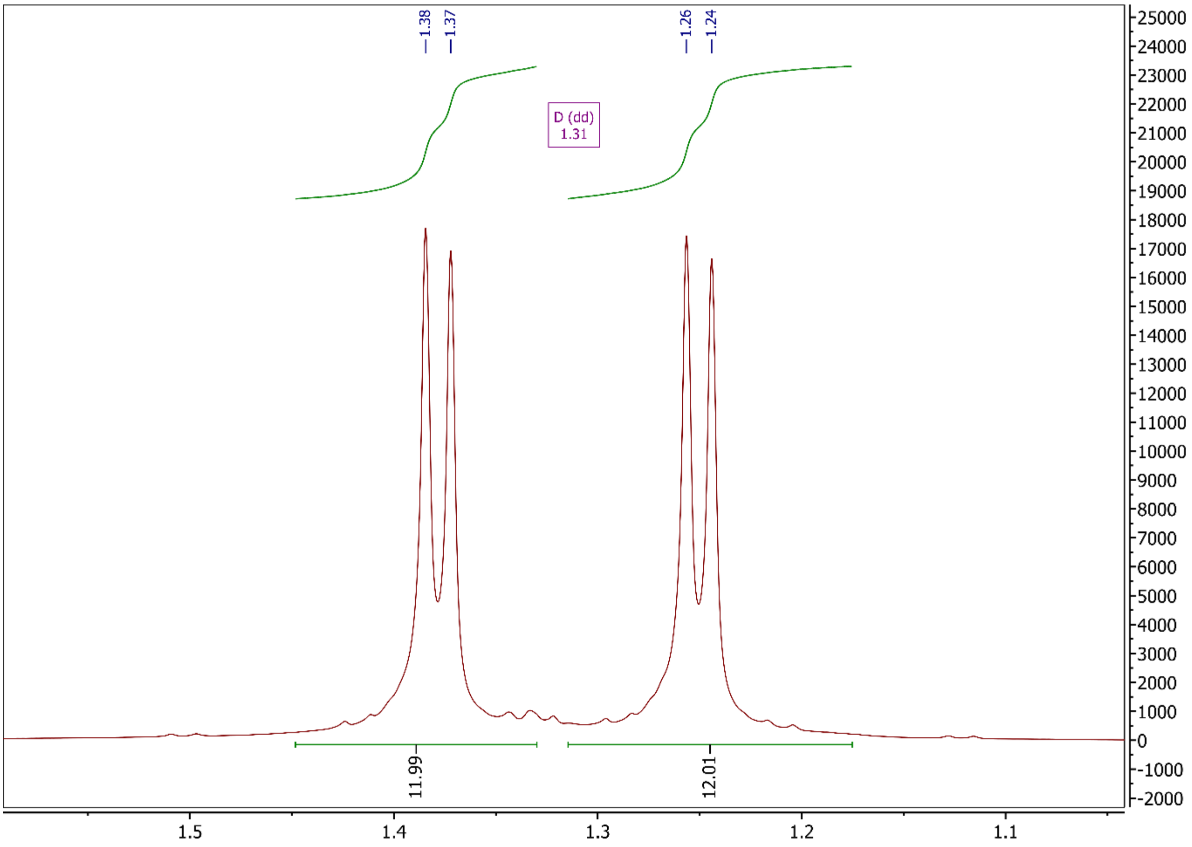

Figure S13: Methyl region of the ^{1}H NMR spectrum for iPr$_4$BPA.

Moving to the ^{13}C NMR spectrum, as shown in Figure S15, we can immediately see the number of signals expected. Starting downfield, the signal at δ 145.13 ppm is the result of the carbons bonded to nitrogen, labelled A. Moving upfield, we see the peak at δ 133.50 ppm corresponding to the four carbons labelled C. Next is the peak at δ 122.65 ppm, which corresponds to the two carbons bonded to phosphorus, labelled D. The final peak for the aromatic region is the one labelled B at δ 116.97 ppm, which corresponds to four carbons in the β-positions relative to nitrogen. We then see a large peak at δ 76.0 ppm which goes off the scale and belongs to the solvent, $CDCl_3$. Next to this, at δ 70.55 ppm, is the peak attributed to methine carbon on the isopropyl groups. The final peak, at δ 24.00 ppm, corresponds to the terminal methyl carbons on the isopropyl groups.

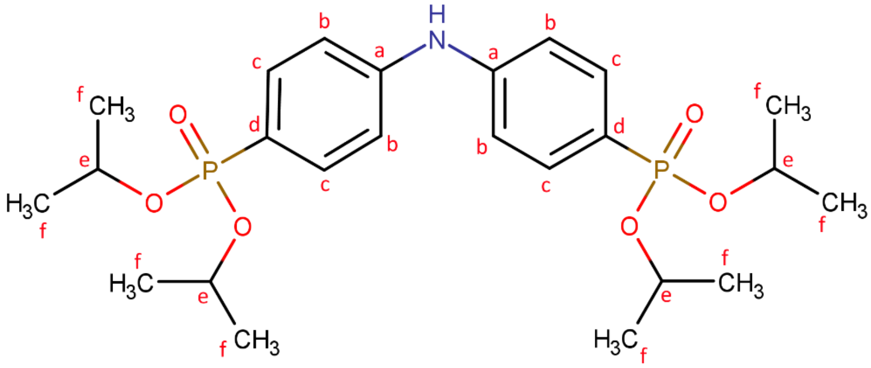

Figure S14: Chemical structure of iPr$_4$BPA with carbon environments labelled a–f.

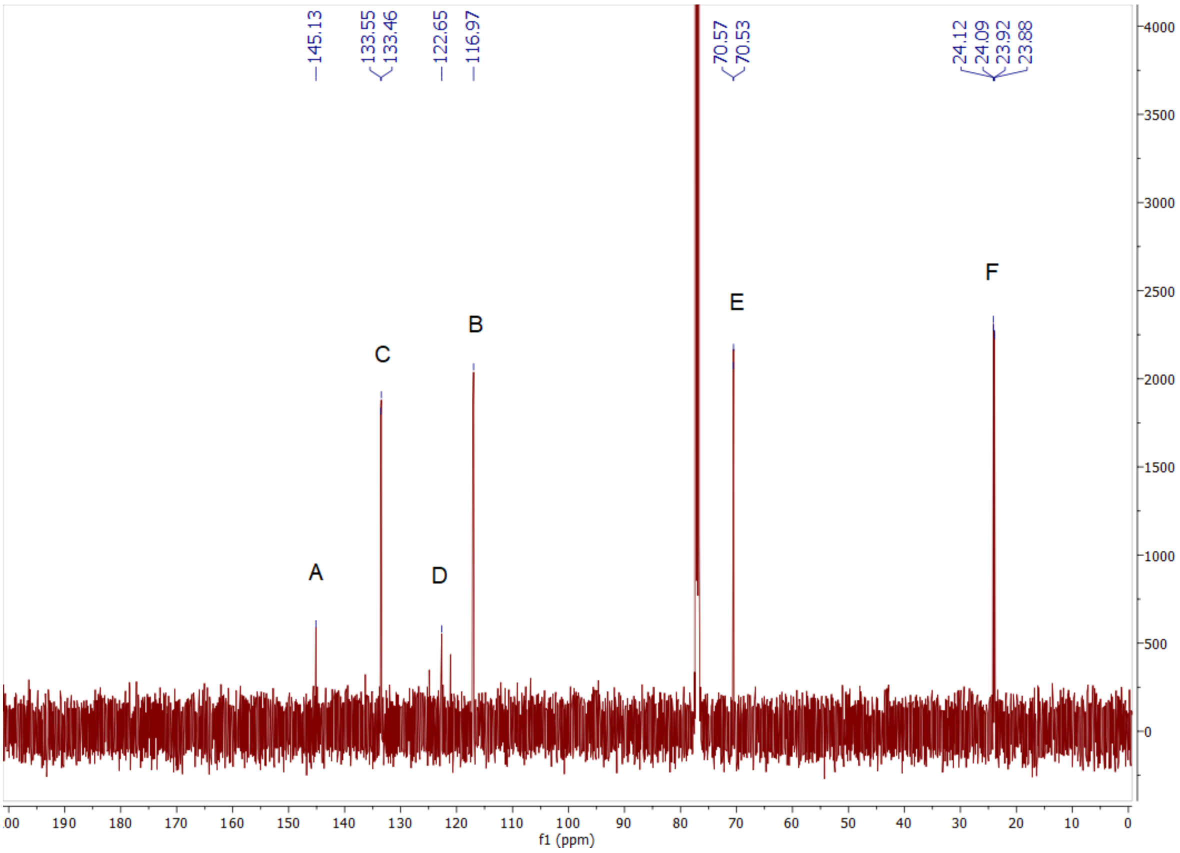

Figure S15: Full ^{13}C NMR spectrum for iPr$_4$BPA.

In order to obtain further confirmation of the NMR results, 2D H-C coupled NMR (HSQC) was carried out, which at least allows the assignment of carbon signals based on those for the proton signals. Looking at Figure S16, we can immediately see that the furthest downfield signals on both spectra, at δ 133.50 ppm, correlate to B and C

on the proton and carbon NMR spectra, respectively. The other correlation in the aromatic region, at δ 116.97 ppm, is shown between B on the proton NMR spectrum and C on the carbon NMR spectrum. The next two signals, at δ 70.55 ppm and δ 24.00 ppm correctly show the H-C correlations for the methine and methyl groups.

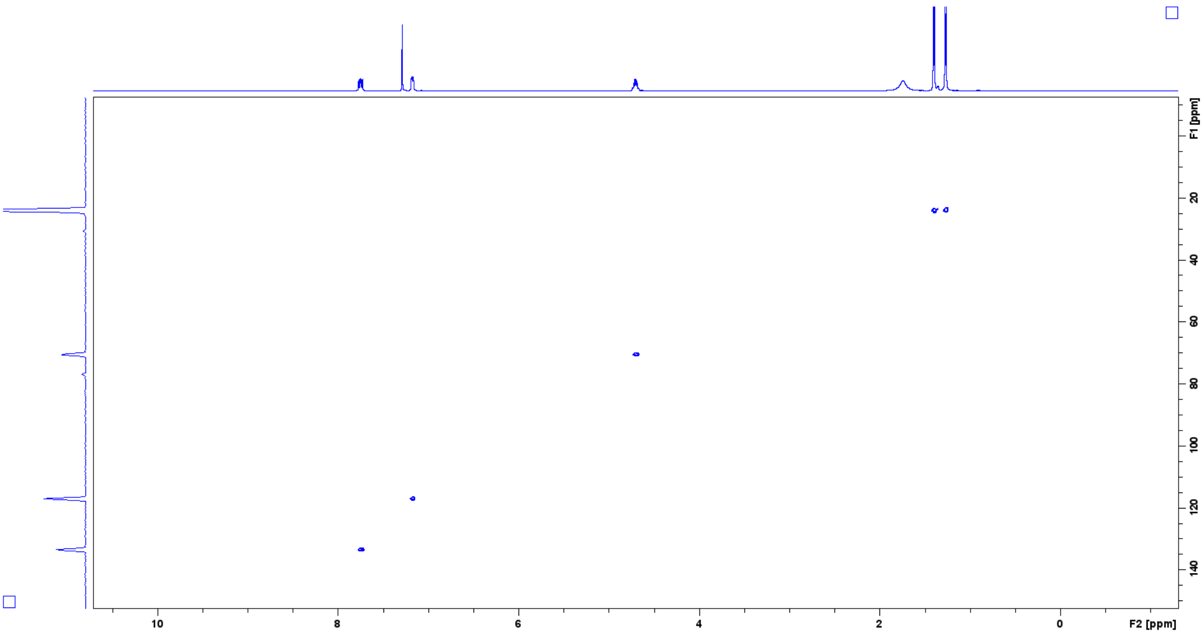

Figure S16: Full HSQC NMR spectrum for iPr_4BPA.

With NMR data clearly showing successful synthesis of iPr_4BPA, mass spectral analysis was carried out to provide further support. Figure S17 shows the full mass spectrum, where the base peak can be seen with an *m*/*z* of 498.22 and is shown in more detail in Figure S18. Here we see the theoretical isotope profile for the $[M + H]^+$ species and the observed experimental data, with an almost exact match between the two. Moving back to the full spectrum, the peak at *m*/*z* 995.43 corresponds a dimer species, $[2M + H]^+$, of the target molecule. The peak at *m*/*z* 330 has a clear relation to those at *m*/*z* 755.33 and 1509.66, whereby the former is likely a degradation product of the target molecule, likely corresponding to the acid form, $[N(C_6H_4)_2 + H]^+$, and the latter two corresponding to the dimer and trimer species, respectively.

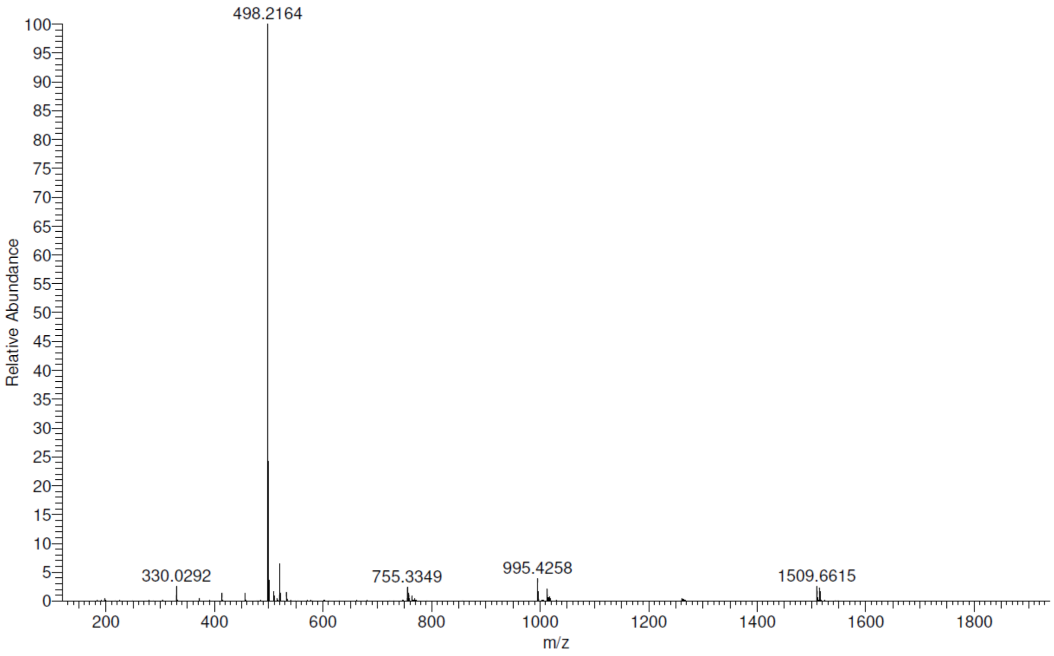

Figure S17: Full mass spectrum for iPr4BPA.

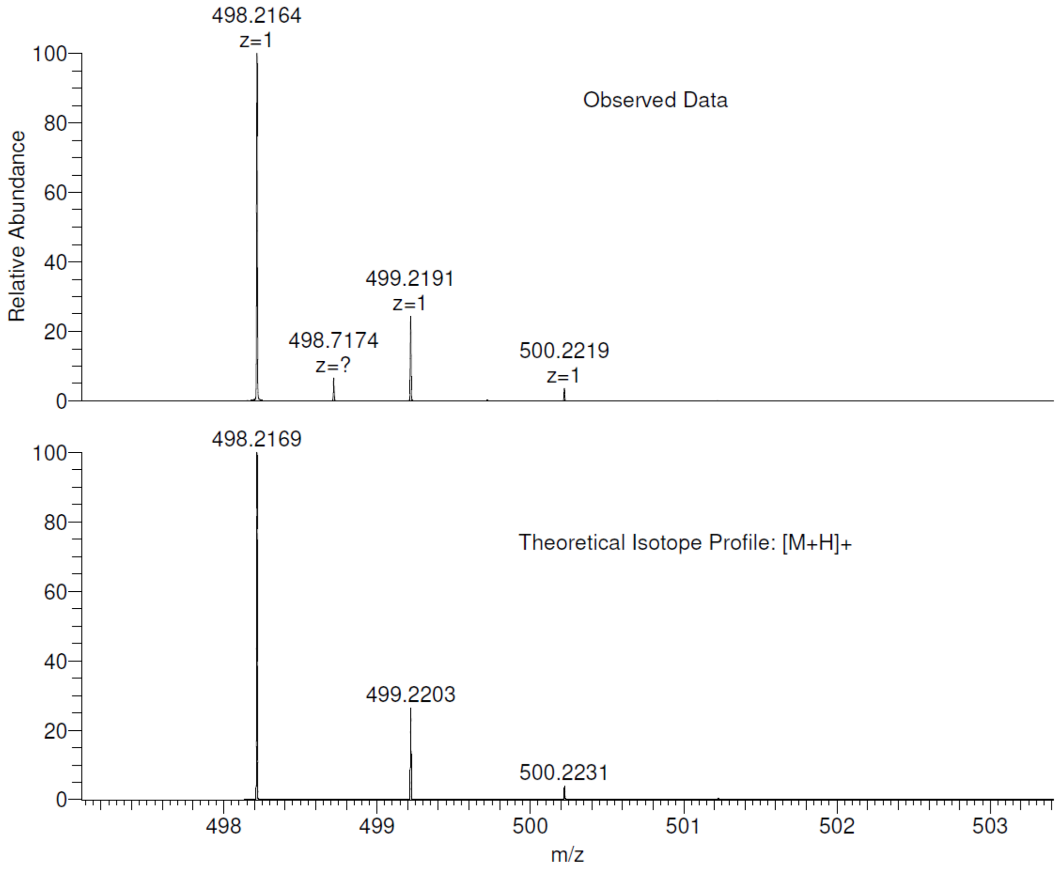

Figure S18: Mass spectrum for iPr4BPA showing the observed data and the theoretical isotope profile for the $[M + H]^+$ species.

_N,N_-Bis(4-phosphonophenyl)amine (H_4BPA) (1B)

With a clean product obtained for iPr$_4$BPA (**1A**), the attention shifted to obtaining the corresponding phosphonic acid, *N*,*N*-bis(4-phosphonophenyl)amine H_4BPA (**1B**). The initial method considered for this was hydrolysis using 6 M hydrochloric acid under reflux for 12+ h. The results here were quite hit and miss, as it was found that this route sometimes led to the cleavage of the C–P bond, which was confirmed by the absence of signals on the ^{31}P NMR spectrum. As an alternative to these harsh conditions, a different route which involved silylation using trimethylsilyl bromide (TMSiBr) and subsequent hydrolysis using water was chosen. Overall, this method proved very simple and required very little work up, often just requiring separate washes with water and acetone, and leading to yields between 65 to 91%. As with the ester, purity is confirmed with both ^{31}P, ^{1}H, ^{13}C, and HSQC NMR spectroscopy, and mass spectrometry. The solvent used for NMR was a 0.1 M solution of NaOH in D_2O. Looking first at the ^{31}P spectrum (Figure S19), it is clear that the splitting pattern has completely changed this time taking the form of a singlet, which suggests that successful hydrolysis and removal of the isopropyl groups has occurred. The peaks are less resolved in this case, so no splitting patterns are observed despite the peak width remaining similar to that of the ester, as shown in Figure S8. It is also clear that no phosphorus-containing impurities are present, and thus the washing protocol used is satisfactory.

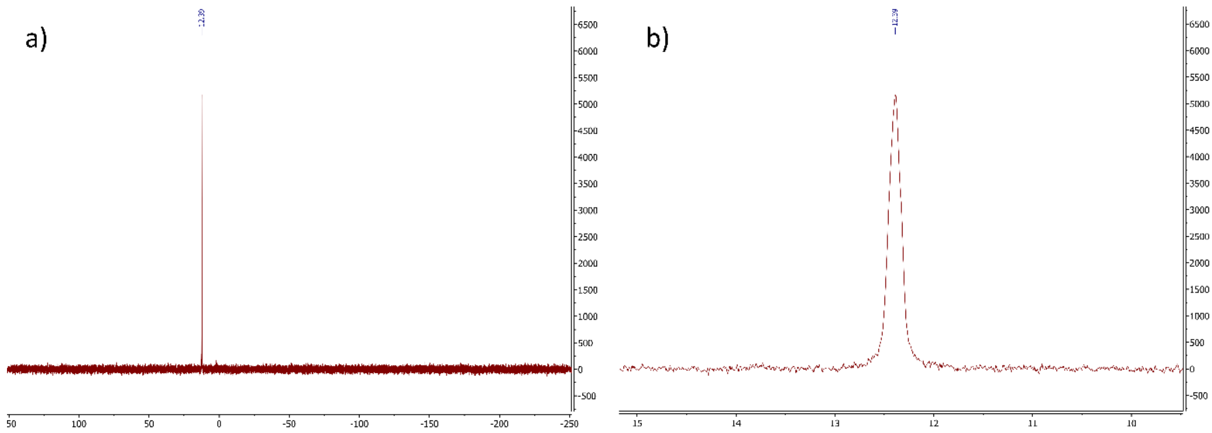

Figure S19: a) Full ^{31}P NMR spectrum, and b) zoomed ^{31}P NMR spectrum for H_4BPA (**1B**).

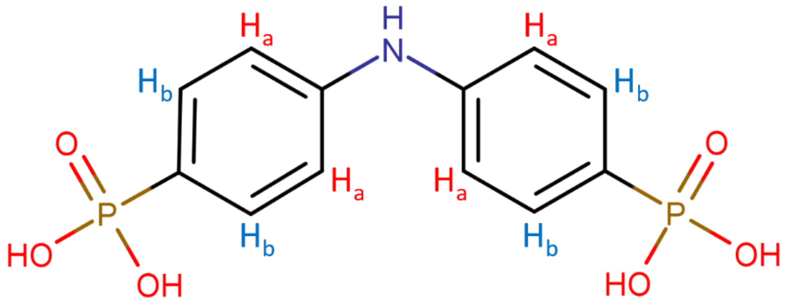

Figure S20: Chemical structure of H_4BPA with proton environments labelled H_a and H_b.

Moving next to the ^{1}H NMR spectrum, as shown in Figure S21, it is clear that the isopropyl groups are no longer present, since the signals for the methyl ($-CH_3$) and methine (-CH-) groups have disappeared, giving further confirmation that hydrolysis has successfully taken place.

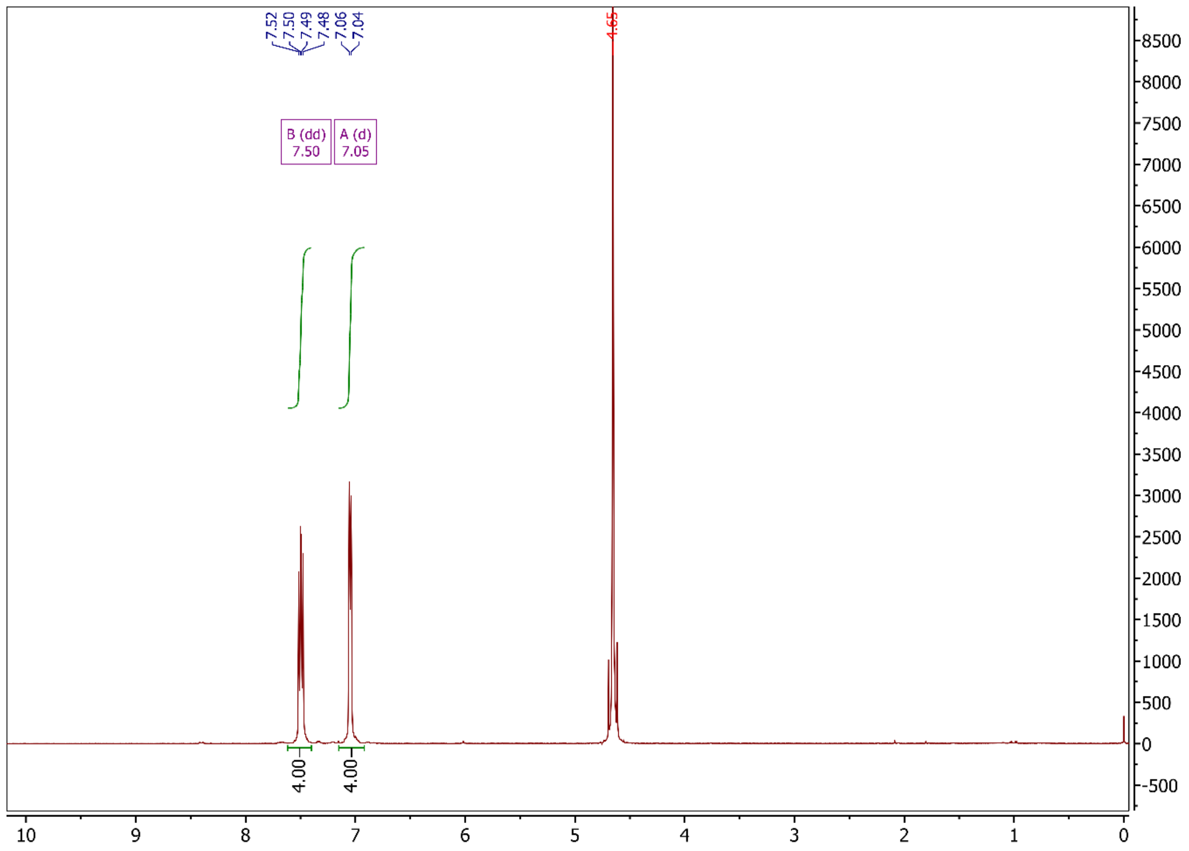

Figure S21: Full ^{1}H NMR spectrum for H_4BPA (**1B**).

Taking a closer look at the aromatic region, as shown in Figure S22, the signals can be assigned as they were for iPr$_4$BPA (**1A**), whereby the downfield signal at δ 7.50 ppm (B) can be assigned to the protons nearest to phosphorus on the phenyl ring, represented by H_b, since it has a larger coupling constant. The signal at δ 7.05 ppm (A) can be assigned to the protons nearest to nitrogen on the phenyl ring, represented by H_a. This signal, while appearing as a single doublet, is more likely to be an unresolved doublet of doublets, as was seen in Figure S11 for iPr$_4$BPA (**1A**).

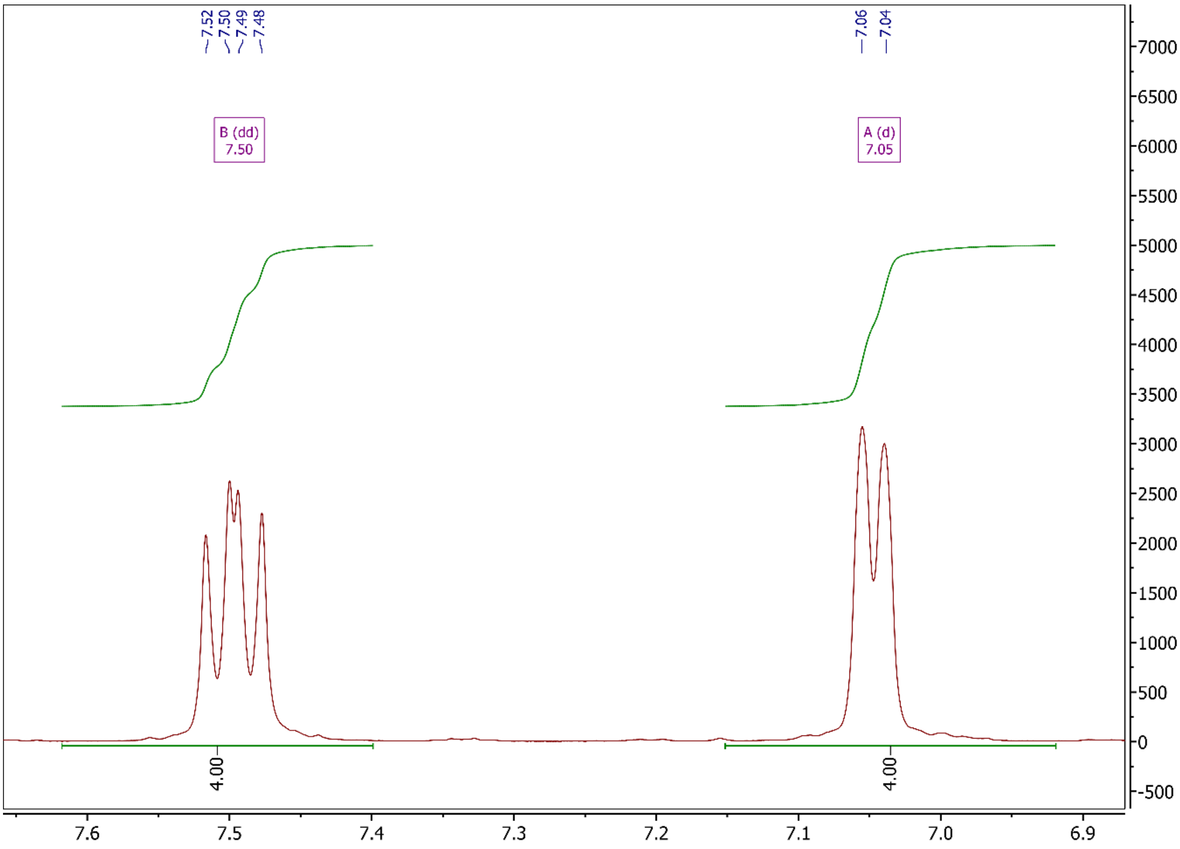

Figure S22: Aromatic region of the ^{1}H NMR spectrum for H_4BPA.

Moving to the ^{13}C NMR spectrum, as shown in Figure S24, we can immediately confirm the lack of signal for the methine and methyl groups. Starting downfield, we see the peak, labelled A on the spectrum, which has been assigned to the two carbon atoms bonded to nitrogen, as was the case for iPr_4BPA. The next signal, labelled D, has been assigned to the two carbons bonded to phosphorus, which has actually shifted when compared with the spectrum for iPr_4BPA. The next two signals then are assigned to the carbons with bonded protons, labelled C and B respectively, with the latter of these being the more downfield. It was unfortunate here, in fact, that the acid is relatively insoluble and thus obtaining a spectrum with reasonable intensities was not possible, even though the signals were able to be assigned.

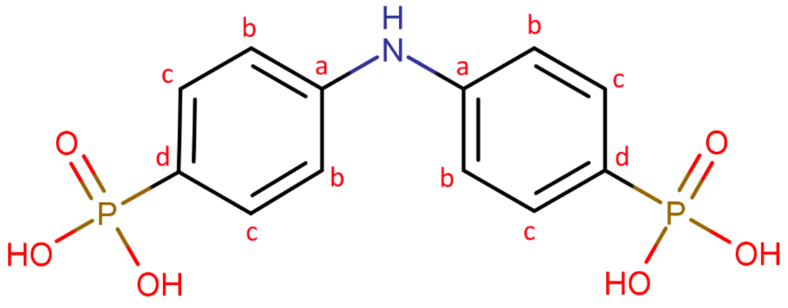

Figure S23: Chemical structure of H_4BPA with carbon environments labelled a–f.

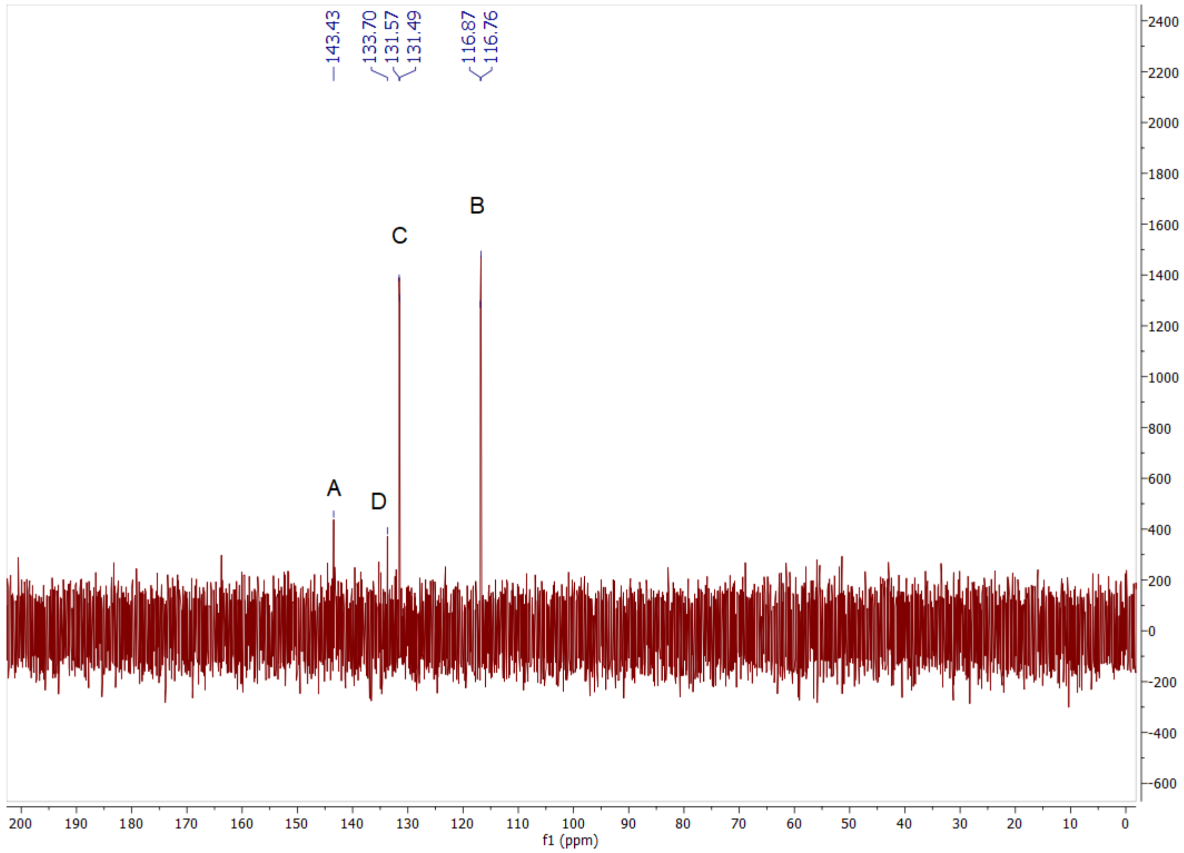

Figure S24: Full ^{13}C NMR spectrum for H_4BPA.

As was done for iPr_4BPA, we again use coupled C–H NMR (HSQC) to lend support for the assignments for H_4BPA NMR spectra. Looking at the spectra in Figure S25, we can again see the lack of signals associated with the isopropyl groups. We can,

however, see the two expected signals for the two sets of aromatic carbons with associated protons.

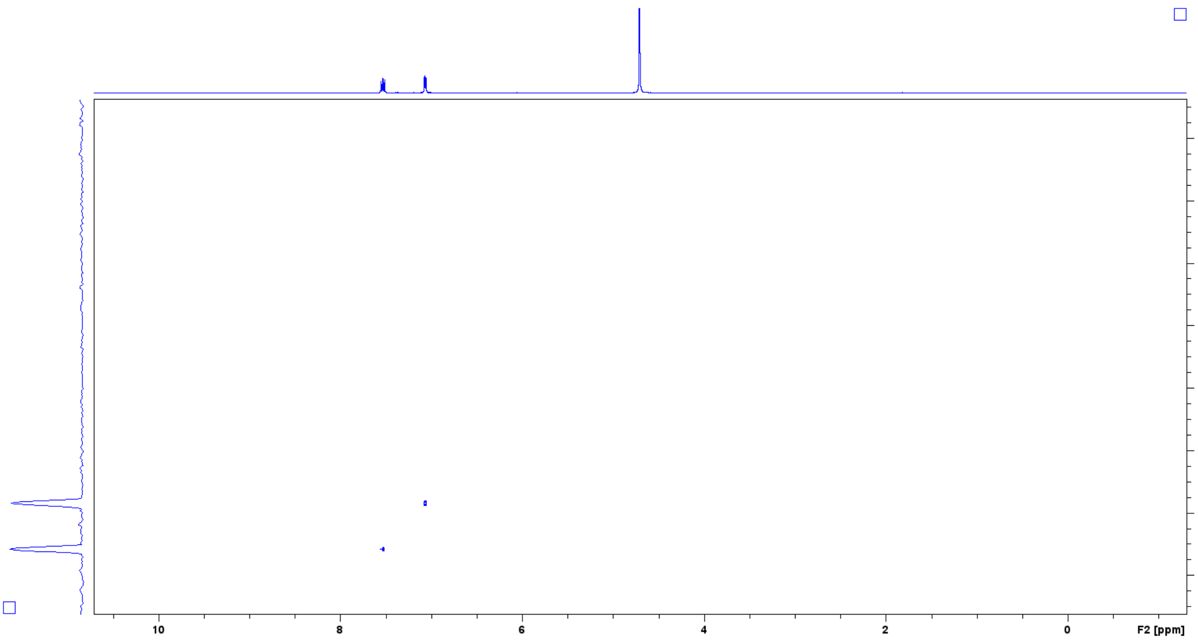

Figure S25: Full HSQC NMR spectrum for H_4BPA.

As with the ester precursor, mass spectral analysis was carried out to confirm the results of the NMR analysis. Looking at Figure S26, the base peak is clear at a *m*/*z* of 163.50, which corresponds to the $[M + 2H]^{2+}$ species. Figure S27 takes a closer look at this peak, showing a close match to the theoretical isotope profile. A second peak is located with a *m*/*z* of 328.01, and corresponds to the $[M + H]^{+}$ species.

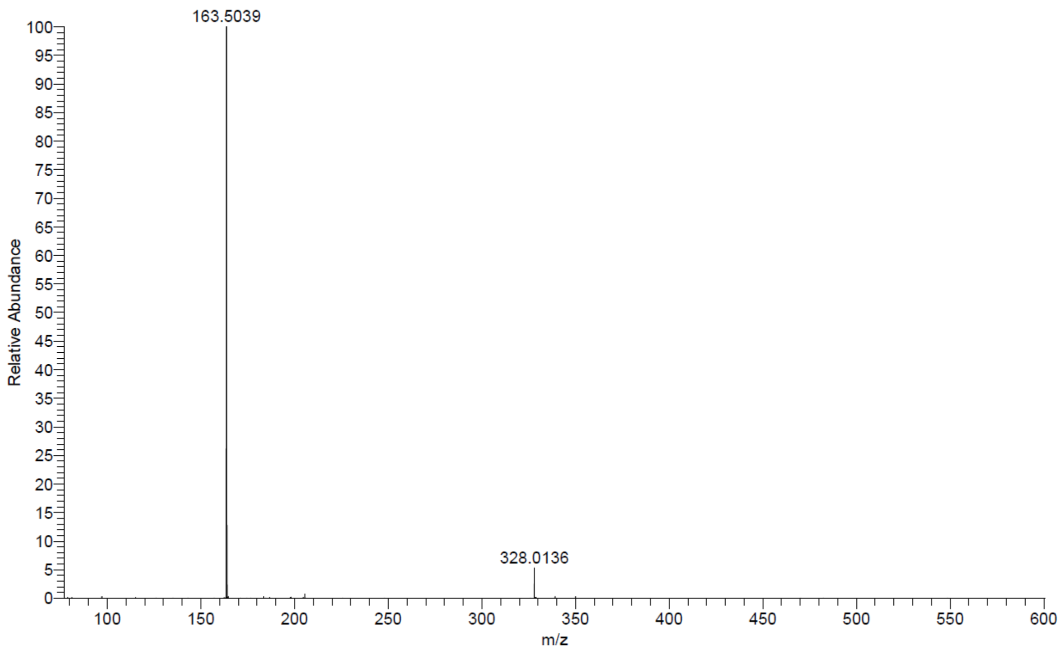

Figure S26: Full mass spectrum for H_4BPA.

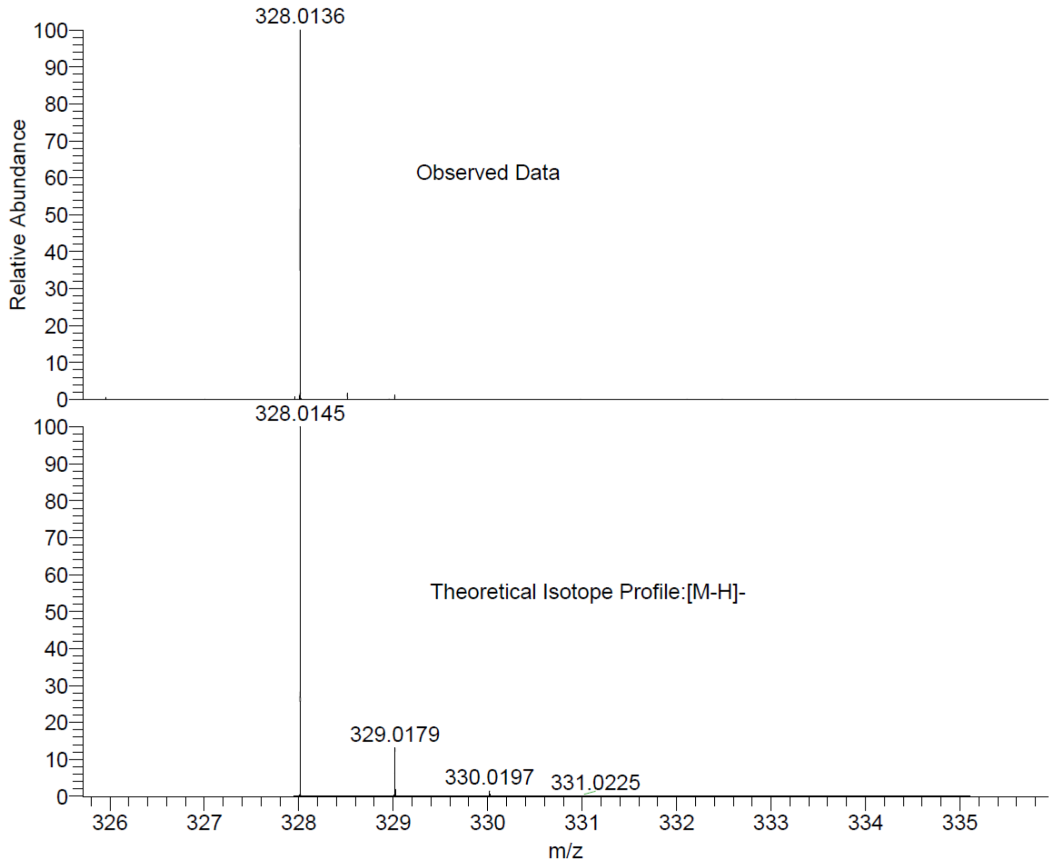

Figure S27: Mass spectrum for H_4BPA showing the observed data and the theoretical isotope profile for the $[M - H]^-$ species.

3,6-Bis(diisopropylphosphono)-9*H*-carbazole (iPr$_4$DPC) (2A)

The second synthetic target, building upon the success of the first linker, was 3,6-bis(diisopropylphosphono)-9*H*-carbazole (iPr$_4$DPC, **2A**). As with the previous linker, this was also a simple and reproducible procedure from which relatively high yields could be obtained. As with the amine linker, the crude reaction product was most often obtained as a dark brown treacle-like mixture, containing the target product alongside the monosubstituted side product, some starting material, and some triisopropyl phosphite. Washing this in hexane then yielded a pinky/off-white solid, which could then be purified by flash chromatography. The yields here were often higher on average than for the previous linker, with synthetic yields obtained in the range of 82–97%. The solvent used for NMR was $CDCl_3$.

The ^{31}P NMR spectrum, as shown in Figure S28, again reveals a significant signal corresponding to the target product, which shows a less resolved splitting pattern than was observed for iPr$_4$BPA (**2A**), though it still indicates higher order coupling with both the methine (H_d) and methyl (H_e) groups on the isopropyl moiety, as well as the aryl protons $H_{a–c}$.

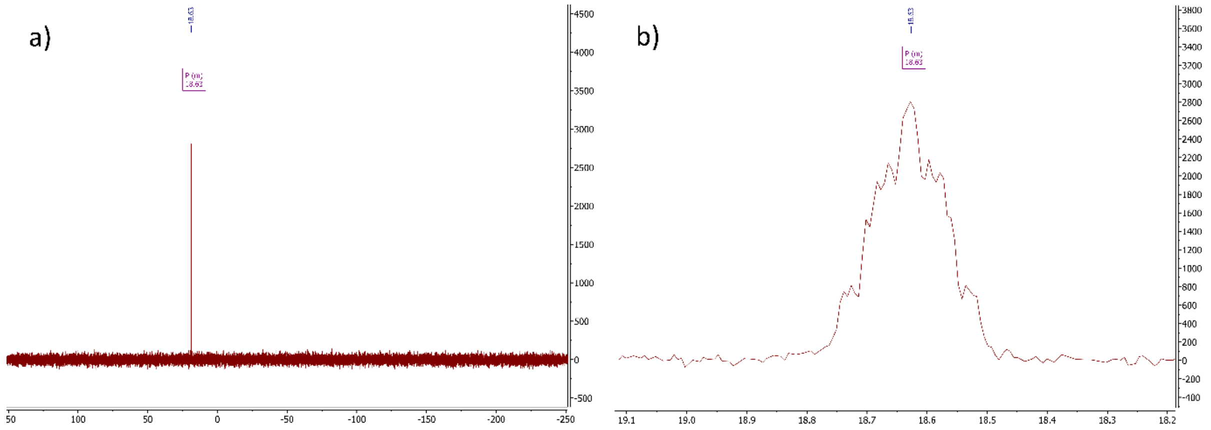

Figure S28: a) Full ^{31}P NMR spectrum, and b) zoomed ^{31}P NMR spectrum for iPr$_4$DPC (**2A**).

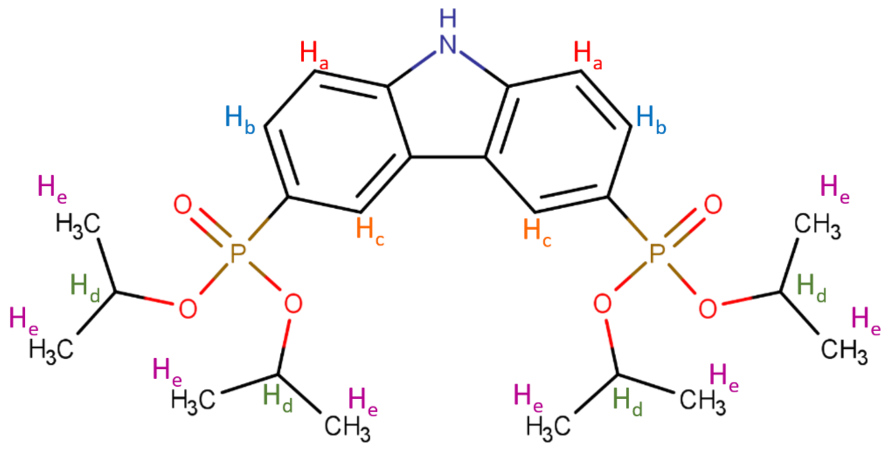

Figure S29: Chemical structure for iPr_4DPC with proton environments labelled H_a–H_e.

Moving to the ^{1}H NMR spectrum, in Figure S30 we see that there are some unexpected signals amongst those attributed to the product. Firstly, at δ 1.76 ppm we see a slightly broadened singlet, which is likely to be water, though the chemical shift is slightly higher than you would usually expect for water, δ 1.56 ppm. It is also possible that this belongs to the methyl group of either triisopropyl phosphite [$P(OiPr)_3$] and/or isopropyl bromide [$BrCH(CH_3)_2$]. This is also the case for the peaks at δ 4.64 ppm and δ 1.35 ppm. Despite the difficulty in identifying this component, it should be noted that this did not seem to have an effect on the synthesis of the corresponding phosphonic acid and is therefore not too much of a concern.

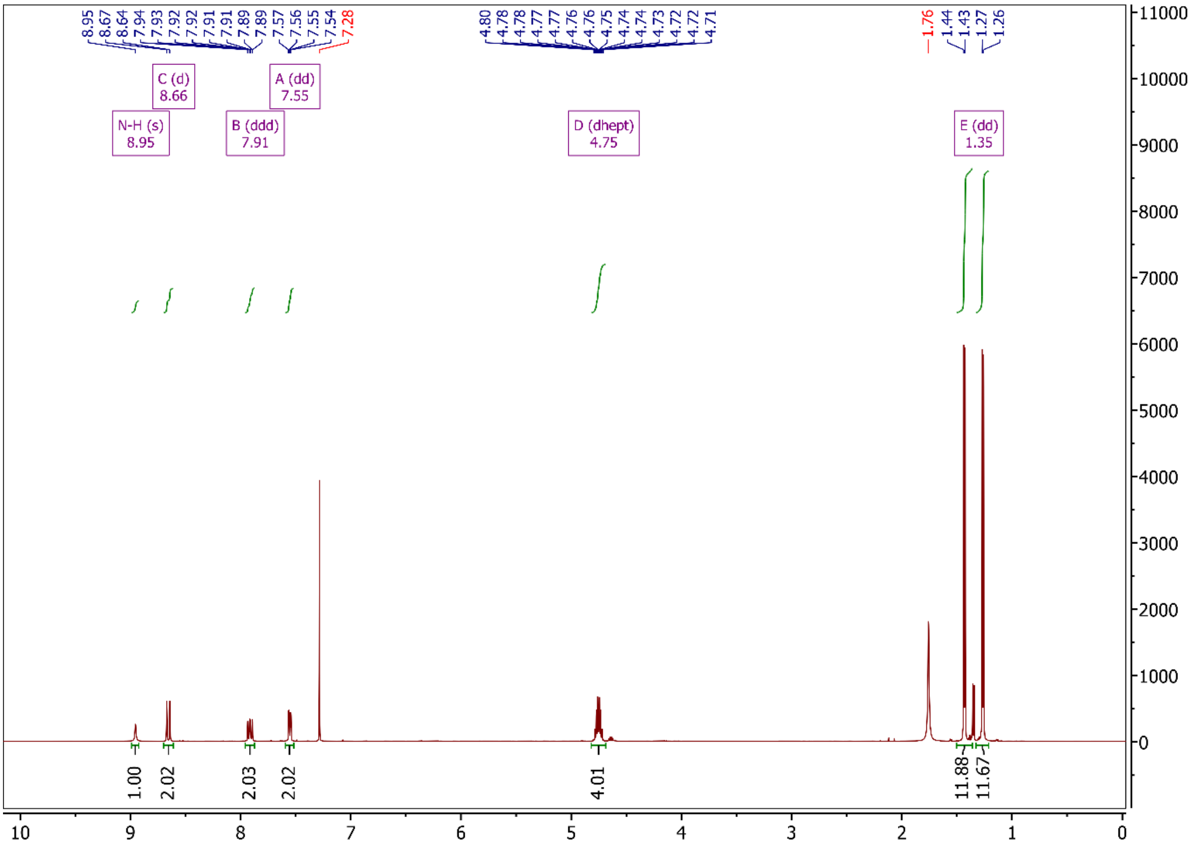

Figure S30: Full ^{1}H NMR spectrum for iPr$_4$DPC (2A).

Moving to Figure S31 to focus on the aromatic region, we see four signals, three of which are to be attributed to the aryl protons and one to the proton bound to nitrogen. The latter of these has been attributed to the broadened signal at δ 8.95 ppm and integrates to 1 proton. Though you might not usually expect to see a signal for these protons, the solvent used, chloroform-*d*, is both apolar and aprotic, so there is no considerable exchange with the N–H proton. What we are left with then are the three signals associated with the aryl protons. The first of these signals (C), a doublet at δ 8.66 ppm, has been attributed to the proton labelled H_c in Figure S29. The splitting here is likely due to higher order coupling with phosphorus, causing the signal to display higher coupling constant than the other signals, which also display splitting patterns that indicate neighbouring protons, which H_c does not have. The next signal

(B), at δ 7.91 ppm, has been attributed to the aryl proton labelled H_b in Figure S29. Here we see a doublet of doublets of doublets (ddd), which is the result of the multiple opportunities for coupling. You would initially expect to see just a doublet caused by coupling with the aryl proton H_a, but this splits further due to coupling with both the aryl proton H_c and phosphorus, resulting in the ddd splitting pattern observed. This leaves the final signal (A) in this region, a doublet of doublets (dd) at δ 7.55 ppm, which has been attributed the proton labelled H_a. Coupling the adjacent aryl proton causes the initial splitting into a doublet, while further coupling with phosphorus causes further splitting, resulting in the observed doublet of doublets. This is again supported by the smaller coupling constants observed as you move to smaller δ shifts.

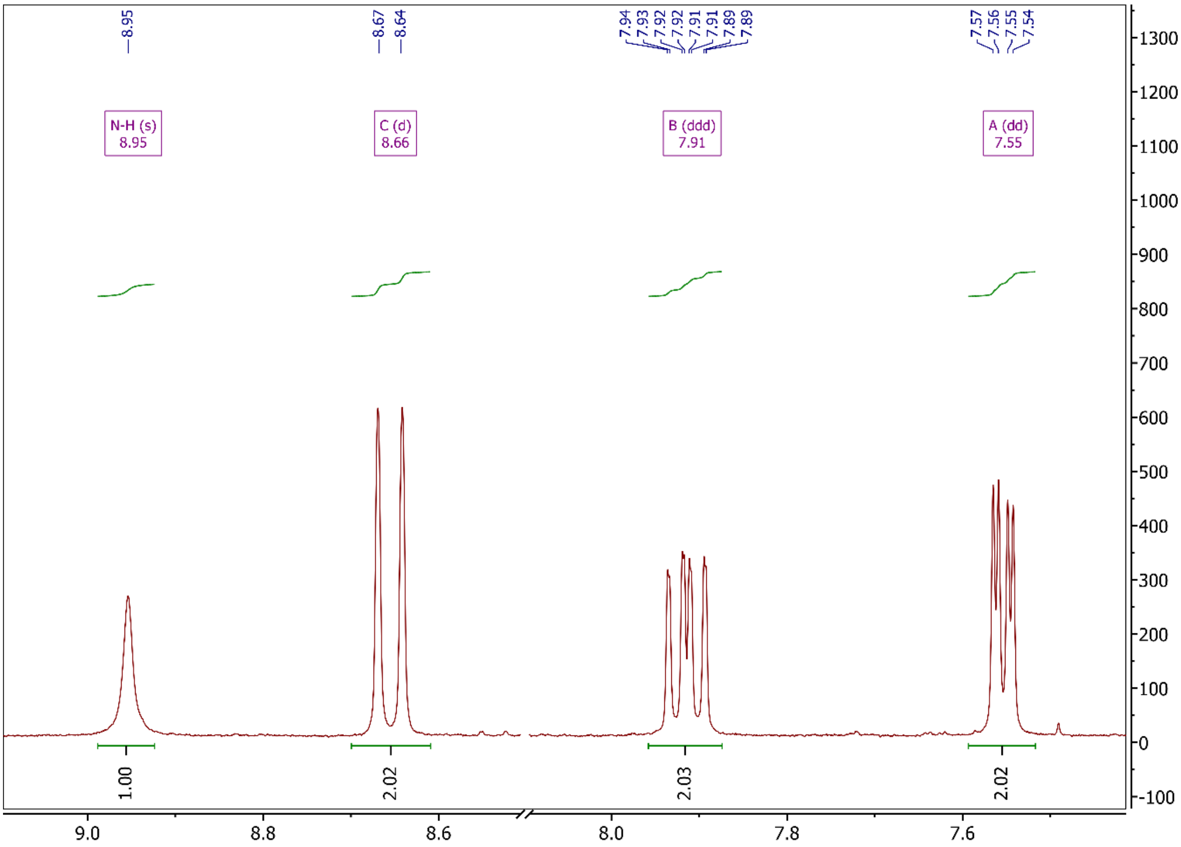

Figure S31: Aromatic region of ^{1}H NMR spectrum for iPr$_4$DPC (**2A**).

As was previously discussed for the methine signal for iPr4BPA, the ^{1}H NMR spectrum for iPr4DPC, as shown in Figure S32, also shows a signal for the methine protons, though at a slightly higher shift of δ 4.75 ppm. In the case of iPr4BPA, it was mentioned that the signal is likely a pair of unresolved septets, which is something that is much clearer here, showing that the original designation as a pair of leaning septets to be quite likely. Most importantly, the signal integrates to the correct number of protons. There is also a similar, though much less intense, signal at ≈ δ 4.64 ppm which can be attributed to the methine group of leftover triisopropyl phosphite, which is not unexpected considering the signal at δ 1.35 ppm, which is attributed to the methyl group of triisopropyl phosphite.

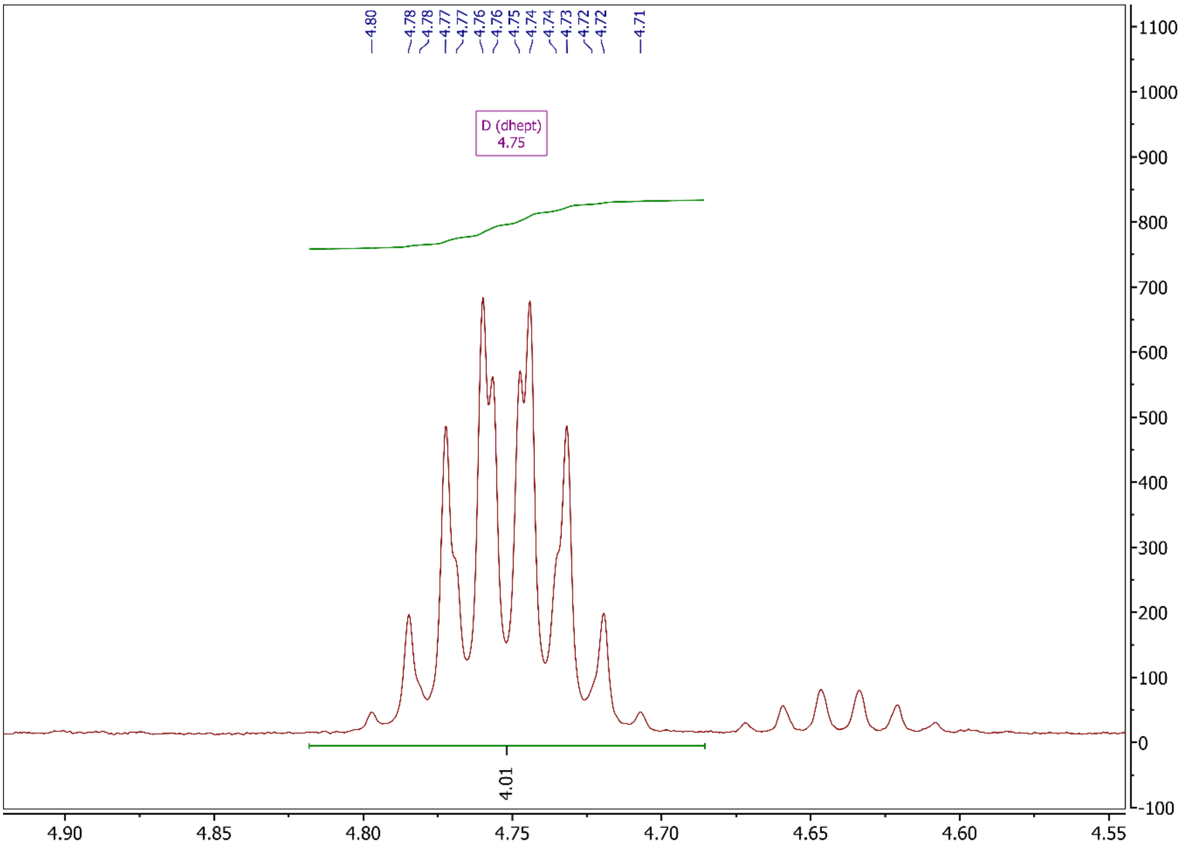

Figure S32: Methine region of ^{1}H NMR spectrum for iPr4DPC (**2A**).

The final signals of concern in this spectrum, which again, was initially thought to be a doublet of doublets, can be seen at δ 1.435 and 1.265 ppm in Figure S33. As was established for iPr$_4$BPA, however, this is in fact more likely to be two doublets which are the result of two chemically distinct methyl groups, and the splitting in each of the doublets arises through coupling with the methine proton. There is also a small impurity present between the methyl peaks at δ 1.35 ppm, which, as mentioned previously, is suspected either isopropyl bromide or triisopropyl phosphite. If quantified using the integration based on the assumption that phosphite is present, this would approximate to a 1:9 ratio of phosphite to iPr$_4$DPC.

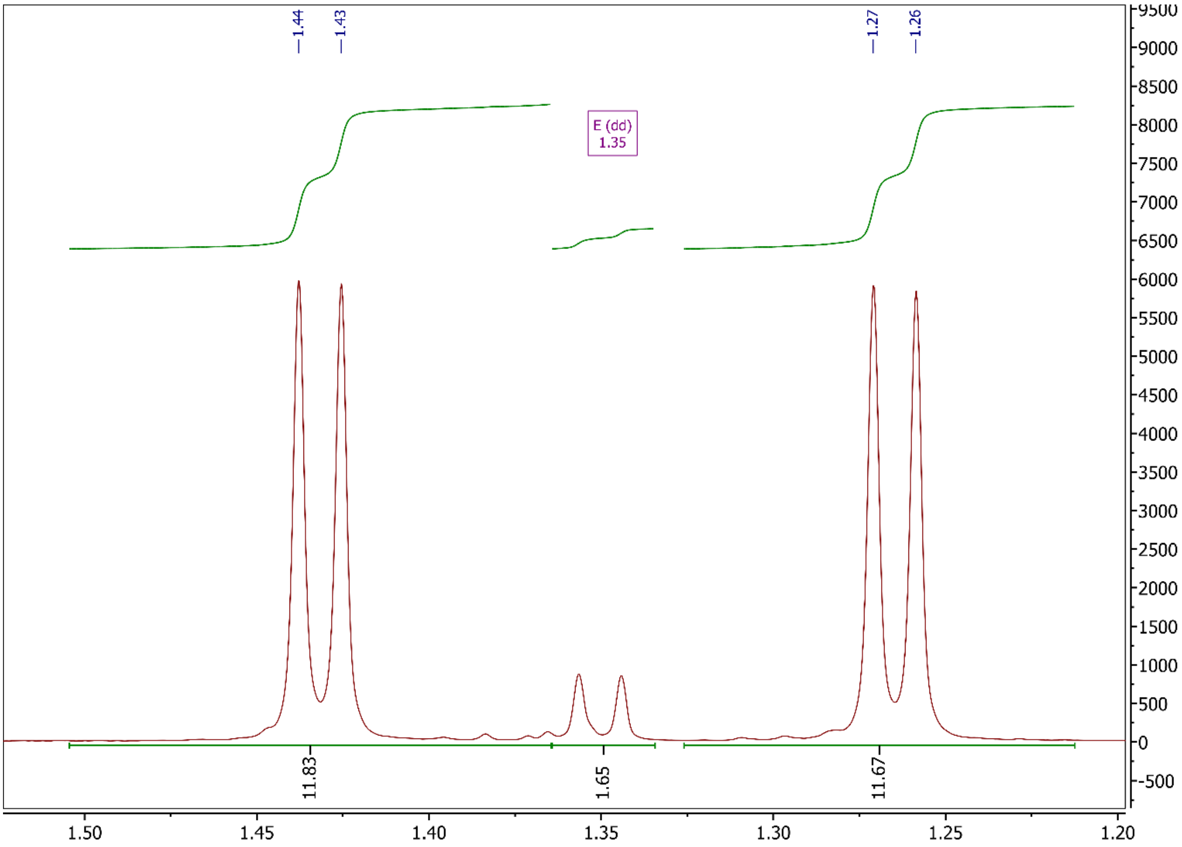

Figure S33: Methyl region of ^{1}H NMR spectrum for iPr$_4$DPC (**2A**).

Looking to the ^{13}C NMR spectrum shown in Figure S35 for iPr$_4$DPC, we can immediately see it is slightly more complicated than that of iPr$_4$BPA. Starting

downfield, the first signal we see is the one at δ 141.98 ppm, which has been assigned to the carbons bonded to nitrogen, labelled A. Next we see two signals, labelled C (δ 129.69 ppm) and E (δ 125.40 ppm), have been assigned to the aromatic carbons bearing protons, labelled accordingly, with the third of these aromatic signals (δ 110.96 ppm) labelled B. Then we have the peak labelled D at δ 122.84 ppm, assigned to the carbon bonded to phosphorus, followed by the peak at δ 121.40 ppm which is assigned to carbon in the β-position, relative to nitrogen on the pyrrole-like ring. The final two peaks of interest labelled G (δ 70.66 ppm) and H (δ 24.04 ppm) are assigned to the carbons on the isopropyl groups.

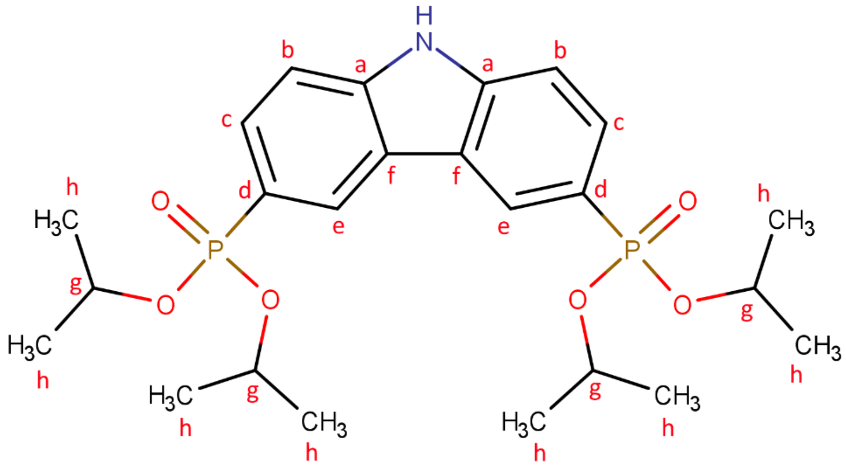

Figure S34: Chemical structure of H_4BPA with carbon environments labelled a–f.

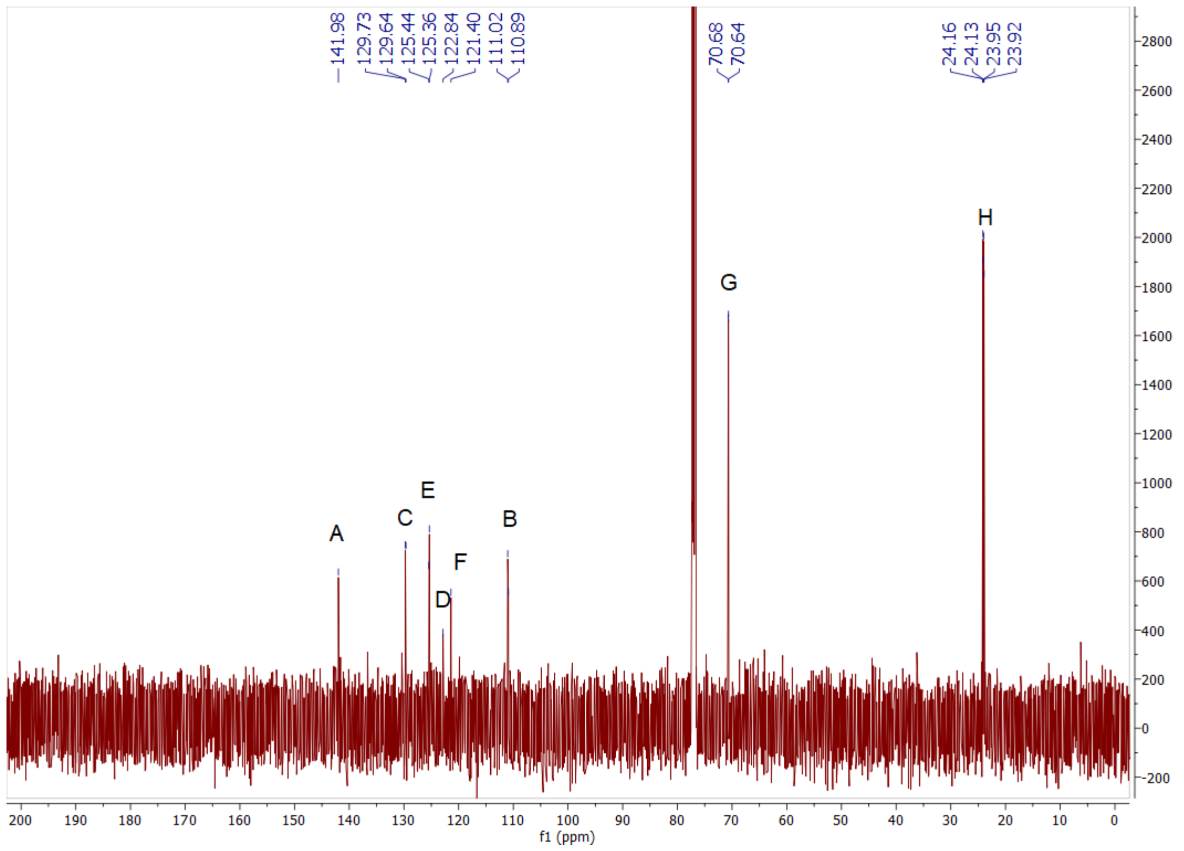

Figure S35: Full ^{13}C NMR spectrum for iPr$_4$DPC (**2A**).

Looking to the HSQC NMR spectra for clarification, as shown in Figure S36, we can confirm that the peaks labelled C, E, and B on the ^{13}C NMR spectrum, are indeed correlated with those labelled B, C, and A on the ^{1}H NMR spectrum. We also see the expected correlations for the methine and methyl groups.

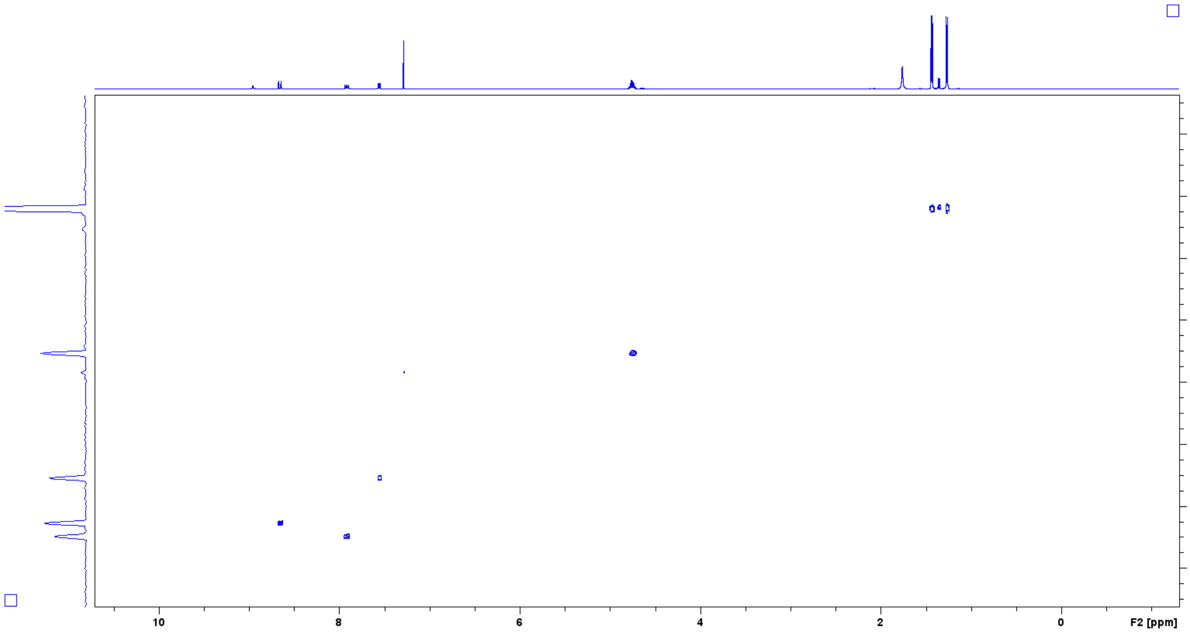

Figure S36: Full HSQC NMR spectrum for iPr4DPC (**2A**).

Moving the mass spectroscopy data, Figure S37 clearly shows the base peak at *m*/*z* 496.20 which corresponds to the $[M + H]^+$ species, as confirmed in Figure S38. A second major peak can be seen at 991.39, which corresponds to the $[2M + H]^+$ dimer species.

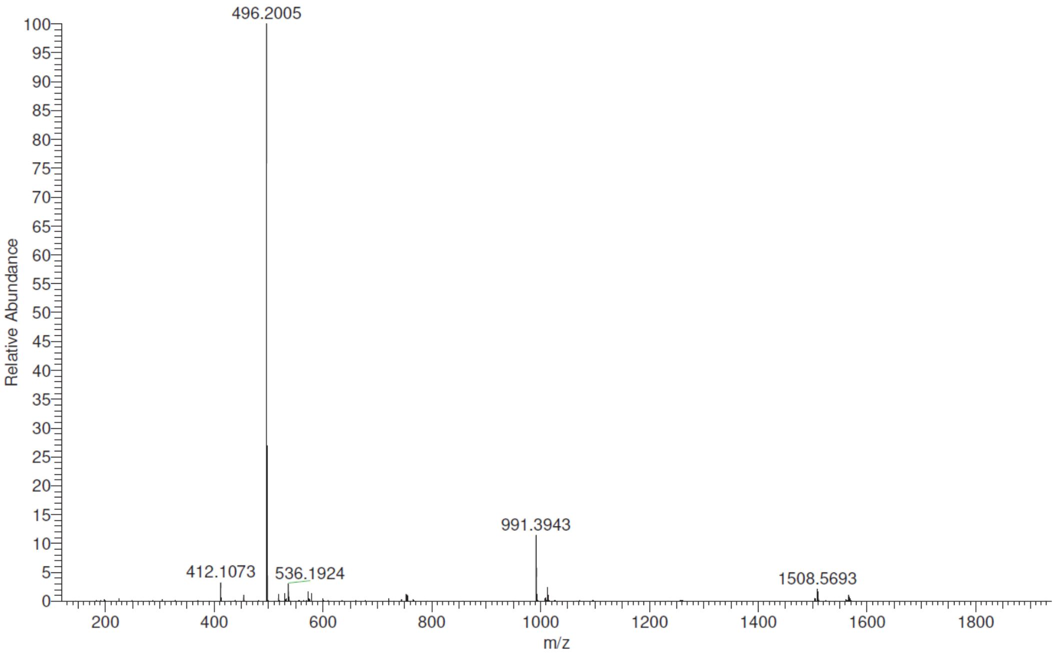

Figure S37: Full mass spectrum for iPr4DPC.

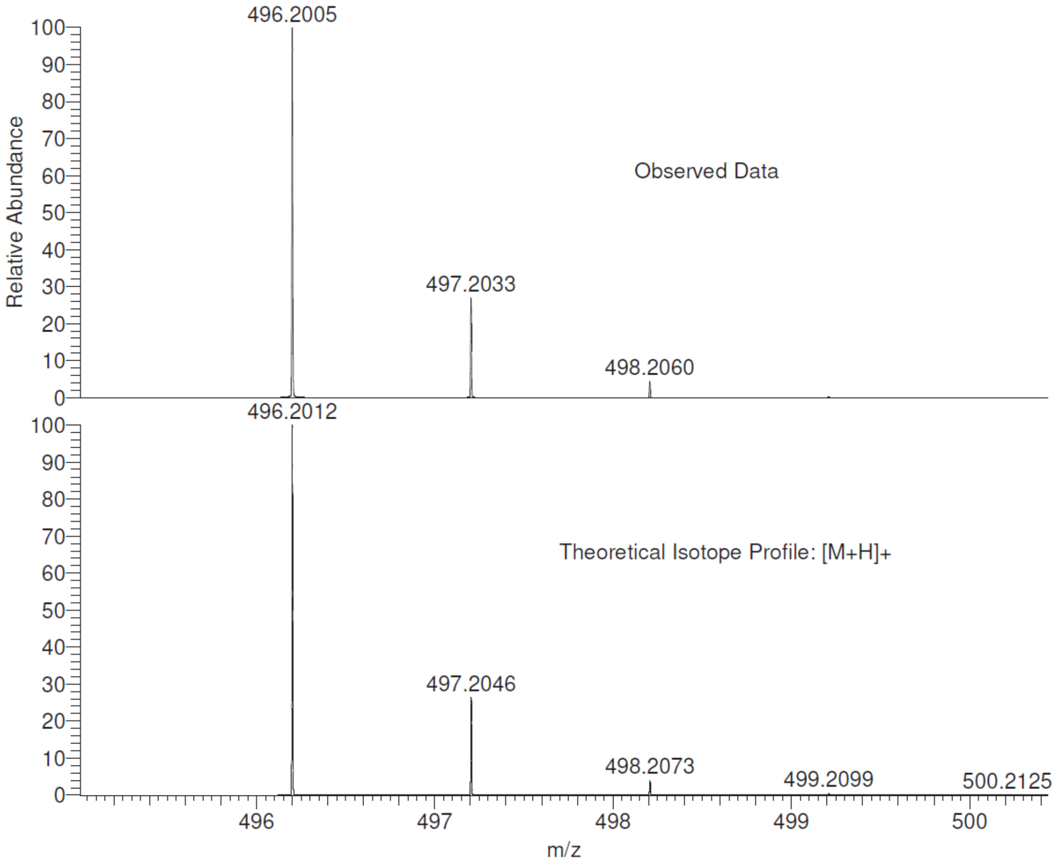

Figure S38: Mass spectrum for iPr$_4$DPC showing the observed data and the theoretical isotope profile for the [M + H]$^+$ species.

3,6-Diphosphono-9*H*-carbazole (H4DPC) (2B)

Having successfully obtained iPr4DPC, attention again shifted to obtaining the corresponding phosphonic acid, 3,6-diphosphono-9*H*-carbazole (H_4DPC). As with the amine, this was a relatively simple procedure and the product was obtained in yields between 87–98% when using the silylation and hydrolysis using TMSiBr, since hydrolysis using HCl again seemed to cleave the P–C bond. The solvent used for NMR was a 0.1 M solution of NaOH in D_2O.

Looking first at the ^{31}P NMR spectrum, as seen in Figure S39, there is a major signal at δ 12.91 ppm, which corresponds to the target product, H_4DPC. There is also a clear change in splitting pattern here, whereby a triplet is observed, though this can be attributed to coupling with the two nonequivalent aryl protons (H_b and H_c). There is also a minor signal upfield at δ 2.6 ppm which may correspond with hydrolysis of leftover triisopropyl phosphite, though this signal is relatively weak in comparison and can reasonably be ignored.

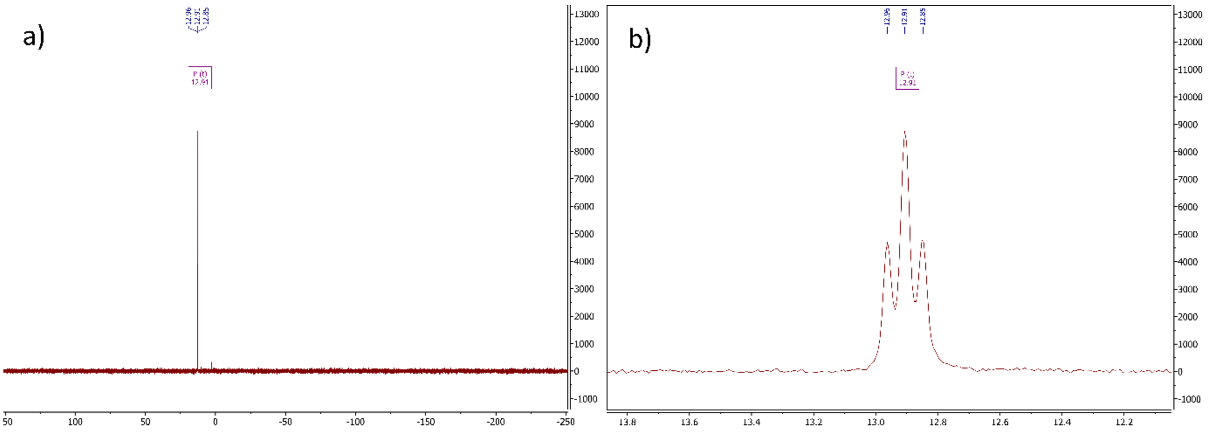

Figure S39: a) Full ^{31}P NMR spectrum, and b) zoomed ^{31}P NMR spectrum for H_4DPC (**2B**).

Moving next to the ^{1}H NMR spectrum as shown in Figure S41, it is clear that there are no signals corresponding to the isopropyl groups, thus the hydrolysis has been

successful, and there seem to be no signals that indicate other impurities. It is also clear that the signal observed previously for N–H has now disappeared, since a protic solvent is now being used.

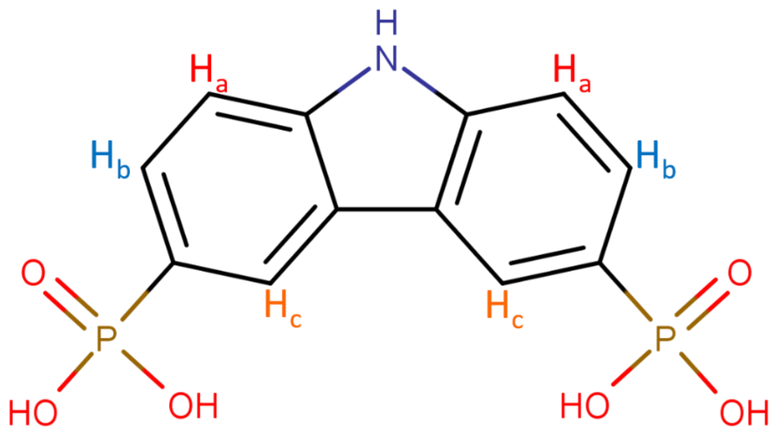

Figure S40: Chemical structure for H_4DPC with proton environments labelled H_a–H_c.

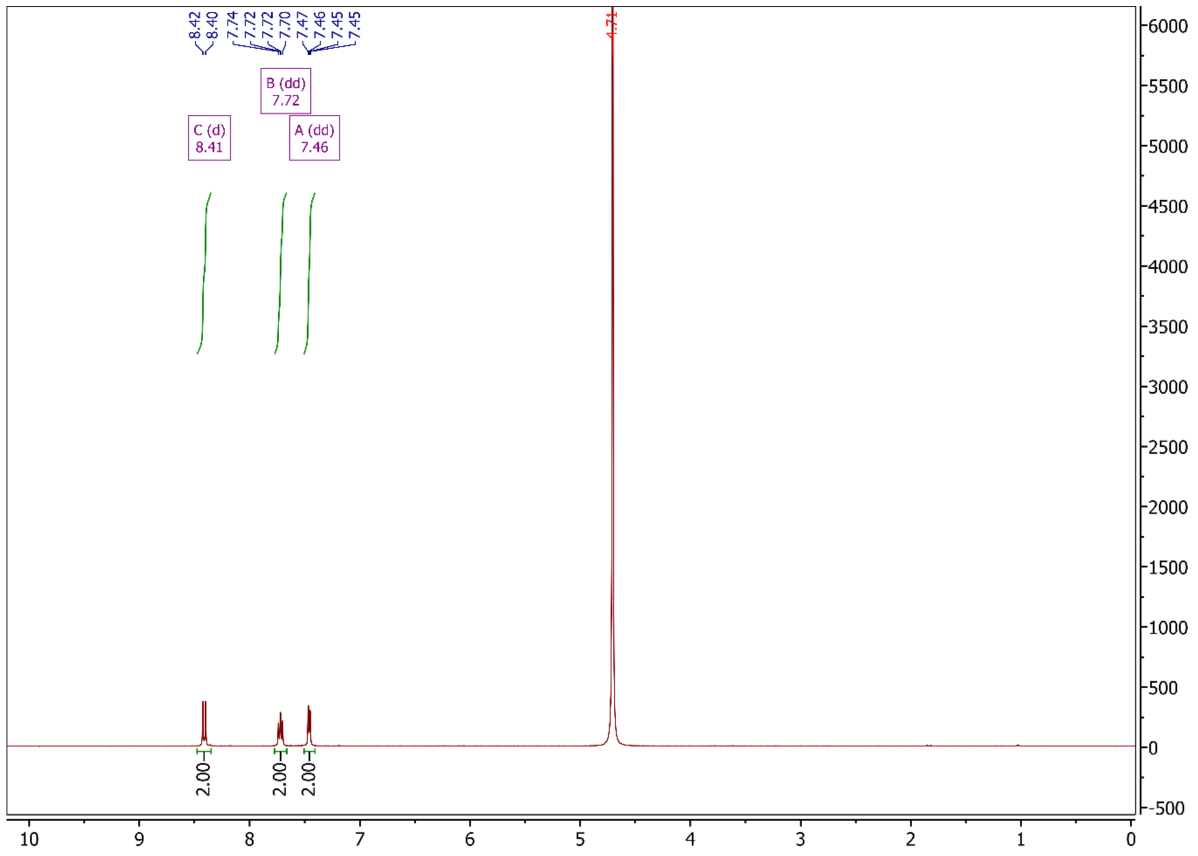

Figure S41: Full ^1H NMR spectrum for H_4DPC (**2B**).

Taking a closer look at the aromatic region, as seen in Figure S42, the signals present can be assigned exactly as they were for iPr4DPC, whereby protons labelled $H_{a–c}$ correspond to signals A–C respectively. One clear difference, however, is the less resolved nature of the spectrum, which causes overlap of signals. This is immediately clear for the signal at δ 7.72 ppm, which was previously classified as a doublet of doublets of doublets (ddd), though now seems to be a triplet. This increased intensity of the central peak is caused by the overlap of multiple peaks which, when resolved, would represent the expected ddd splitting pattern as caused by coupling to the other aryl protons and phosphorus.

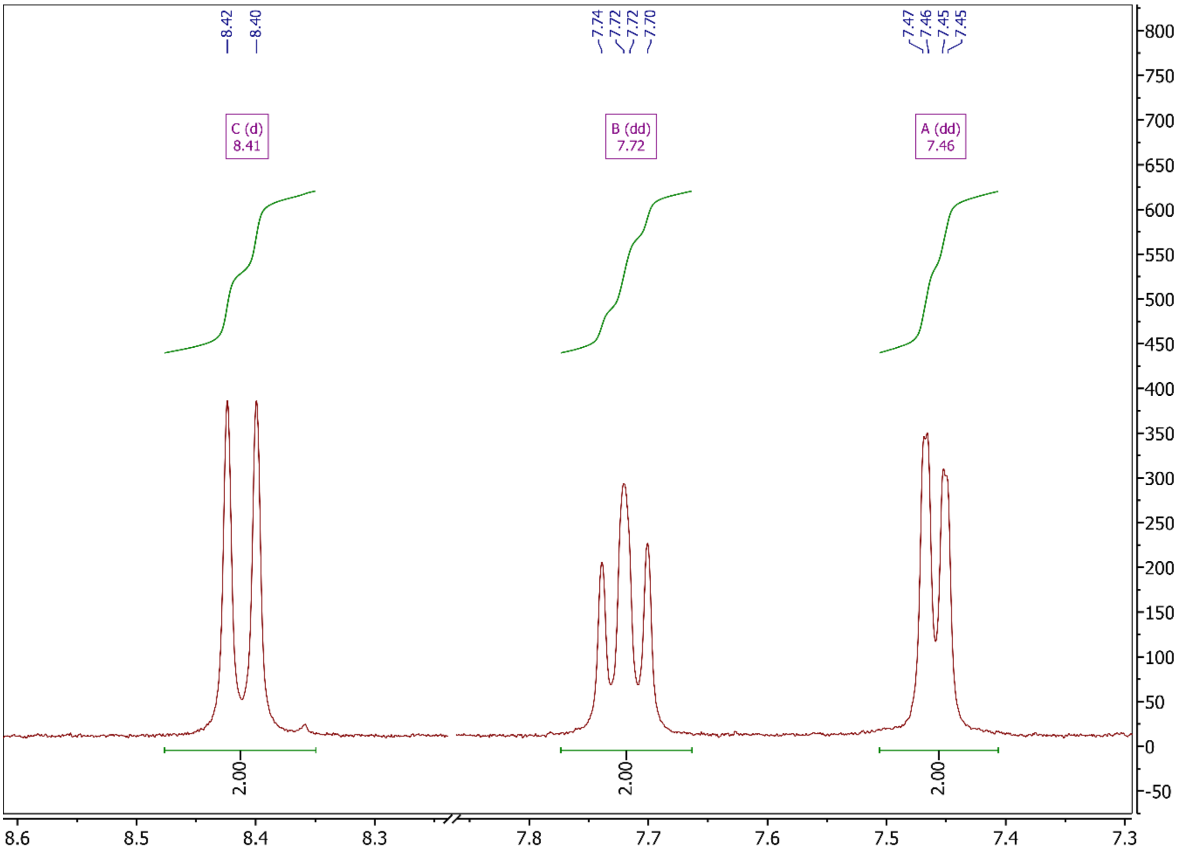

Figure S42: Aromatic region of ^{1}H NMR spectrum for H_4DPC (**2B**).

Further characterization using ^{13}C NMR was less successful, most likely due to the insolubility of H_4DPC and the subsequent inability to obtain a spectrum with sufficient peak intensities to accurately assign peaks, at least fully. Looking at Figure S44, it is clear that some of the potential signals in the area of interest are barely above the rather noisy background.

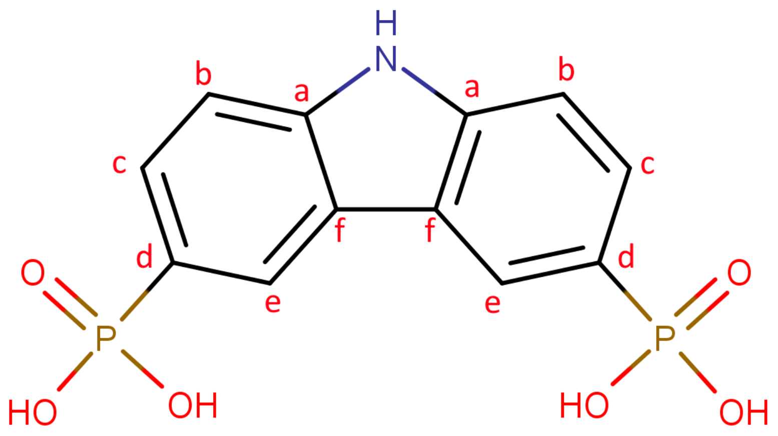

Figure S43: Chemical structure of H_4DPC (**2B**) with carbon environments labelled a–f.

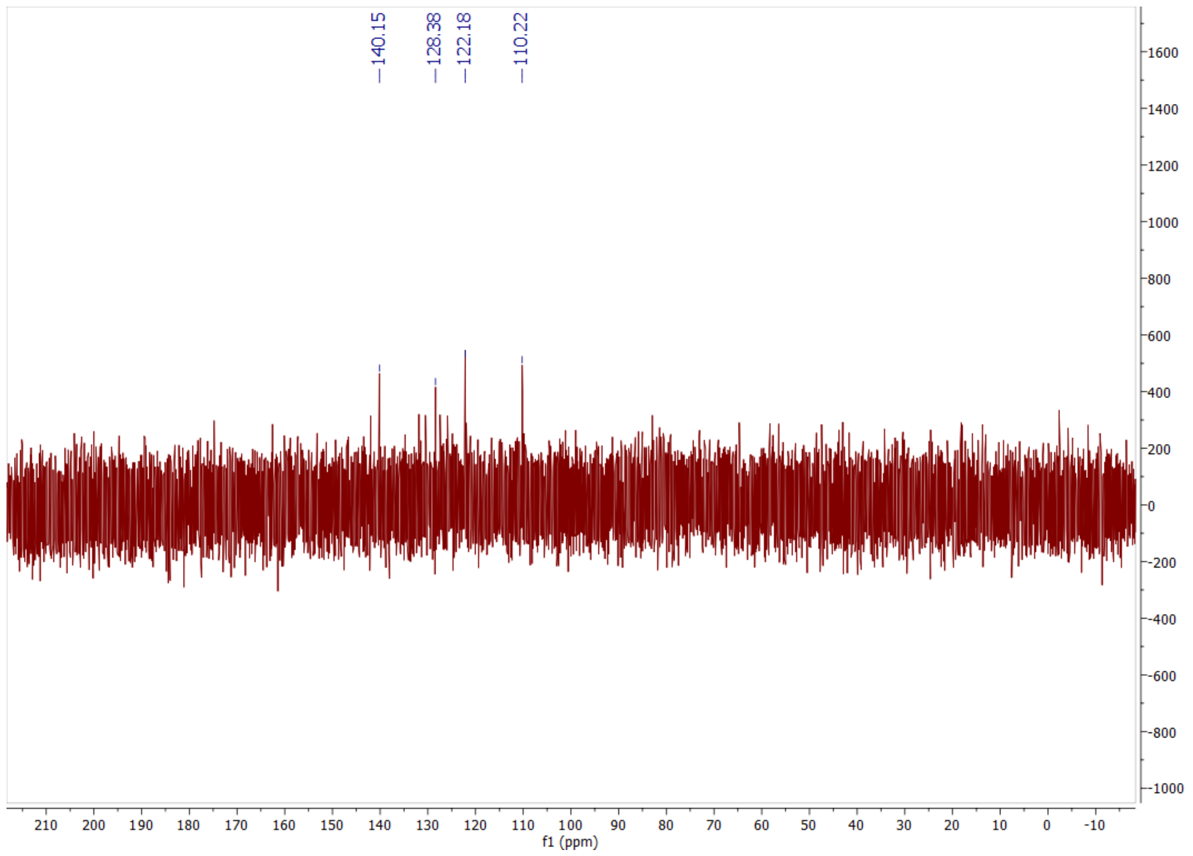

Figure S44: Full ^{13}C NMR spectrum for H_4DPC (**2B**).

Referring to the HSQC NMR spectra, as shown in Figure S45, it is possible to at least assign the peaks for carbons with bonded protons. With this in mind, it is clear that the ^{13}C NMR peaks at δ 128.38, δ 122.18, and δ 110.22 ppm can be assigned to the carbons labelled E, C, and B, respectively.

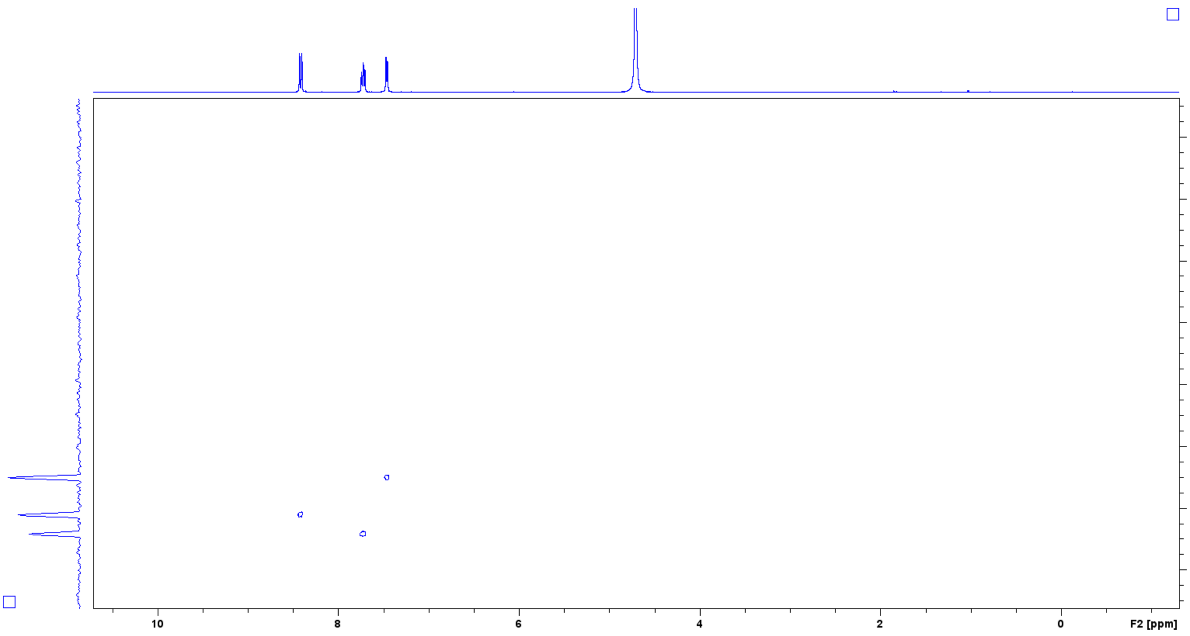

Figure S45: Full HSQC NMR spectrum for H_4DPC (**2B**).

Looking at the mass spectral data, Figure S46 clearly shows the base peak at *m*/*z* 162.50 ppm corresponding to the $[M - 2H]^{2-}$ species and can be seen in more detail in Figure S47. Another peak is shown at *m*/*z* 325.99, corresponding to the $[M - H]^{-}$ species.

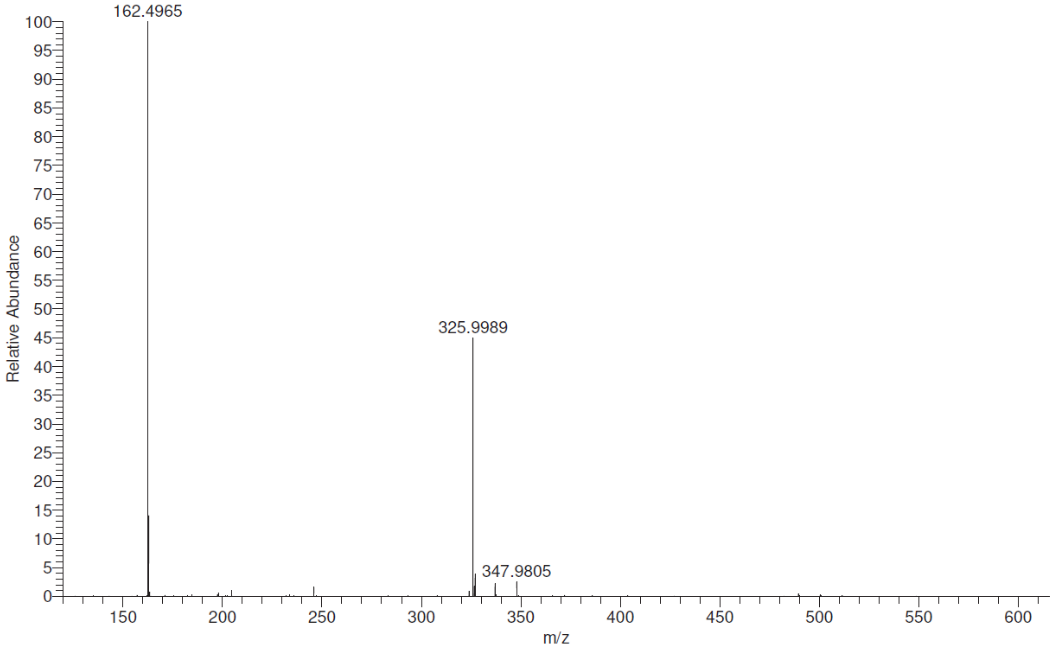

Figure S46: Mass spectrum for iPr_4DPC (**2B**).

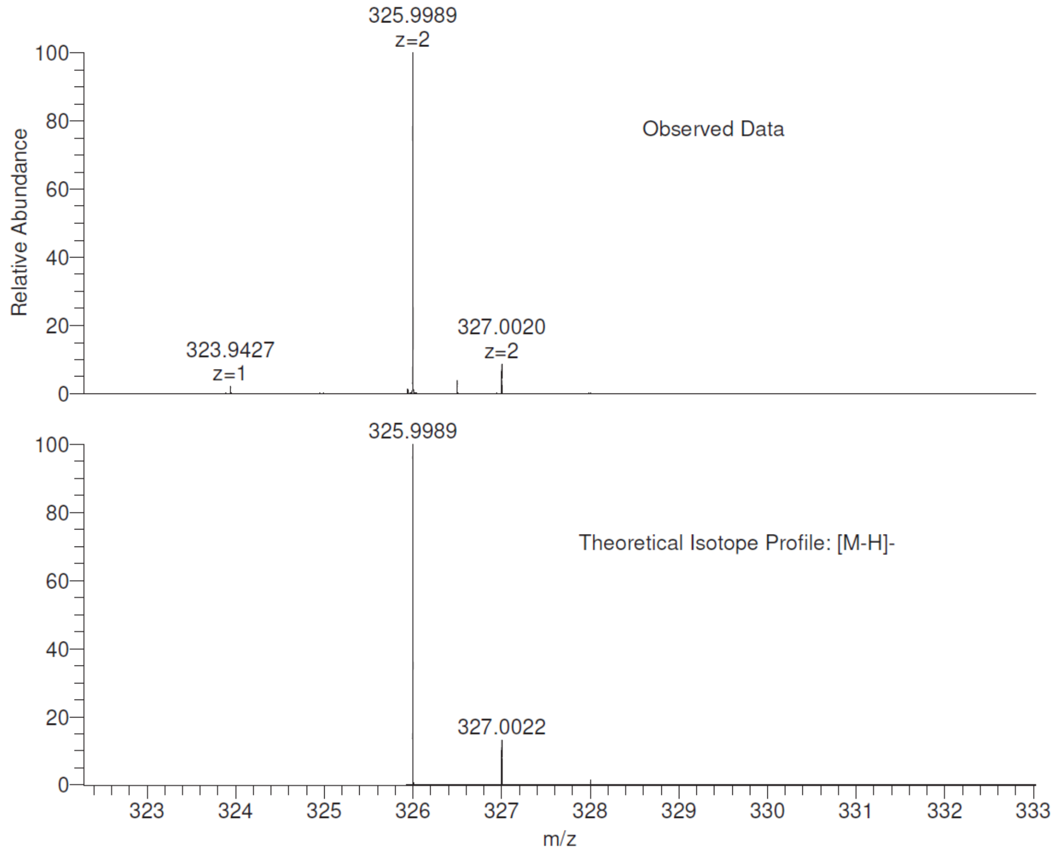

Figure S47: Mass spectrum for iPr_4DPC showing the observed data and the theoretical isotope profile for the $[M-H]^-$ species.

4-Diisopropylphosphono-*N*-(4-diisopropylphosphonophenyl)-*N*-phenylaniline [iPr4DPPA] (3A)

The third synthetic target obtained was 4-diisopropylphosphono-*N*-(4-diisopropyl-phosphonophenyl)-*N*-phenylaniline (iPr4DPPA, **3A**). As with the two previous linkers, this synthesis was also straightforward and could be achieved in yields above 80%. The procedure itself was essentially identical, with a simple workup in hexane to obtain the linker. The solvent used for NMR was $CDCl_3$.

Looking first at the ^{31}P NMR spectrum, see Figure S48, we see just a single signal which can be attributed to the target product, iPr_4DPPA. Taking a closer look at the signal, it seems that the splitting observed for the two previous linkers is not present, though this might be due to a reduction in the signal to noise ratio.

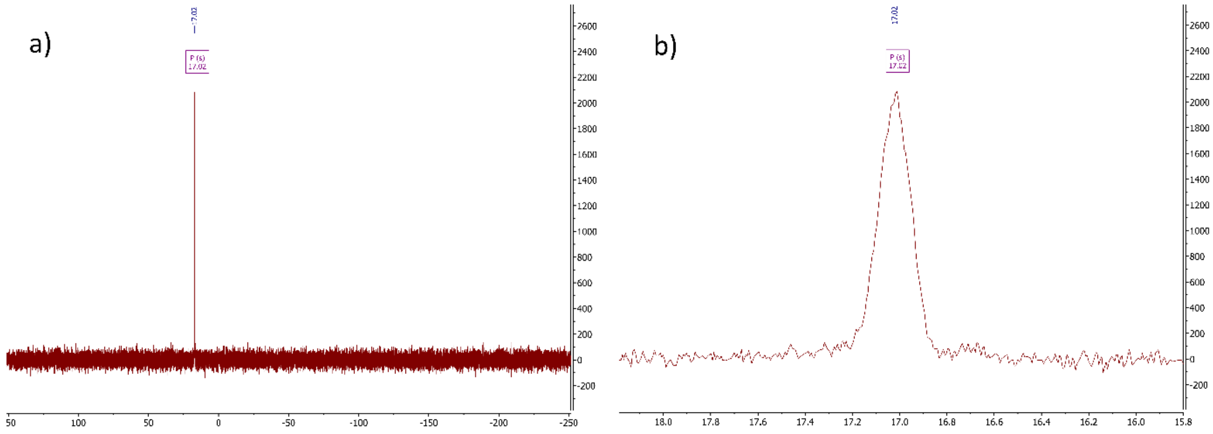

Figure S48: a) Full ^{31}P NMR spectrum, and b) zoomed ^{31}P NMR spectrum for iPr_4DPPA (**3A**).

Moving to the ^{1}H NMR spectrum, as shown in Figure S50, we again see the impurities suspected to be either triisopropyl phosphite and/or isopropyl bromide, as was indicated for iPr_4DPC. Beyond this, there are no other unexpected signals, and the only other features of note are the slightly unresolved signals in the aromatic region.

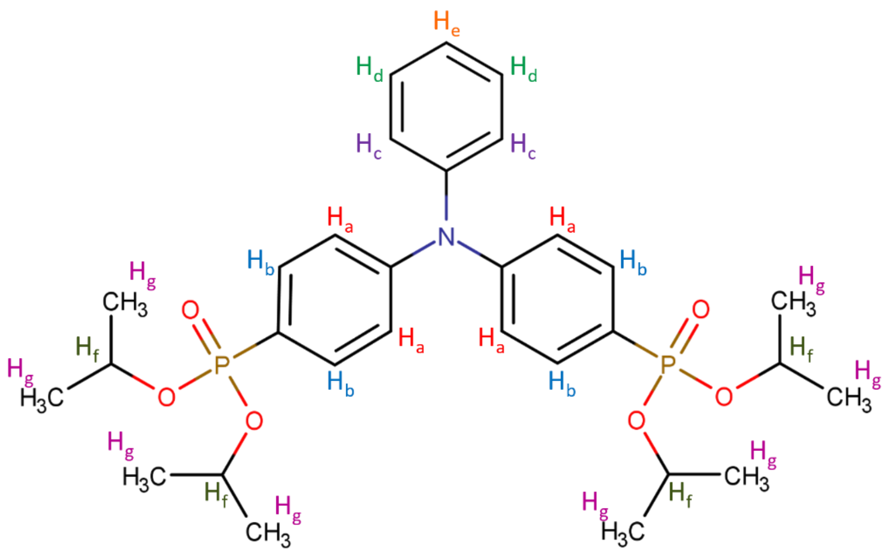

Figure S49: Chemical structure for iPr$_4$DPPA (**3A**) with proton environments labelled H_a–H_g.

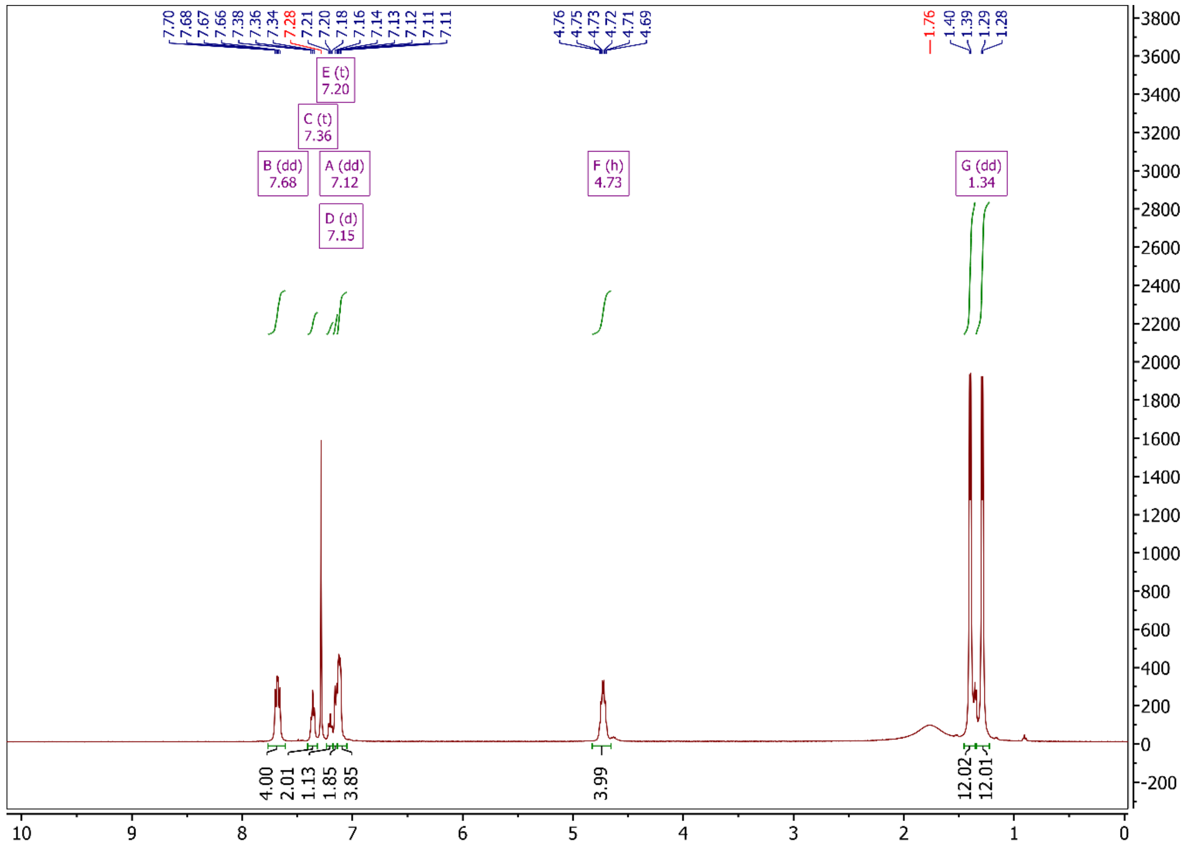

Figure S50: Full ^1H NMR spectrum for iPr$_4$DPPA (**3A**).

Zooming in on the aromatic region, as seen in Figure S51, it is possible to identify five signals in total. The first of these signals has a δ-shift of 7.86 ppm, which can be assigned similarly to the iPr_4BPA linker, whereby the peak corresponds to protons nearest to phosphorus on the phenyl rings, labelled H_b in Figure S49, again indicated by the large coupling constants. The integration also indicates that it at least belongs to the rings containing phosphonate groups. The next signal, at δ 7.36 ppm, has been assigned to the protons labelled H_c on the phenyl ring with no phosphonate group. The splitting on the spectrum shows a triplet, though you might expect a doublet of doublets for the associated proton, therefore it is likely that this is what we are seeing, simply unresolved. Next, we see the solvent peak at δ 7.28 ppm. Moving upfield, we see a relatively easy to assign the signal at δ 7.20 ppm, and in this case has been attributed to the proton labelled H_e and is supported by the 1.13 integration value. The final two signals in the aromatic region, while not fully resolved, can be assigned and the splitting can also be identified. The first one, at δ 7.15 ppm, appears to be a simple doublet, and integrates approximately to two, and should therefore be assigned to the remaining protons on the non-phosphonate-containing ring labelled H_d. By process of elimination, this leaves the final aromatic signal at δ 7.12 ppm to be assigned to the protons labelled H_a, though this assignment is supported by the doublet of doublet splitting pattern as well as the integration of approximately four.

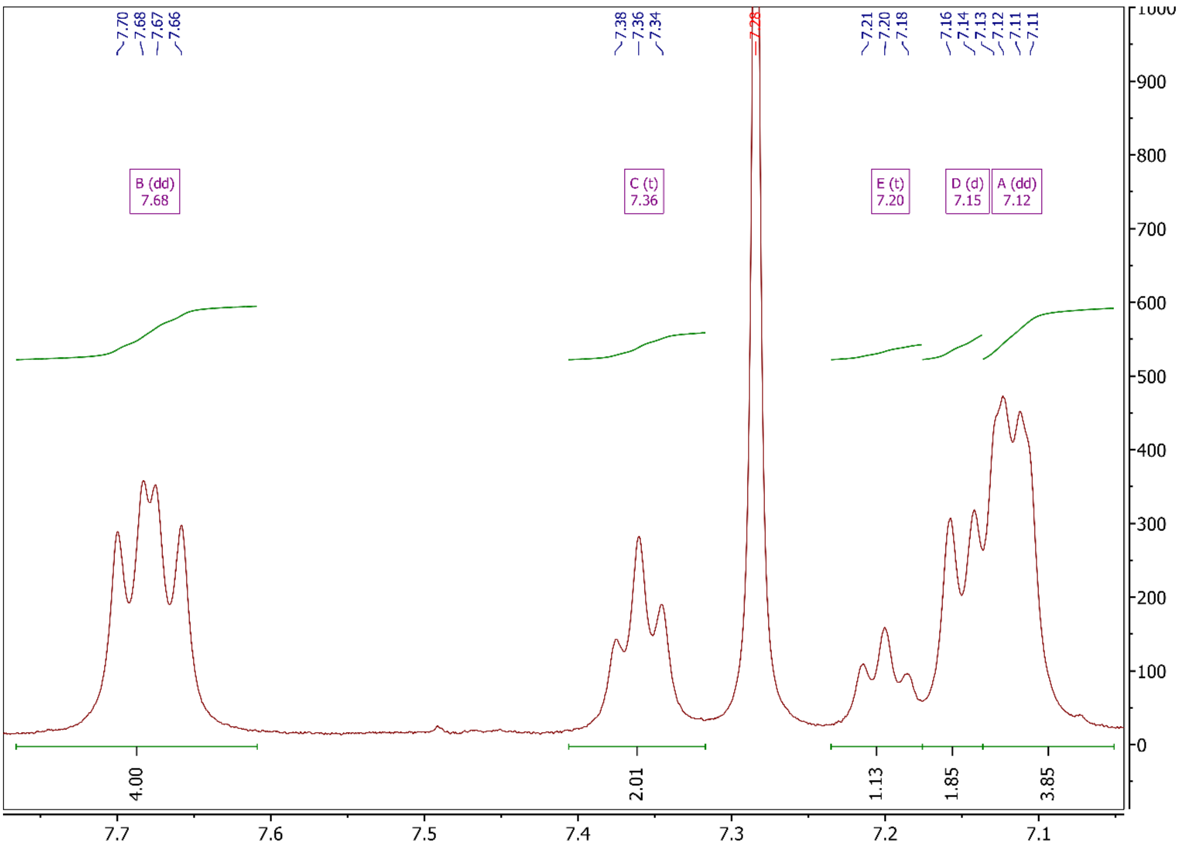

Figure S51: Aromatic region of ^{1}H NMR spectrum for iPr$_4$DPPA (**3A**).

Moving to the signal at δ 4.73 ppm, as shown in Figure S52, we see what initially looked like a sextet, though as we have previously explored, is most likely an unresolved doublet of septets, as it would be expected for the methine moiety on the isopropyl groups, labelled H_f in Figure S49. The integration of four is also in favour of this assignment.

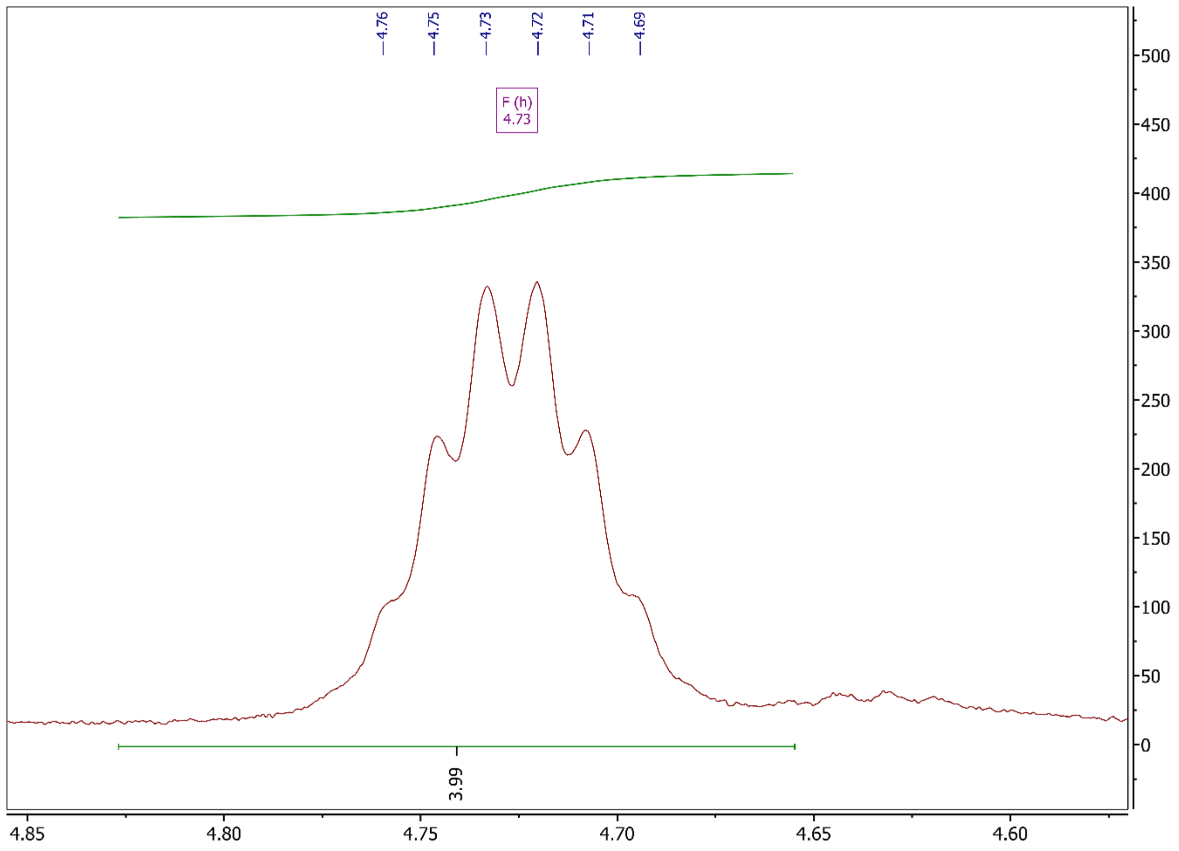

Figure S52: Methine region of ^{1}H NMR spectrum for iPr$_4$DPPA (**3A**).

The final signal, as shown in Figure S53, appears at δ 1.34 ppm as a pair of doublets, as was established for iPr$_4$BPA and iPr$_4$DPC, and corresponds to the methyl groups belonging to the isopropyl moiety. The integration for each of the doublets is 12, together totaling 24, as expected.

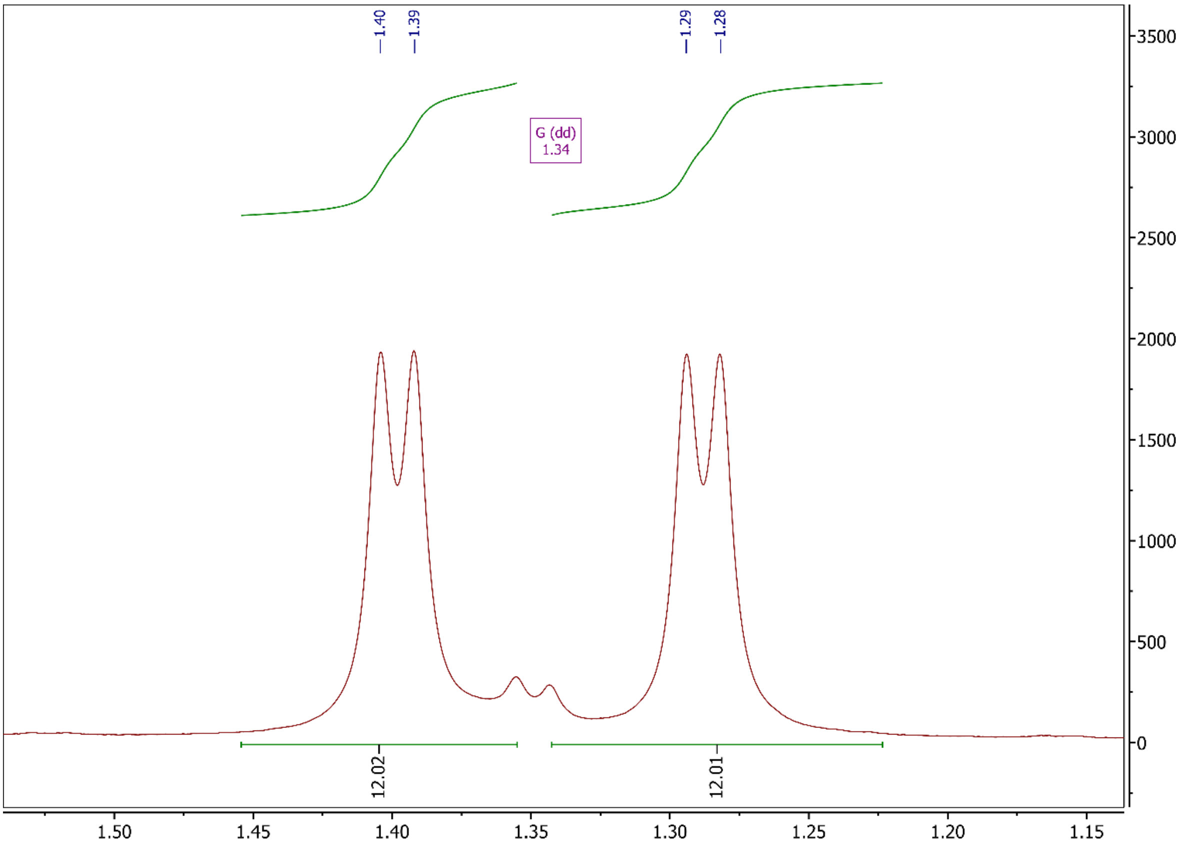

Figure S53: Methyl region of ^{1}H NMR spectrum for iPr$_4$DPPA (**3A**).

Moving on to the ^{13}C NMR spectrum, as shown in Figure S55, we can immediately assign the methyl (i) and methine (h) carbons to the signals at δ 24.02 ppm and δ 70.63 ppm, respectively. Moving downfield, things start to get slightly more complicated. The first two signals at δ 150.25 ppm and δ 145.08 ppm have both been assigned to the carbon atoms bonded to nitrogen, labelled (a) in Figure S48, with one representing the rings containing phosphonate groups and the other representing the ring with no phosphonate group. Following this, we have the signal at δ 133.03 ppm, which has been assigned to the carbons labelled (c). Next is the signal at δ 129.84 ppm, which has been assigned to the carbons on the non-phosphonate-containing ring, labelled (e). The next signal, at δ 126.54 ppm, has been assigned to the carbons directly bonded to phosphorus, labelled (d). The next two signals at δ

125.27 ppm and δ 123.95 ppm, which are of slightly lower intensity, have been assigned to the carbons on the non-phosphonate-containing ring, labelled (f) and (g), respectively. The final signal on the spectrum, δ 122.46 ppm, has been assigned to the carbons in the β-positions, relative to nitrogen, on the phosphonate containing rings, labelled (b).

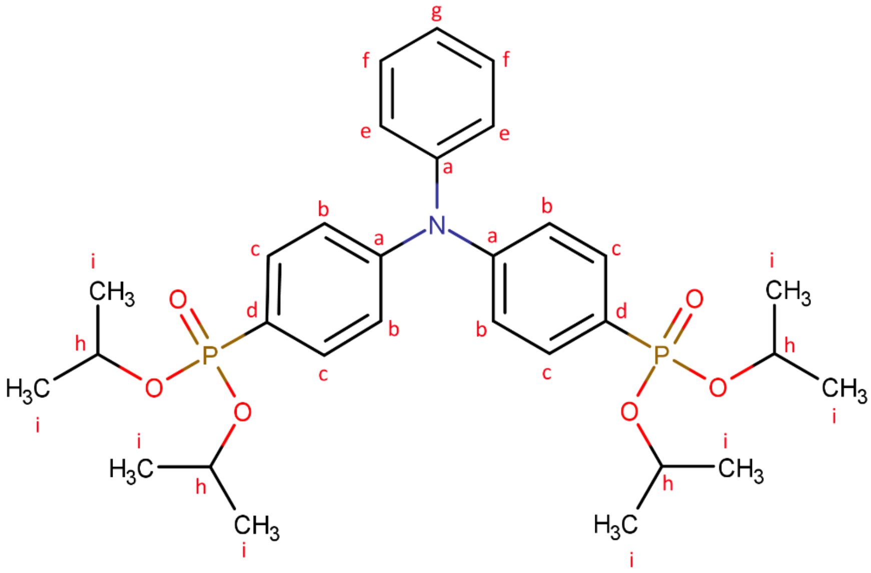

Figure S54: Chemical structure for iPr4DPPA (**3A**) with carbon environments labelled a–i.

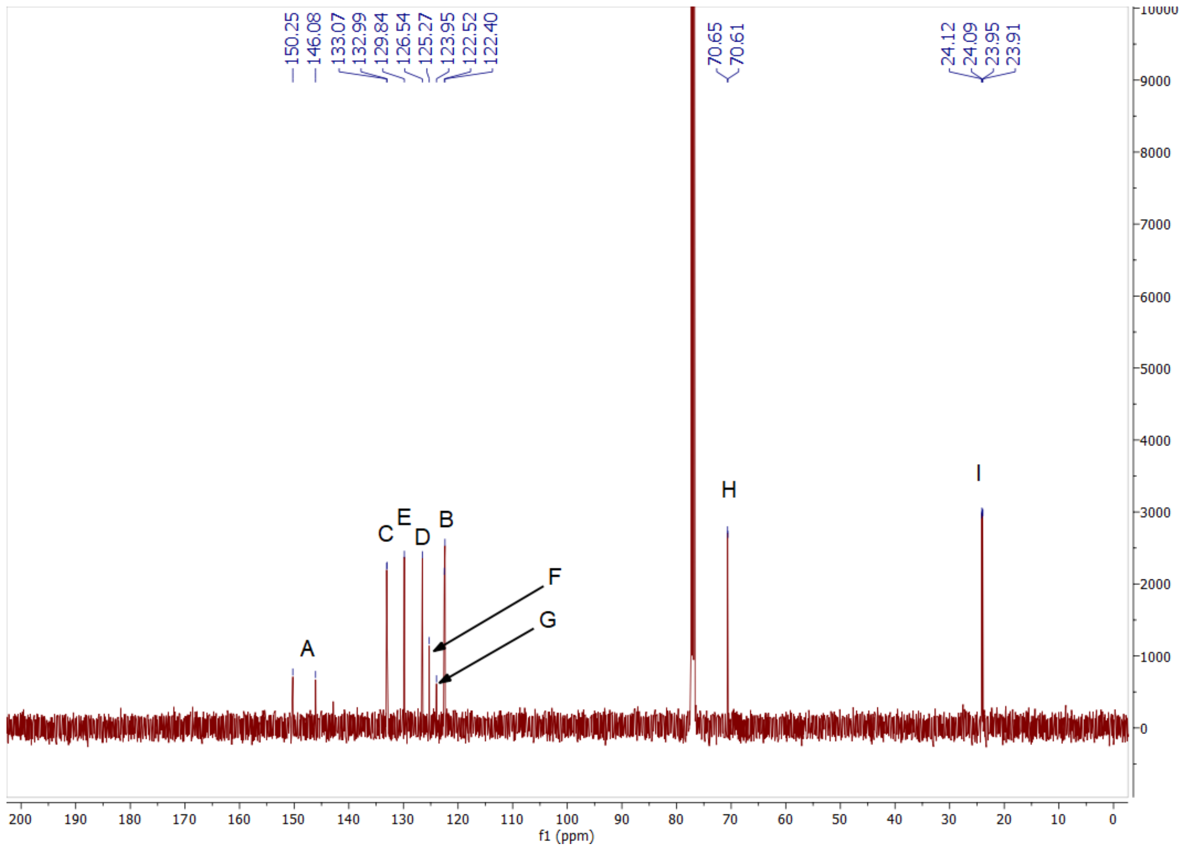

Figure S55: Full ^{13}C NMR spectrum for iPr_4DPPA (**3A**).

Lending support for these assignments as well as those for the proton NMR, is the HSQC NMR spectrum shown in Figure S56 and S57, in which the signals have been labelled 1–5 for clarity. The signals show the correlations between C_c–H_b (1), C_e–H_c (2), C_g–H_e (3), C_f–H_d (4), and C_b–H_a (5).

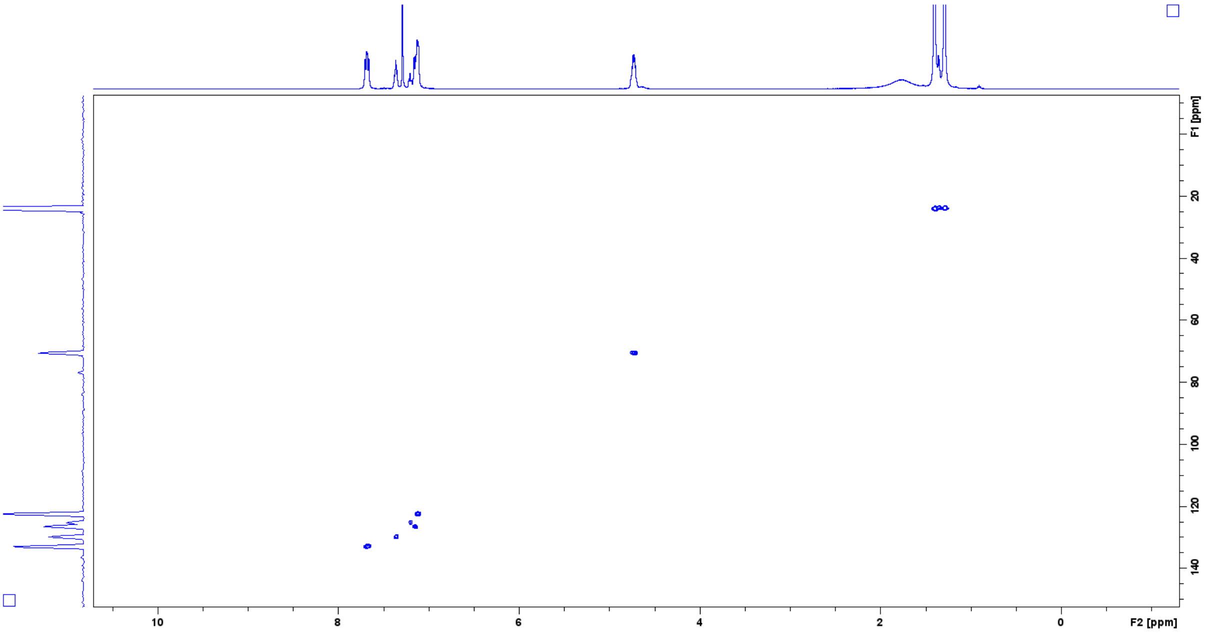

Figure S56: Full HSQC NMR spectrum for iPr4DPPA (**3A**).

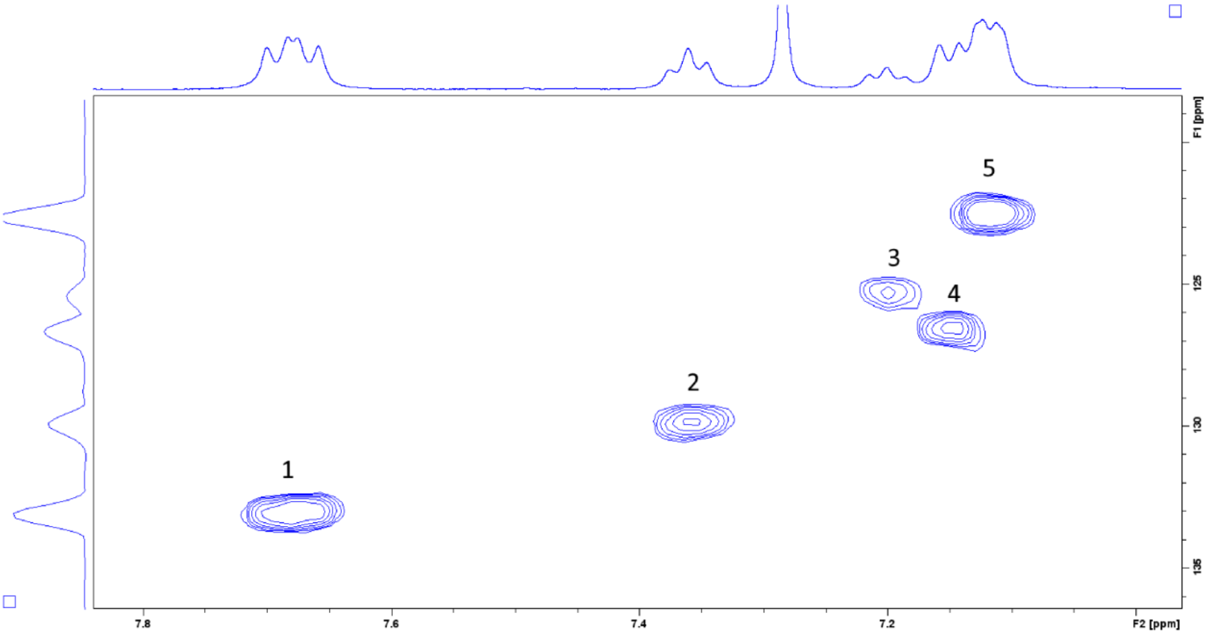

Figure S57: Zoomed HSQC NMR spectrum for iPr4DPPA (**3A**).

Mass spectral analysis was carried out, and Figure S58 clearly shows the base peak at *m*/*z* 574/25, which corresponds to the $[M + H]^+$ species. This can be seen in greater detail in Figure S59, which compares the theoretical isotope profile with observed data, showing a good match between the two.

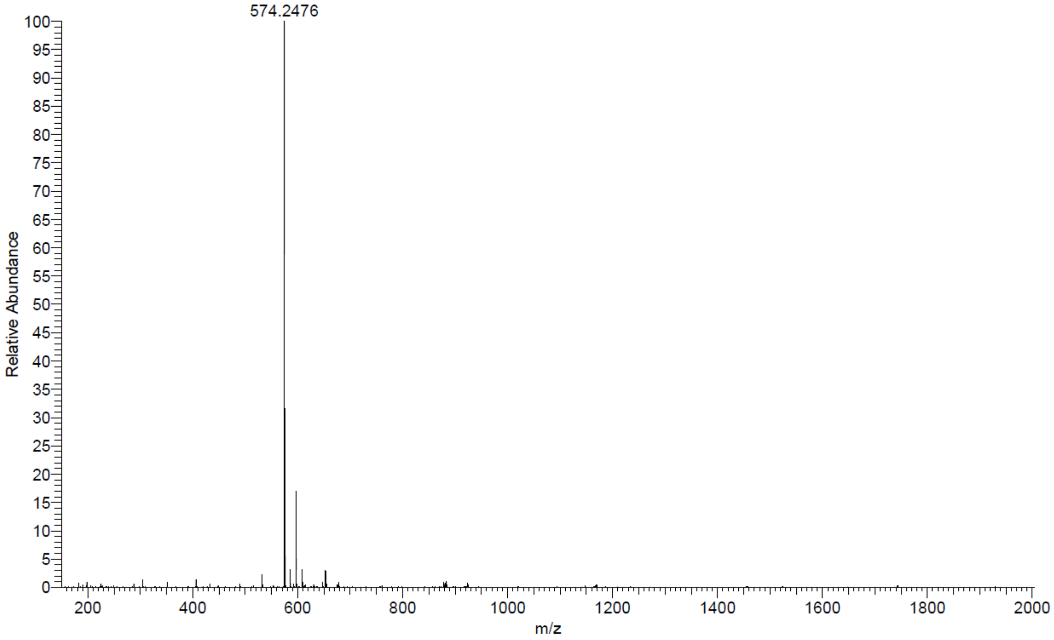

Figure S58: Full mass spectrum for iPr4DPPA (**3A**).

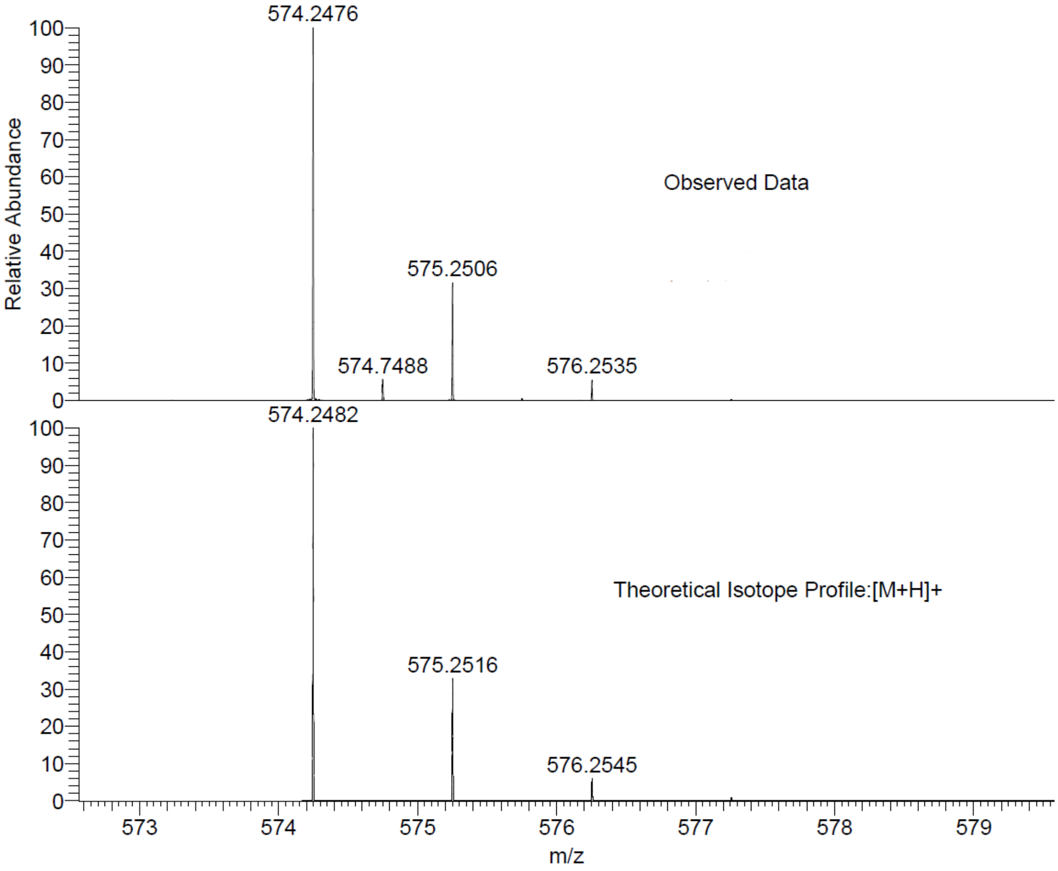

Figure S59: Mass spectrum for iPr4DPPA (**3A**) showing the observed data and the theoretical isotope profile for the $[M + H]^+$ species.

4-Phosphono-*N*-(4-phosphonophenyl)-*N*-phenylaniline [H_4DPPA] (3B)

Although a rather repetitive statement at this point, the hydrolysis of iPr$_4$DPPA to obtain H_4DPPA was, as was the case for the other two linkers, a relatively simple procedure, and pretty reasonable yields of between 70–95% could be achieved. Unlike the previous two, this linker was not assessed for its suitability for hydrolysis using HCl, instead opting to go straight for the less harsh procedure involving silylation through the use of TMSiBr. The solvent used for NMR was a 0.1 M solution of NaOH in D_2O.

Starting with the ^{31}P NMR spectrum, as shown in Figure S60, we can immediately see the presence of only one signal, indicating relative purity with regards to phosphorus-containing compounds. We can already observe the loss of the isopropyl groups here from the huge change in the splitting pattern. Here, we see only a triplet, likely caused by coupling with the aromatic protons, whereas the pattern for the ester presented a much more complicated and much less resolved pattern due to the amount of coupling taking place.

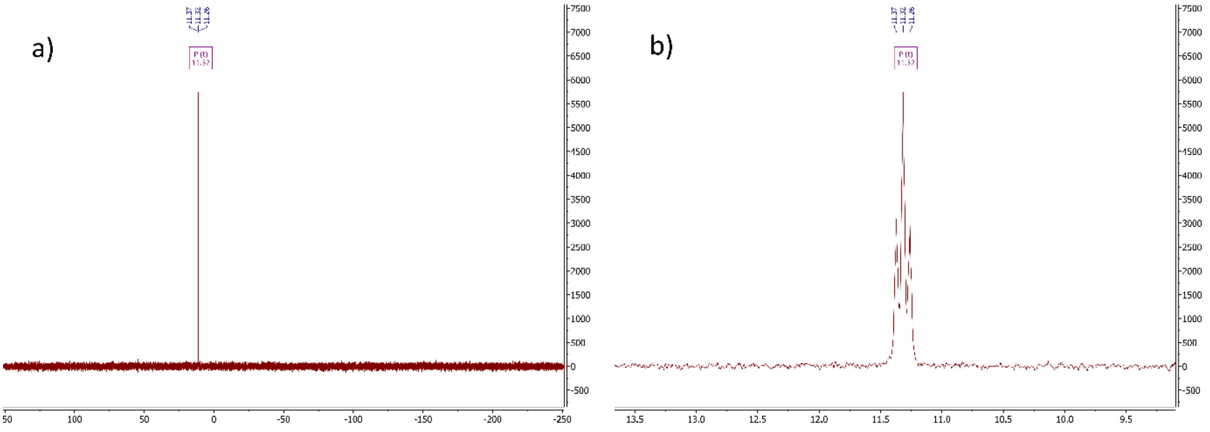

Figure S60: a) Full ^{31}P NMR spectrum, and b) zoomed ^{31}P NMR spectrum for H_4DPPA (**3B**).

Moving to the ^{1}H NMR spectrum as shown in Figure S62, we can immediately confirm the loss of the isopropyl groups through the lack of methine and methyl signals upfield. Beyond this, the only potential impurity shown at δ 0.0 ppm, which can be attributed to phosphorus acid (H_3PO_3), though the amount present must be quite small as it is not seen in ^{31}P NMR spectrum and could easily be removed through further washing in small amounts of water or ethanol.

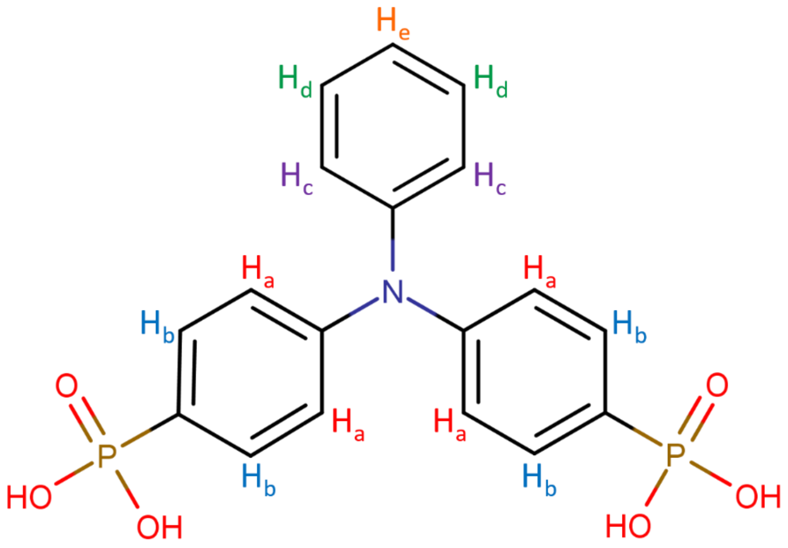

Figure S61: Chemical structure for H_4DPPA (**3B**) with proton environments labelled H_a–H_e.

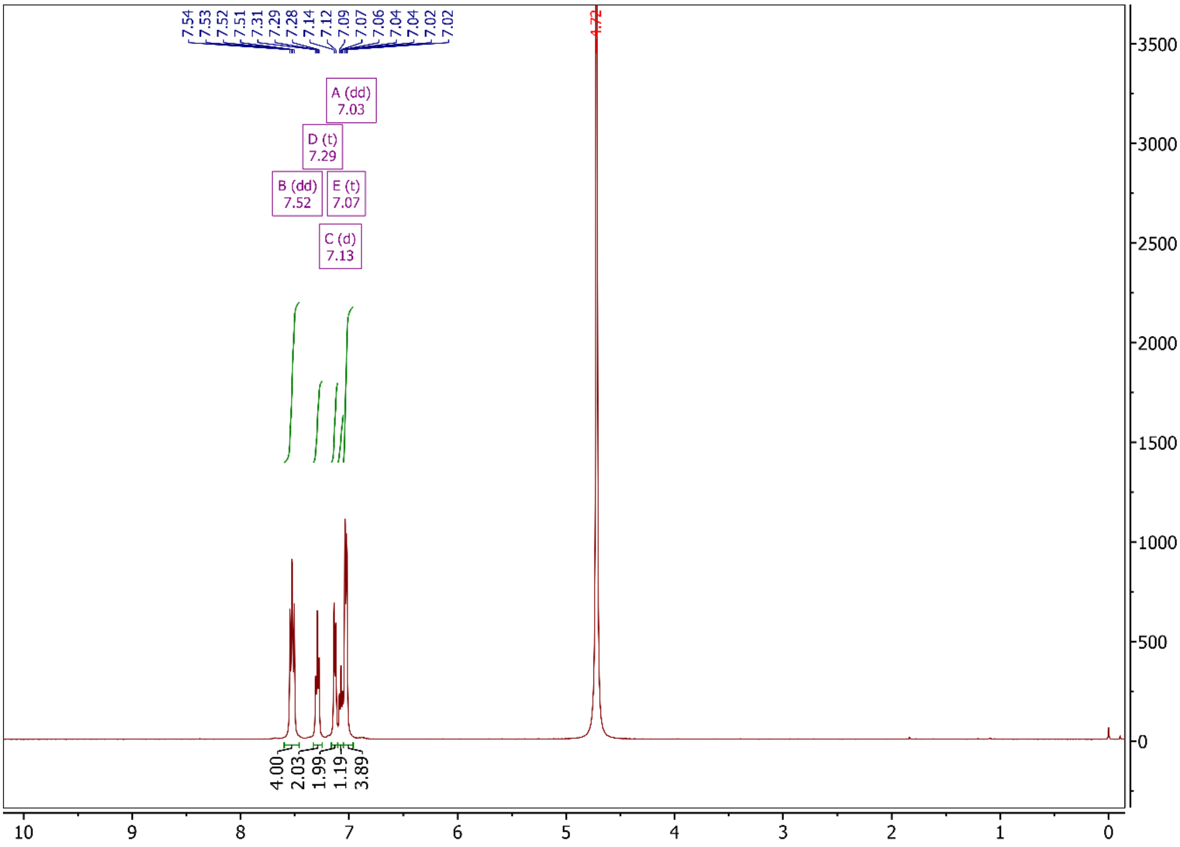

Figure S62: Full ^{1}H NMR spectrum for H_4DPPA (**3B**).

Focusing on the aromatic region, as shown in Figure S63, and comparing it to that of the ester in Figure S51, the assignment of the signals is clear, though there is one striking difference, which is that two of the signals, C and E, have switched places on the spectrum. Turning back to the assignment of the signals, as was the case for the ester, the signal at δ 7.52 ppm has been assigned to the protons nearest to phosphorus, labelled H_b. The next signal, at δ 7.29 ppm, has been assigned to the protons labelled H_d on the non-phosphonate-containing ring. The signal at δ 7.13 ppm has been assigned to the protons nearest nitrogen on the non-phosphonate-containing ring, labelled H_c. The next signal, and generally the easiest to assign due to the integration, has been assigned to the proton labelled H_e on the non-phosphonate-containing ring. This leaves the final signal at δ 7.13 ppm, which through

process of elimination, supported by splitting pattern and integration, be attributed to the protons labelled H_a.

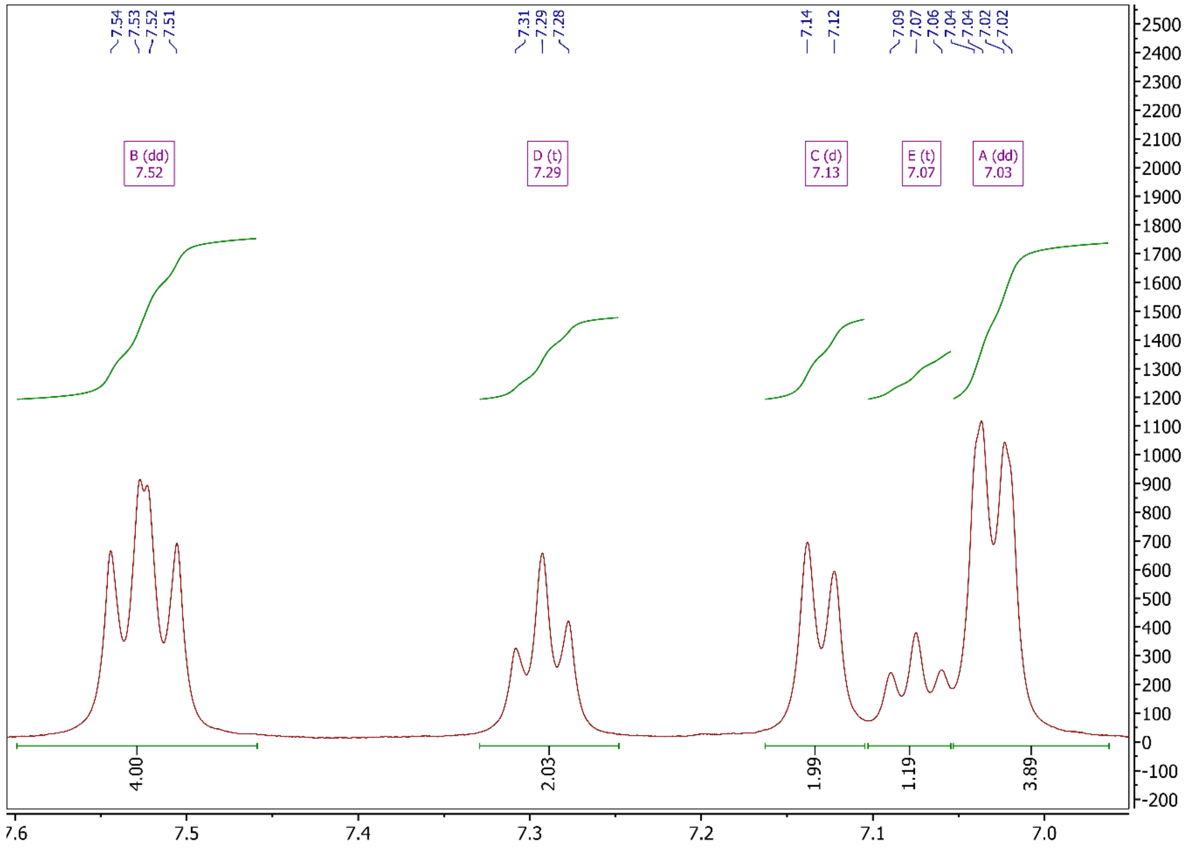

Figure S63: Aromatic region of the ^{1}H NMR spectrum for H_4DPPA (**3B**).

As was the case for the ester, ^{13}C NMR, as shown in Figure S65, has proven insufficient in aiding full characterization of the linker. This is mostly due to the insolubility of the product, which has resulted in a poor signal-to-noise ratio. With this in mind, HSQC should help with assigning some of the ^{13}C NMR peaks. It should be noted here that the ^{13}C NMR spectrum clearly shows the lack of isopropyl group peaks.

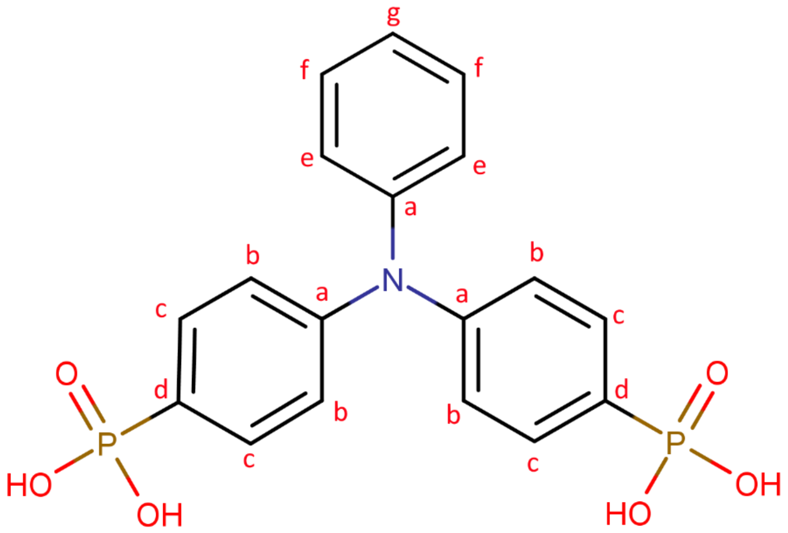

Figure S64: Chemical structure for H_4DPPA (**3B**) with carbon environments labelled a–g.

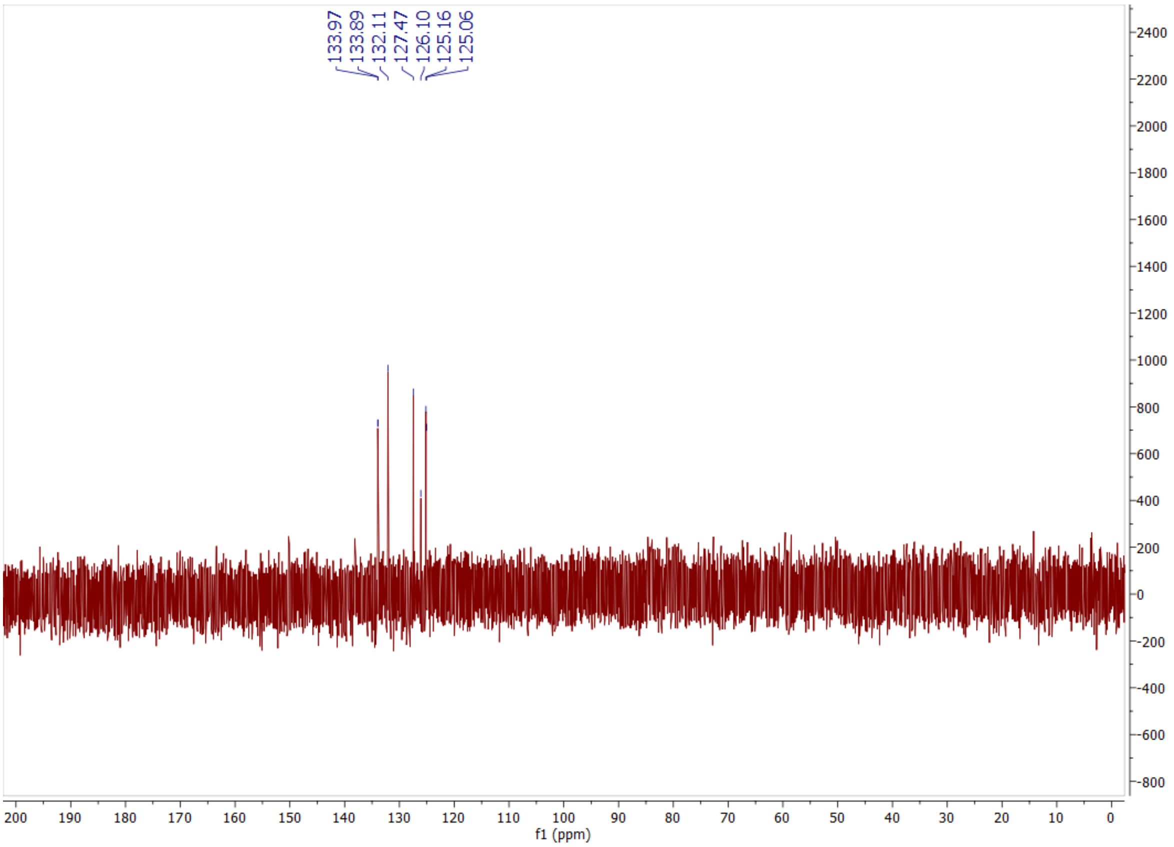

Figure S65: Full ^{13}C NMR spectrum for H_4DPPA (**3B**).

Looking at the HSQC NMR spectra in Figure S66, we can indeed see the switching of signals 3 and 4 when comparing with that of the ester in Figure S56. Beyond that, the assignments are identical, with the signals showing correlations between C_c–H_b (1), C_e–H_c (2), C_g–H_e (3), C_f–H_d (4), and C_b–H_a (5).

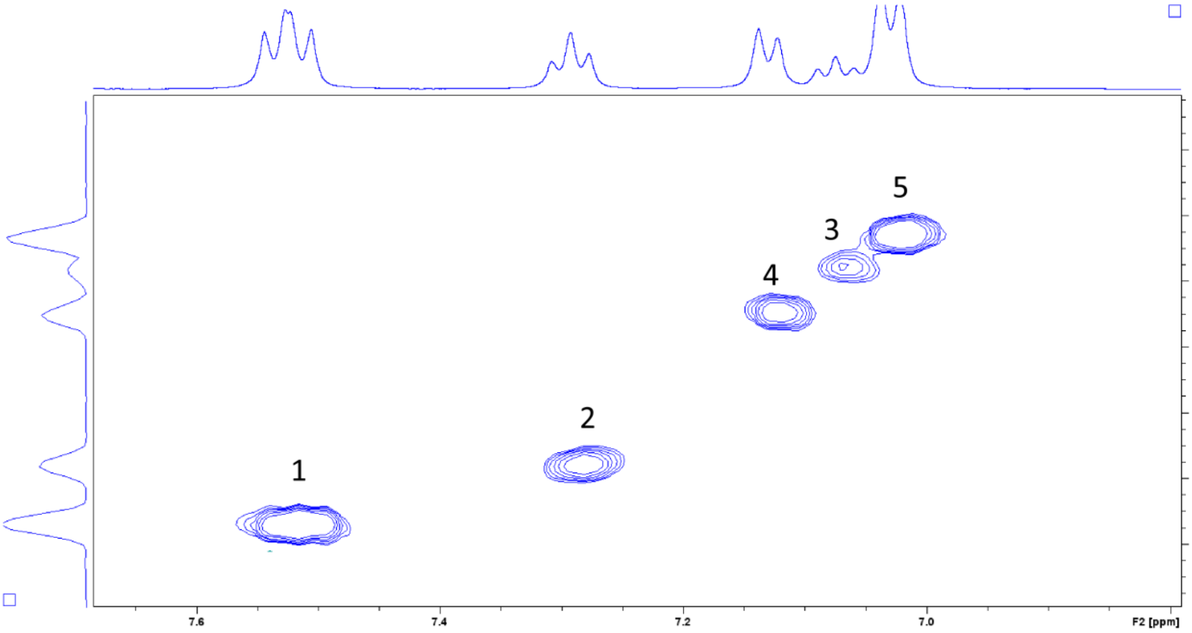

Figure S66: Full HSQC NMR spectrum for H_4DPPA (**3B**).

Mass spectral analysis was carried out, and Figure S67 clearly shows the base peak at *m*/*z* 201.52, which corresponds to the $[M-2H]^{2-}$ species. This can be seen in greater detail in Figure S68, which compares the theoretical isotope profile with observed data, shown a good match between the two.

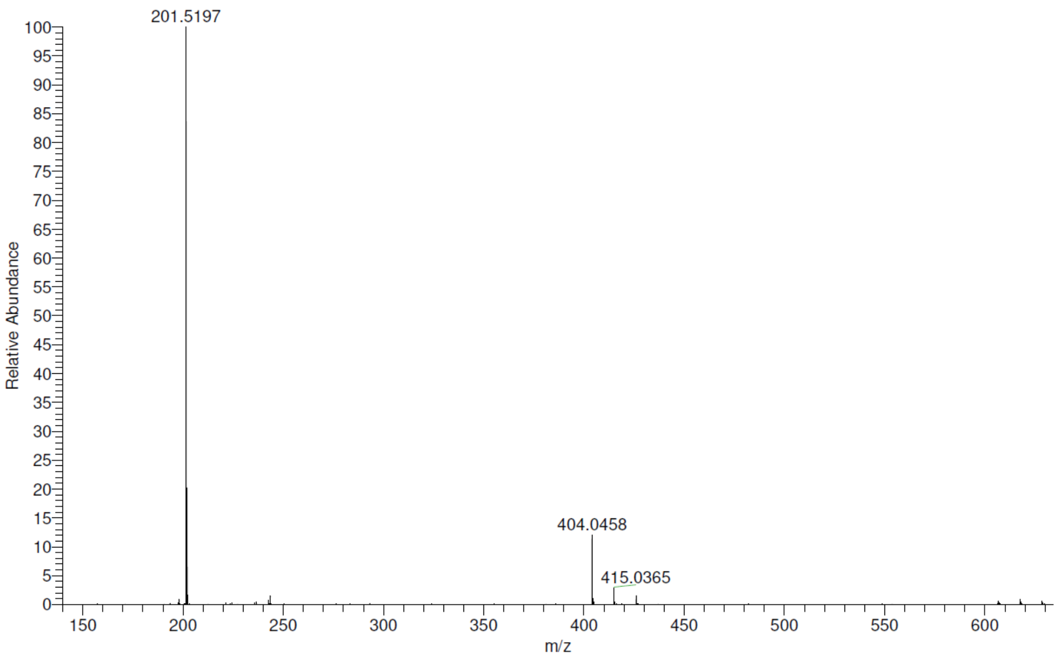

Figure S67: Full mass spectrum for H_4DPPA (**3B**).

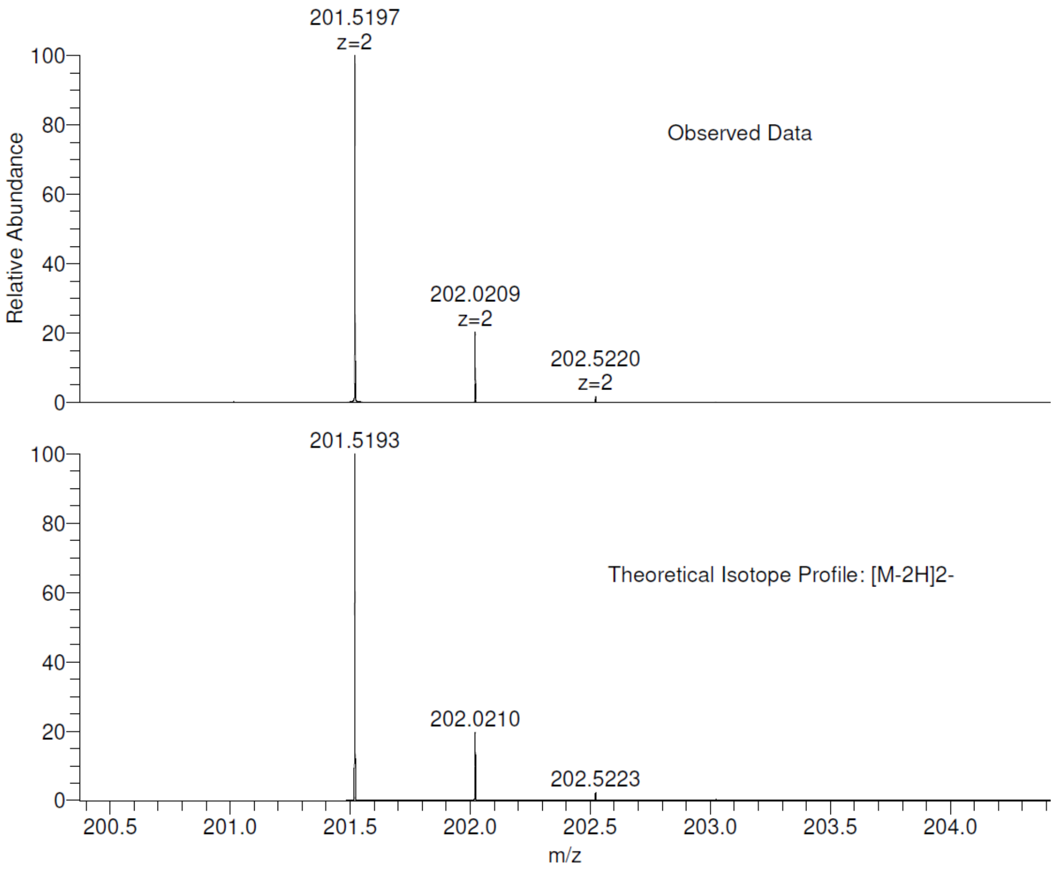

Figure S68: Mass spectrum for H_4DPPA (**3B**) showing the observed data and the theoretical isotope profile for the $[M - 2H]^{2-}$ species.

References

1. Taddei, M.; Costantino, F.; Vivani, R., *Eur. J. Inorg. Chem.* **2016,** *2016*, 4300-4309.

2. Sevrain, C. M.; Berchel, M.; Couthon, H.; Jaffres, P. A., *Beilstein J. Org. Chem.* **2017,** *13*, 2186-2213.
